# *Stenotrophomonas maltophilia* uses a c-di-GMP module to sense the mammalian body temperature during infection

**DOI:** 10.1371/journal.ppat.1012533

**Published:** 2024-09-04

**Authors:** Yan Wang, Kai-Ming Wang, Xin Zhang, Wenzhao Wang, Wei Qian, Fang-Fang Wang

**Affiliations:** 1 State Key Laboratory of Plant Genomics, Institute of Microbiology, Chinese Academy of Sciences, Beijing, China; 2 School of Life Science, Yunnan University, Kunming, Yunnan, China; 3 Shandong First Medical University & Shandong Academy of Medical Sciences, Jinan, Shandong, China; 4 National Key Laboratory of Intelligent Tracking and Forecasting for Infectious Diseases, National Institute for Communicable Disease Control and Prevention, Chinese Center for Disease Control and Prevention, Beijing, China; 5 State Key Laboratory of Mycology, Institute of Microbiology, Chinese Academy of Sciences; 6 CAS Center for Excellence in Biotic Interactions, University of Chinese Academy of Sciences, Beijing, China; University of Maryland, UNITED STATES OF AMERICA

## Abstract

The body temperature of Warm-blooded hosts impedes and informs responses of bacteria accustomed to cooler environments. The second messenger c-di-GMP modulates bacterial behavior in response to diverse, yet largely undiscovered, stimuli. A long-standing debate persists regarding whether a local or a global c-di-GMP pool plays a critical role. Our research on a *Stenotrophomonas maltophili*a strain thriving at around 28°C, showcases BtsD as a thermosensor, diguanylate cyclase, and effector. It detects 37°C and diminishes c-di-GMP synthesis, resulting in a responsive sequence: the periplasmic c-di-GMP level is decreased, the N-terminal region of BtsD disengages from c-di-GMP, activates the two-component signal transduction system BtsKR, and amplifies *sod1-3* transcription, thereby strengthening the bacterium′s pathogenicity and adaptation during infections in 37°C warm *Galleria mellonella* larvae. This revelation of a single-protein c-di-GMP module introduces unrecognized dimensions to the functional and structural paradigms of c-di-GMP modules and reshapes our understanding of bacterial adaptation and pathogenicity in hosts with a body temperature around 37°C. Furthermore, the discovery of a periplasmic c-di-GMP pool governing BtsD-BtsK interactions supports the critical role of a local c-di-GMP pool.

## Author summary

The body temperature of mammals, typically around 37°C, serves as an important host-derived signal for pathogens thriving at substantially lower temperatures. The ability to detect and respond to this host-derived temperature signal is essential for a bacterium′s life cycle shift from a free-living state to pathogenicity. The c-di-GMP signaling network is predominant in detecting signals and regulating bacterial behaviors, leaving many input signals and their sensing mechanisms remain elusive. The significance of our research lies in uncovering the mechanism by which a single-protein c-di-GMP module detects 37°C and enhances the virulence and adaptability of *Stenotrophomonas maltophilia* during infection, broadening our understanding of the role played by a compartmentalized c-di-GMP pool and our knowledge of the simplicity of c-di-GMP modules. This breakthrough deepens our insight into how this bacterium transforms between free-living state and pathogenicity at 37°C, and opens new avenues for developing treatments targeting this pathway.

## Introduction

The body temperature of mammals, around 37°C, serves as both a host-derived signal and a stressor for pathogens thriving at temperatures remarkably lower than 37°C [1–3]. It stimulates various adaptive and pathogenicity-related traits in bacteria, such as adhesion, entry, biofilm formation, evasion of the immune system, and toxin production [4]. Additionally, it imposes oxidant stress on pathogenic bacteria by increasing the levels of reactive oxygen species (ROS) inside the cell [5,6]. Studies have implied that adaptation to the mammalian body temperature plays a crucial role in transforming an environmental microbe into a pathogenic microbe for mammals [7–9]. Significant progress has been achieved in understanding the molecular mechanisms by which bacteria recognize the mammalian body temperature. Various types of receptors, including DNA, RNA, and protein receptors, have been identified [10,11]. DNA receptors primarily function as promoters, altering the supercoiling structures in response to temperature changes to regulate binding with RNA polymerases and subsequent gene transcription [12]. RNA receptors, mainly located in the 5′ untranslated regions (UTRs) of certain mRNAs, undergo conformational changes upon exposure to the mammalian body temperature, blocking ribosome binding sites (RBSs) and thus regulating protein production post-transcriptionally [13,14]. Protein receptors undergo polymeric or conformational change to regulate their activities, including transcriptional repressors [15], proteases [16], and receptor histidine kinases (RHKs) [17].

Cyclic diguanylate (c-di-GMP) signaling network plays a predominant role in granting bacteria the capacity to sense environmental conditions and subsequently adjust bacterial physiological and chemical processes accordingly [18]. c-di-GMP serves as a crucial second messenger within the cytoplasmic space of bacteria, binding to and regulating the activity of its effectors to control bacterial behaviors, including adhesion, biofilm formation, swimming, virulence, and stress responses [19]. Synthesized from two GTP molecules by diguanylate cyclases (DGCs) featuring a GGDEF domain, c-di-GMP is broken down into 5′-phosphoguanylyl-(3′,5′)-guanosine (pGpG) and eventually into GMP by phosphodiesterases (PDEs) containing either an HD-GYP or an EAL domain [19]. Considerable progress has been made in identifying the enzymes that metabolize c-di-GMP and its binding effectors. However, knowledge regarding the stimuli and sensing mechanisms responsible for regulating the expression or activity of c-di-GMP metabolizing enzymes remains limited [20–22]. Although the expression of these enzymes may react to stimuli, the rapid regulation of c-di-GMP levels facilitating just-in-time effector control could be achieved through direct regulation of DGCs and PDEs activities by stimuli, suggesting the priority of DGCs and PDEs as sensors. Surprisingly, nearly none advancements have been made in understanding the role of c-di-GMP signaling networks in temperature sensing, nor in identifying DGCs and PDEs as direct thermal sensors, except only one case reporting the DGC TdcA from *Pseudomonas aeruginosa* as a thermal sensor of the temperature at 37°C by Almblad *et al* [23]. TdcA was found to detect a temperature increase from 25°C to 37°C using the thermosensitive Per-Arnt-SIM (thermoPAS) domain to enhance its cyclase activity, leading to an elevation in cellular c-di-GMP levels. Subsequently, c-di-GMP binds to and regulates the activities of downstream effectors, such as FleQ, influencing the biofilm formation and motility of *Pseudomonas aeruginosa*. This marks the first instance where c-di-GMP signaling networks have been recognized for their ability to sense temperature through the sensor domain of a DGC.

*Stenotrophomonas maltophilia* is an emerging opportunistic bacterium capable of causing blood-stream infections and pneumonia, particularly in immunocompromised or immunosuppressed patients [24]. While not much progress has been made in understanding how this bacterium recognizes host-derived signals, an exception is the discovery of SisP detecting ferrous iron to increase its activity in degrading c-di-GMP [25]. SisP, which contains both a GGDEF and an EAL domain, utilizes its PAC-PAS-PAC-PAC domain to specifically bind with ferrous iron, leading to an enhancement in the EAL domain′s activity in degrading c-di-GMP. This results in a decrease in the c-di-GMP level and triggers the phosphorylation and activation of FsnR by releasing both RavS and FsnR from binding with c-di-GMP. Subsequently, FsnR functions as a transcriptional activator to positively regulate the expression of flagellar genes, promoting swimming motility crucial for bacterial dissemination and pathogenicity [26]. Despite c-di-GMP signaling networks being implicated in *S*. *maltophilia*′s temperature response [27], progress in identifying thermal sensors and understanding the mechanisms involved in temperature perception remains limited.

*S*. *maltophilia* CGMCC 1.1788 was originally isolated from a patient′s oropharyngeal region and shows a preference for thriving at approximately 28°C [25,26,28]. In this study, we unveil the identification of a c-di-GMP module that effectively modulates bacterial infectivity towards *Galleria mellonella* at 37°C, yet not at 28°C. Within this c-di-GMP module, the GGDEF domain of BtsD (body temperature-sensitive diguanylate cyclase) generates c-di-GMP, and orchestrates periplasmic c-di-GMP levels potentially through the cytosolic generation accompanying the periplasmic transportation, while its N-terminal region acts as an effector to bind c-di-GMP. During the infection process, BtsD detects 37°C to reduce c-di-GMP production, resulting in reduced levels of periplasmic c-di-GMP. Subsequently, the N-terminal region of BtsD disengages from c-di-GMP and thus binds with the sensor domain of BtsK (body temperature-sensitive kinase), leading to decreased phosphorylation levels of BtsK and then BtsR (body temperature-sensitive response regulator). BtsR subsequently upregulates the expression of the putative superoxide dismutase-encoding cluster (*sod1-3*). These genes play a critical role in bacterial pathogenicity and *in vivo* adaptation when *S*. *maltophilia* CGMCC 1.1788 infects *G*. *mellonella* at 37°C, but not at 28°C. Our research reveals that a singular protein-composed c-di-GMP module translates physical signals to biochemical ones, the periplasmic c-di-GMP level. This breakthrough adds new layers to our comprehension of the functional and structural paradigms of c-di-GMP modules and carves out fresh pathways for therapeutic developments. Given the periplasmic c-di-GMP level, a local c-di-GMP pool rather than a global one, orchestrates the activity of the BtsD-BtsK-BtsR system in modulating bacterial adaptability and pathogenicity, our findings support the critical roles played by local c-di-GMP pools and provides a novel mechanism to regulate local c-di-GMP levels.

## Results

### BtsD enhances pathogenicity and *in vivo* adaptability of *S*. *maltophilia* at 37°C

**Fig 1:**
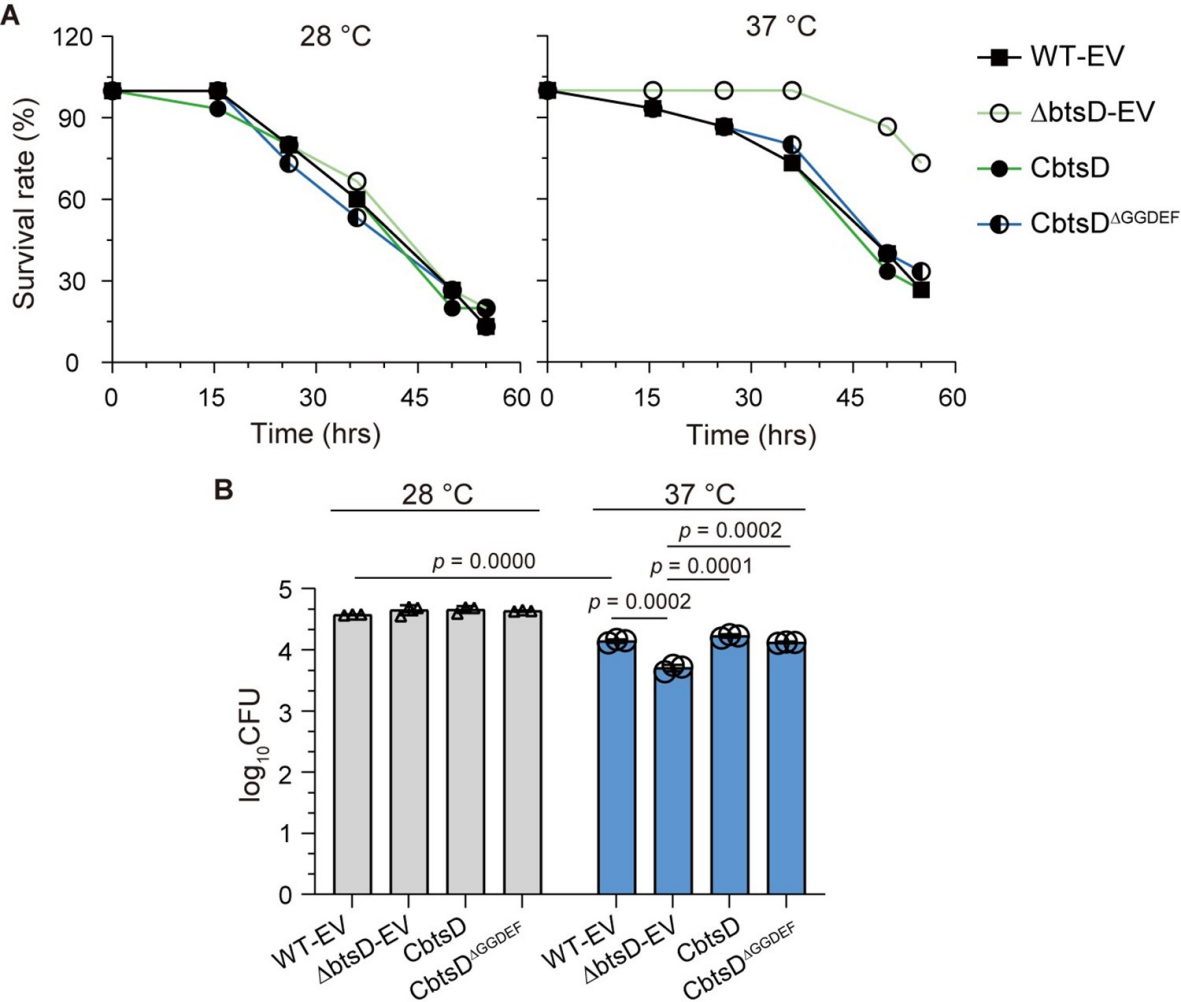
BtsD enhances pathogenicity and *in vivo* adaptability of *S*. *maltophilia* at 37°C. **(A)** *G*. *mellonella* larvae killing assay, demonstrating larval mortality following injection of specified bacterial cultures at 28°C and 37°C, respectively. The data averages are presented, with the original data dots and errors shown in S1A Fig. **(B)** Bacterial count assay, displaying the numbers of viable bacteria after injection and collection from *G*. *mellonella* larvae. The column and line charts depicted are representative of three independent experiments with comparable outcomes. Data in the bacterial count assay are presented as mean ± SD of three independent replicates. Statistical significance was evaluated using the two-tailed unpaired Student’s *t-*test. The *p* values of each of the specified two groups are presented upside. WT-EV: the wide-type strain bearing empty pBBR1MCS2 vectors; ΔbtsD-EV: the *btsD* deletion mutant bearing empty pBBR1MCS2 vectors; CbtsD: the complementary strain constitutively expressing *btsD* in *btsD* deletion background; CbtsD^ΔGGDEF^: the complementary strain constitutively expressing the recombinant *btsD*^*ΔGGDEF*^, with the GGDEF domain deleted, in *btsD* deletion background.

We developed an animal model using *Galleria mellonella* larvae, a widely used model organism with a preferred growth temperature range of 28–37°C [29,30], to screen for putative bacterial sensors and regulators involved in bacteria overcoming the temperature constraint to infect endothermic animals. By evaluating the mortality of *G*. *mellonella* larvae following bacterial culture injections and assessing the survival of the collected bacteria from *G*. *mellonella* larvae at 28°C and 37°C, we investigated the pathogenicity and *in vivo* adaptability of a bacterial strain during infection at 37°C. Our results showed that a mutation in *btsD* (ΔbtsD-EV, the strain with *btsD* deleted and bearing empty pBBR1MCS2 vectors) notably decreased larval mortality and bacterial survival at 37°C, whereas complementation of *btsD* (CbtsD strain, constitutively expressing *btsD* in *btsD* deletion background) restored the phenotype (Figs 1A, 1B, and S1A). Interestingly, *btsD* mutation and complementation did not significantly impact the outcomes at 28°C. We confirmed that the observed effects were not due to growth deficiencies by comparing growth curves of the three strains, WT-EV (the wild type strain bearing empty pBBR1MCS2 vectors), ΔbtsD-EV, and CbtsD (S2 Fig). These findings indicate the positive role of BtsD in regulating the pathogenicity and *in vivo* adaptability of *S*. *maltophilia* CGMCC 1.1788 during infection at 37°C, suggesting BtsD aids this bacterium overcoming the temperature constraint to infect endothermic animals.

Additionally, we noted a significant decrease in the survival of WT-EV at 37°C relative to 28°C (Figs 1B and S1A), underscoring the notion that the body temperature of mammalian hosts acts as a limiting factor for the infection by bacteria adapted to cooler environments. Furthermore, we noted that complementation of *btsD*^*ΔGGDEF*^ (CbtsD^ΔGGDEF^ strain, constitutively expressing *btsD*^*ΔGGDEF*^ in *btsD* deletion background, *btsD*^*ΔGGDEF*^: *btsD* with its GGDEF domain-encoding sequences, 836–1008 aa, deleted) successfully reversed the reduction in larval mortality and bacterial survival caused by *btsD* deletion at 37°C (Figs 1A, 1B, and S1A). This finding indicates that the remaining portion of BtsD, excluding the GGDEF domain, plays a positive role in *S*. *maltophilia*′s ability to overcome the temperature constraint to infect endothermic animals.

### BtsD is a thermosensor regulating c-di-GMP levels in the periplasm

**Fig 2:**
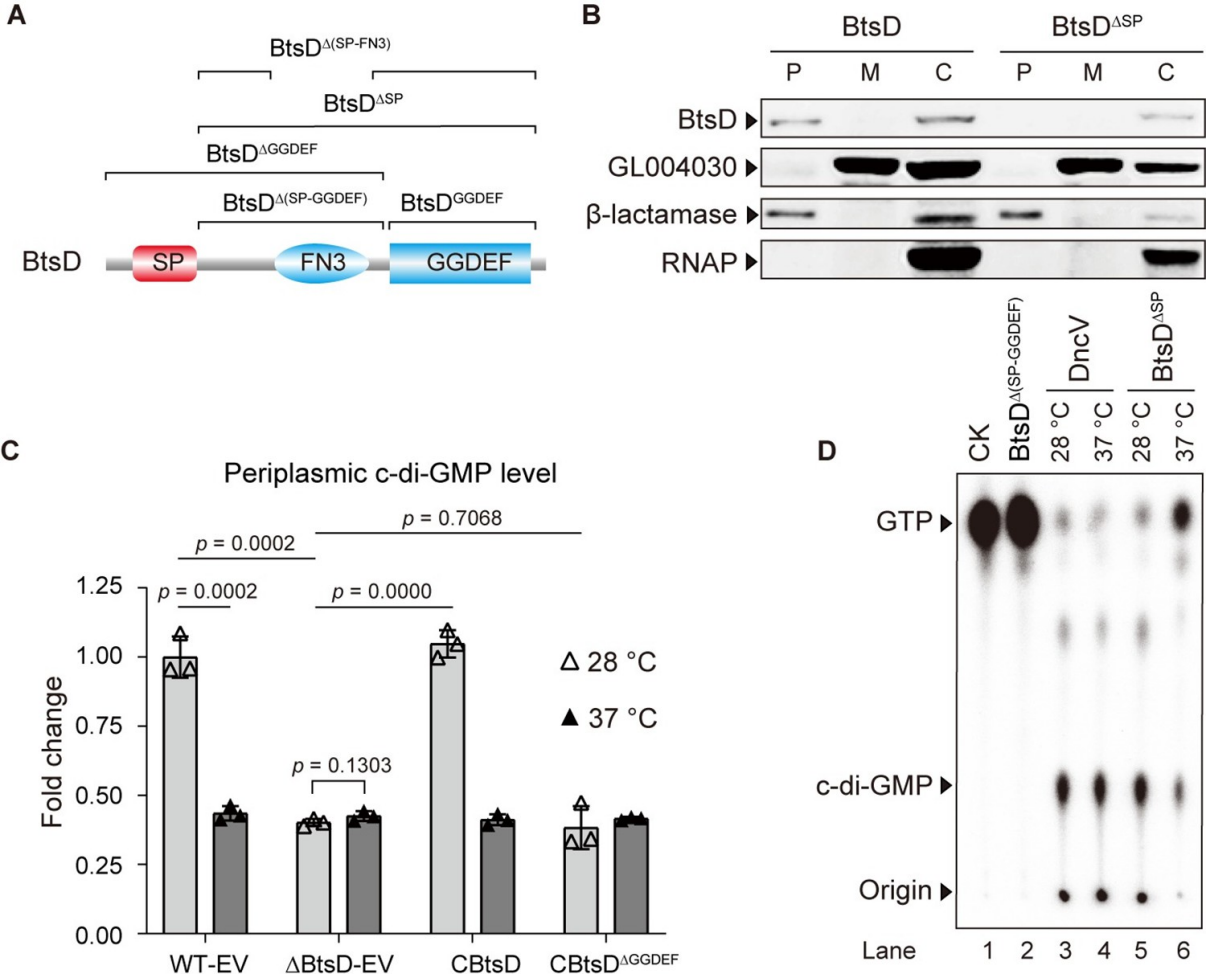
BtsD is a thermosensor regulating periplasmic levels of c-di-GMP. **(A)** The secondary structures of BtsD and its recombinant proteins. SP represents the signal peptide identified by SignalIP 4.0, FN3 represents the FN3 domain under the SMART accession number SM000060, and GGDEF represents the GGDEF domain under the accession number SM000267. The recombinant variants are as follows: BtsD^ΔSP^, with the SP deleted; BtsD^ΔGGDEF^, with the GGDEF domain deleted; BtsD^Δ(SP-GGDEF)^, with both the SP and GGDEF domain deleted; BtsD^Δ(SP-FN3)^, with both the SP and FN3 domain deleted; and BtsD^GGDEF^ containing the only GGDEF domain. **(B)** Western blot analyses revealed the presence of BtsD in the periplasm of the strain expressing His-tagged wild-type BtsD, as opposed to the strain expressing recombinant BtsD^ΔSP^ with the SP deleted. To exclude cross-contamination, the α subunit of RNA polymerase, GL004030, and β-lactamase were used as controls for cytosolic, membrane, and periplasmic proteins, respectively. BtsD: the strain expressing the His-tagged wild-type BtsD; BtsD^ΔSP^: the strain expressing the His-tagged BtsD^ΔSP^. **(C)** Periplasmic c-di-GMP levels quantified by LC-MS/MS. The isolated periplasmic fractions, verified to be free of contamination by Western blot analysis, were subjected to LC-MS/MS for c-di-GMP concentration measurement. The data are presented as mean ± SD of three independent replicates. Statistical significance was evaluated using the two-tailed unpaired Student’s *t-*test. The *p* values for each of the specified two groups are presented upside. Details of the strains are described above. **(D)** Reduced levels of c-di-GMP generated by purified BtsD^ΔSP^ at 37°C compared to 28°C. BtsD^Δ(SP-GGDEF)^ served as the negative control, and bands of DncV products were used to indicate the location of c-di-GMP separated by thin layer chromatography (TLC). DncV is a c-di-GMP synthetase from *Escherichia coli*. All presented data are representatives of three independent replicates, yielding consistent outcomes.

BtsD was initially predicted to be a periplasmic DGC based on the presence of a putative signal peptide (SP) at the N-terminal and a GGDEF domain at the C-terminal (Fig 2A), a prediction that was subsequently validated. Firstly, we meticulously isolated and collected components from the cytoplasmic, membrane, and periplasmic space for western blotting analyses. To ensure the absence of cross-contamination, the α-subunit of RNA polymerase served as the control for cytosolic proteins, β-lactamase for periplasmic proteins, and GL004030 for membrane proteins. BtsD bands were detected in both the cytosolic and periplasmic components in the strain containing full-length BtsD encoding sequences, but only in the cytosolic component when the SP sequences were deleted (BtsD^ΔSP^, Fig 2B). This observation indicates that BtsD is located in the periplasm of *S*. *maltophilia* CGMCC 1.1788. Additionally, retaining BtsD in the cytosol, via deleting the SP (CbtsD^ΔSP^, constitutively expressing *btsD*^*ΔSP*^ in *btsD* deletion background), remarkably decreased the bacterium′s swimming motility compared to the wild type strain (WT-EV) (S3 Fig). In contrast, neither the deletion of *btsD* (ΔbtsD-EV) nor its constitutive expression (CbtsD) significantly affected swimming motility (S3 Fig). This finding highlights the importance of BtsD′s signal peptide, thereby its periplasmic location, in ensuring its proper function in regulating bacterial behaviors. Given that changes in swimming motility are commonly associated with alterations in cellular c-di-GMP levels in bacteria [18], it is speculated that BtsD generates c-di-GMP *in vivo* and might modulate local c-di-GMP levels in its wild type form, as evidenced by the distinctive roles of *btsD* and *btsD*^*ΔSP*^ in regulating bacterial swimming motility (S3 Fig). Subsequently, we evaluated the synthetase activity of BtsD in producing c-di-GMP both *in vivo* and *in vitro*. We quantified periplasmic c-di-GMP levels in the specified strains by isolating and concentrating periplasmic components, verified to be free of contamination by cytosolic components via Western blot analysis. A notable reduction in periplasmic c-di-GMP levels was observed in both strains ΔbtsD-EV and CBtsD^ΔGGDEF^ (serving as the negative controls) compared to WT-EV and CbtsD strains at 28°C (Fig 2C). These findings suggest that BtsD is capable of producing c-di-GMP at 28°C, a conclusion further verified by the detection of c-di-GMP bands following the incubation of GTP with either the recombinant protein BtsD^ΔSP^ (the active form of BtsD with the SP sequences 1–42 aa deleted) or BtsD^GGDEF^ (comprising the GGDEF domain of BtsD, 836–1008 aa of BtsD), rather than BtsD^Δ(SP-GGDEF)^ (entailing BtsD with both its SP and GGDEF domain deleted) (Fig 2D). These results suggest that BtsD relies on its GGDEF domain to produce c-di-GMP at 28°C, and the c-di-GMP production by BtsD governs the periplasmic c-di-GMP level. Since no detectable GTP was observed in the isolated periplasmic components (S4 Fig), it is predicted that BtsD generates c-di-GMP in the cytoplasm, which is then transported to the periplasm.

Furthermore, a pronounced decrease was observed in both the periplasmic levels of c-di-GMP in WT-EV and CBtsD strains, as well as in c-di-GMP production following the incubation of GTP with BtsD^ΔSP^, at 37°C compared with that at 28°C (Fig 2C and 2D). These observations indicate that BtsD is directly responsive to the temperature of 37°C, causing a decrease in its synthetase activity. Additionally, polymeric structure analysis using native gels showed a significant decrease in the tetrameric forms of BtsD at 37°C compared to 28°C, when the reaction systems either directly incubated at the specified temperatures or subjected to a gradual 30-min temperature upshift (S5A Fig). This suggests that the tetramers are the active form of BtsD in generating c-di-GMP. Additionally, it was found that the solo GGDEF domain of BtsD directly alters its synthetase activity in response to temperature changes from 28°C to 37°C. This is evidenced by a significant decrease in c-di-GMP generation by BtsD^GGDEF^ (the recombinant BtsD protein containing only its GGDEF domain, 836–1008 aa) when the reaction was set at 37°C compared to 28°C (S5B Fig). Furthermore, replacing the GGDEF domain with that of WspR, a DGC from *Pseudomonas aeruginosa* that is active at 37°C [23], renders BtsD incapable of generating c-di-GMP (S5C Fig). The findings demonstrate the irreplaceability and the critical role played by the GGDEF domain in the temperature perception of BtsD, as well as a potential interaction between BtsD′s N-terminal region and GGDEF domain. The molecular basis underlying the significance of the GGDEF domain of BtsD might associate with two variations, Arg^895^ and Ser^897^, revealed by sequence alignment analysis conducted among the GGDEF domains of BtsD and several DGCs active at 37°C (S6 Fig).

In summary, BtsD functions as a sensor for the mammalian body temperature, directly sensing the temperature increase from 28°C to 37°C, with its GGDEF domain playing a critical role. This temperature increase leads to a reduction in the tetramers of BtsD, thereby decreasing its synthetase activity and the level of periplasmic c-di-GMP. However, the molecular basis behind the process remains to be unraveled through dynamic analysis on BtsD′s conformational changes upon temperature variations. This is for the first time we have localized c-di-GMP in the periplasm, expanding our understanding of the regulatory role of c-di-GMP signaling network from the cytoplasmic to the periplasmic space of bacteria. Additionally, the irreplaceability of the GGDEF in BtsD and its automatic modulation of c-di-GMP generation upon temperature changes merit further study.

Together with the discovery that the restoration of indicated bacterial traits was observed through the complementation of *btsD*^*ΔGGDEF*^ in a *btsD* deletion background at 37°C (Fig 1A and 1B), our research indicates that the GGDEF domain of BtsD may suppress its N-terminal region, thus disabling *S*. *maltophilia* in overcoming the temperature constraint to infect endothermic animals. This suppression could potentially be alleviated through conformational alterations in the GGDEF domain of BtsD or a following reduction in its synthetase activity and the periplasmic c-di-GMP levels upon 37°C induction.

### BtsD triggers the TCS BtsKR to enhance the pathogenicity and *in vivo* adaptability of *S*. *maltophilia* at 37°C

**Fig 3:**
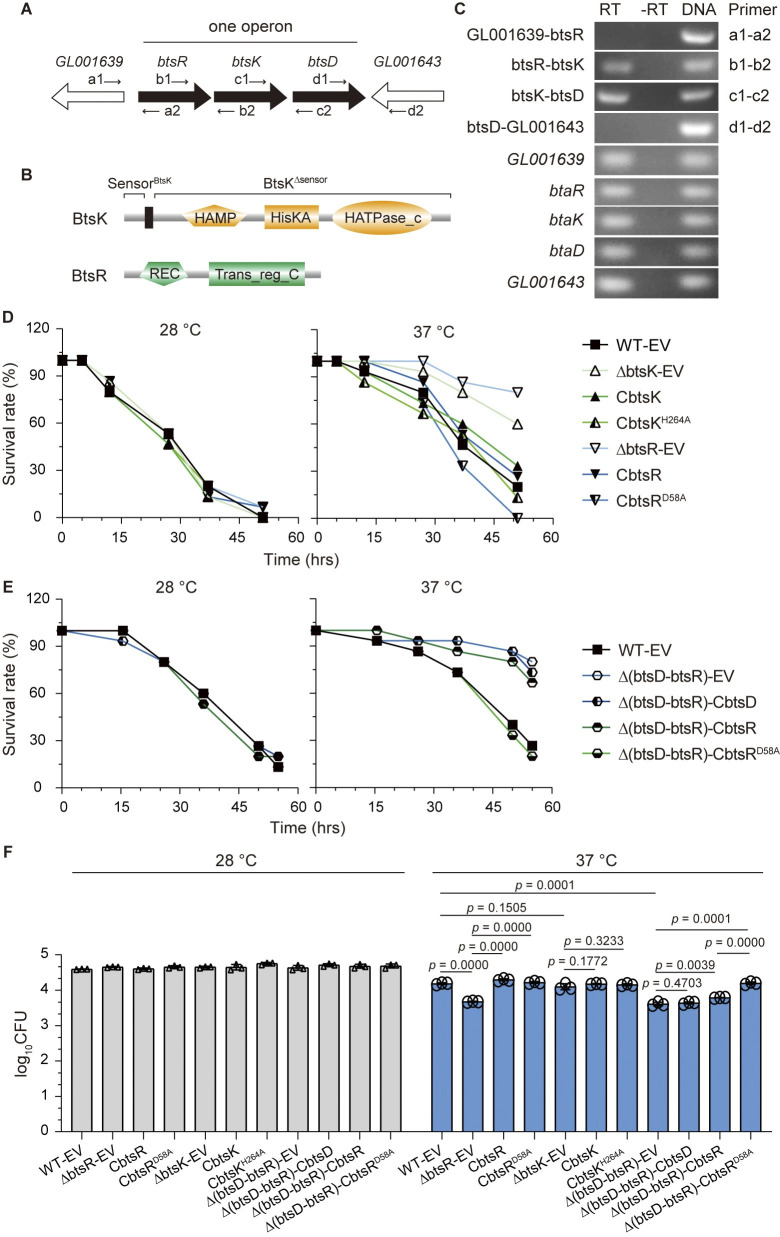
BtsD triggers the BtsKR to enhance the pathogenicity and *in vivo* adaptability at 37°C. **(A)** Locations of *btsD*, *btsK*, and *btsR* in the genome of *S*. *maltophilia* CGMCC 1.1788 and primers used to investigate the operon structure. **(B)** Secondary structures of both BtsK and BtsR predicted by SMART with the vertical bar indicating the transmembrane region. HAMP, HisKA, HAPase_c, REC, and Trans_reg_C domains were indicated. Sensor^BtsK^ denotes the recombinant BtsK containing only the sensor domain, while BtsK^Δsensor^ refers to the recombinant BtsD with its sensor domain deleted. **(C)** Validation of the operon *btsR-btsK-btsD* by RT-PCR. RT represents PCR using cDNA reversely transcribed from total RNAs as templates. While -RT served as the negative control using total RNAs without reverse transcription, and DNA as the positive control with genomic DNA as templates. **(D)** and **(E)** *G*. *mellonella* larvae killing assay, demonstrating larval mortality following injection of specified bacterial cultures at 28°C and 37°C, respectively. For **(D)** and **(E)**, the data averages are presented, with the original data dots and errors shown in S1B and S1C Fig, respectively. **(F)** Bacterial count assay, displaying the numbers of viable bacteria after injection and collection from *G*. *mellonella* larvae. Data in **(F)** are presented as mean ± SD of three independent replicates. Statistical significance was evaluated using the two-tailed unpaired Student’s *t-*test, with *p* < 0.05 indicating a statistically significant difference. all the shown data are representative of three independent experiments with comparable outcomes. WT-EV is described above. ΔbtsK-EV and ΔbtsR-EV: the *btsK* and *btsR* deletion mutant carrying empty pBBR1MCS2 vectors; CbtsK and CbtsR: the complementary strains constitutively expressing *btsK* and *btsR* in *btsK* and *btsR* deletion background, respectively; CbtsK^H264A^ and CbtsR^D58A^: the complementary strains constitutively expressing the recombinant *btsK* with its His^264^-encoding sequences replaced by an Ala-encoding sequences and the recombinant *btsR* with its Asp^58^-encoding sequences replaced by an Ala-encoding sequences; Δ(btsD-btsR)-EV: the mutant with both *btsD* and *btsR* deleted, carrying empty pBBR1MCS2 vectors; Δ(btsD-btsR)-CbtsD, Δ(btsD-btsR)-CbtsR, and Δ(btsD-btsR)-CbtsR^D58A^: the complementary strains constitutively expressing *btsD*, *btsR*, and *btsR*^*D58A*^ in a background where both *btsD* and *btsR* are deleted, respectively.

*btsD* was predicted to be in an operon with *btsK* and *btsR* in *S*. *maltophilia* CGMCC 1.1788 (Fig 3A). BtsK encodes a putative RHK with a periplasmic sensor domain and a conserved phosphorylation site His^264^ within the HisKA domain, while BtsR is a potential response regulator (RR) with a conserved phosphorylation site Asp^58^ within the REC domain (Fig 3B). These proteins might constitute a two-component signal transduction system (TCS), one of the predominant signaling systems responsible for signal detection and response regulation in bacteria [31]. This raises the speculation that the potential TCS BtsKR cooperates with BtsD in regulating bacterial pathogenicity and *in vivo* adaptability at 37°C based on the common notion that prokaryotes often cluster genes with similar or related functions in the same operon [32]. These speculations were confirmed through investigation. Initially, the operon structure of *btsD* was examined using reverse transcription polymerase chain reactions (RT-PCR). Bands of the products were detected using b1-b2 or c1-c2 primers, rather than a1-a2 and d1-d2, with the cDNAs reverse-transcribed from RNAs extracted from the wild-type strain as the template (Fig 3C). These findings suggest that *btsR*, *btsK*, and *btsD* are transcribed together as part of an operon. Subsequently, the roles of both BtsK and BtsR in regulating bacterial pathogenicity and *in vivo* adaptability were confirmed. Deletion of *btsR* (ΔbtsR-EV, the strain with *btsR* deleted and bearing empty pBBR1MCS2 vectors) significantly decreased larval mortality and bacterial survival at 37°C, but not at 28°C. This deficiency was fully restored by complementation with either the wild-type gene (CbtsR, constitutively expressing *btsR* in *btsR* deletion background) or a gene encoding a recombinant protein mimicking the unphosphorylated state of the wild-type protein (CbtsR^D58A^, constitutively expressing *btsR*^*D58A*^, with Asp^58^ replaced by Ala.) (Figs 3D, 3F and S1B). Similarly, deletion of *btsK* (ΔbtsK-EV, the strain with *btsK* deleted and bearing empty pBBR1MCS2 vectors) decreased larval mortality and bacterial survival with complementation using *btsK*^*H264A*^ (CbtsK^H264A^, constitutively expressing *btsK*^*H264A*^, with His^264^ replaced by Ala) being more effective than *btsK* (Figs 3D, 3F and S1B). These results suggest the positive contributions of BtsK and BtsR in regulating bacterial pathogenicity and *in vivo* adaptability, as well as a potential dephosphorylation of these proteins upon induction by 37°C.

Then we performed epistasis analyses to investigate the relationship between BtsD and the putative TCS BtsKR in regulating bacterial pathogenicity and *in vivo* adaptability. Considering that the RR is responsible for the TCS output, we generated Δ(btsD-btsR)-EV strains (deletion of both genomic *btsD* and *btsR* genes, carrying empty pBBR1MCS2 vectors) and the corresponding complementation strains: Δ(btsD-btsR)-CbtsD (complementation of BtsD), Δ(btsD-btsR)-CbtsR (complementation of BtsR), and Δ(btsD-btsR)-CbtsR^D58A^ (complementation of BtsR^D58A^) for the epistasis analyses. Our findings revealed a notable decrease in larvae mortality and bacterial survival rates at 37°C in the Δ(btsD-btsR)-EV strain compared to the wild type strain (WT-EV). Interestingly, complementation of BtsD and BtsR, but not BtsR^D58A^ which mimics the dephosphorylated state of BtsR, failed to rescue this deficiency (Figs 3E, 3F, and S1C). These results highlight a signaling pathway involving BtsD, BtsK, and BtsR that senses 37°C to regulate the pathogenicity and *in vivo* adaptability of *S*. *maltophilia*, with BtsD upstream of BtsKR potentially activating them by reducing their phosphorylation levels.

Sequence alignment analysis of the BtsD-BtsK-BtsR system against National Center for Biotechnology Information (NCBI) databases was performed. It was found that this system is conserved in most Gram-negative bacteria, including most species of *Stenotrophomonas*, *P*. *aeruginosa*, *Vibrio cholerae*, *Xanthomonas campestris*, and *Lysobacter auxotrophicus*. However, only a limited number of *Stenotrophomonas* species, including *S*. *sepilia*, *S*. *pavanii*, *S*. *geniculata*, and *S*. *cyclobalanopsidis*, have their BtsD homologs conserved in the SP (S7A Fig). Many other species within *Stenotrophomonas* genus, such as *S*. *lactitubi*, *S*. *bentonitica*, *S*. *rhizophila*, and *S*. *acidaminiphila*, exhibit varied SPs (S7A Fig). Interestingly, only two bacterial species outside the *Stenotrophomonas* genus, *P*. *aeruginosa* and *P*. *hibiscicola*, also have conserved signal peptides (S7A Fig). The results suggest that the SP of BtsD evolves faster than its other domains, implying distinct cellular locations and roles for its homologs, as well as varied functions of the BtsD-BtsK-BtsR system in bacteria. The phylogenic analysis based on the sequences of the signal peptides reveals more closer relationships between BtsD and its homologs in *S*. *sepilia*, *P*. *aeruginosa*, *S*. *pavanii*, *S*. *geniculate*, and *P*. *hibiscicola* (S7B Fig). The results may indicate horizontal gene transfer between bacterial species or gene loss in some species, demonstrating distinct selective pressures and adaptative strategies during bacterial evolution.

### The BtsD-BtsK-BtsR signaling pathway upregulates *sod1-3* transcription, enhancing *S*. *maltophilia* pathogenicity and *in vivo* adaptability at 37°C

Next, we performed RNA sequencing (RNA-seq) analyses to screen for putative target genes that are regulated by the BtsD-BtsK-BtsR signaling pathway, crucial for the pathogenicity and *in vivo* adaptability of *S*. *maltophilia* at 37°C. A total of 257 genes were screened based on the criteria: maintaining stable transcript levels in one strain (WT or ΔbtsD) while significantly changing in the other strain with a temperature shift from 28°C to 37°C, and exhibiting significant differences in transcript levels between strains at 37°C (S1 Table). These genes were annotated using the Gene Ontology (GO) and Kyoto Encyclopedia of Genes and Genomes (KEGG) databases to gain insight into their functions and roles in bacterial cells. While all 257 genes received GO annotations and functional classifications, only 65 genes were also annotated in KEGG (S8A and S8B Fig, and S2 Table). The majority of GO-annotated genes were categorized into five main GO secondary-level groups, including catalytic activity, cellular anatomical entity, binding, cellular process, and metabolic process. The catalytic activity and metabolic process groups primarily encompassed genes encoding a variety of enzymes (such as synthases, NTPases, kinases, transferases, proteases, reductases, and dismutases) involved in protein and small molecule metabolism and modification (e.g., NTPs and amino acids). The cellular anatomical entity, binding, and cellular process groups predominantly contained genes encoding assembly proteins, as well as components of efflux and transport systems. Approximately forty percent of the KEGG-annotated genes were allocated to four KEGG secondary-category pathways: signal transduction, amino acid metabolism, energy metabolism, and carbohydrate metabolism—with the latter three pathways closely linked to cellular metabolism. In summary, BtsD regulates a specific set of genes involved in diverse cellular processes, such as energy and substance metabolism, as well as the exchange of substances within and outside the cell at 37°C.

**Fig 4:**
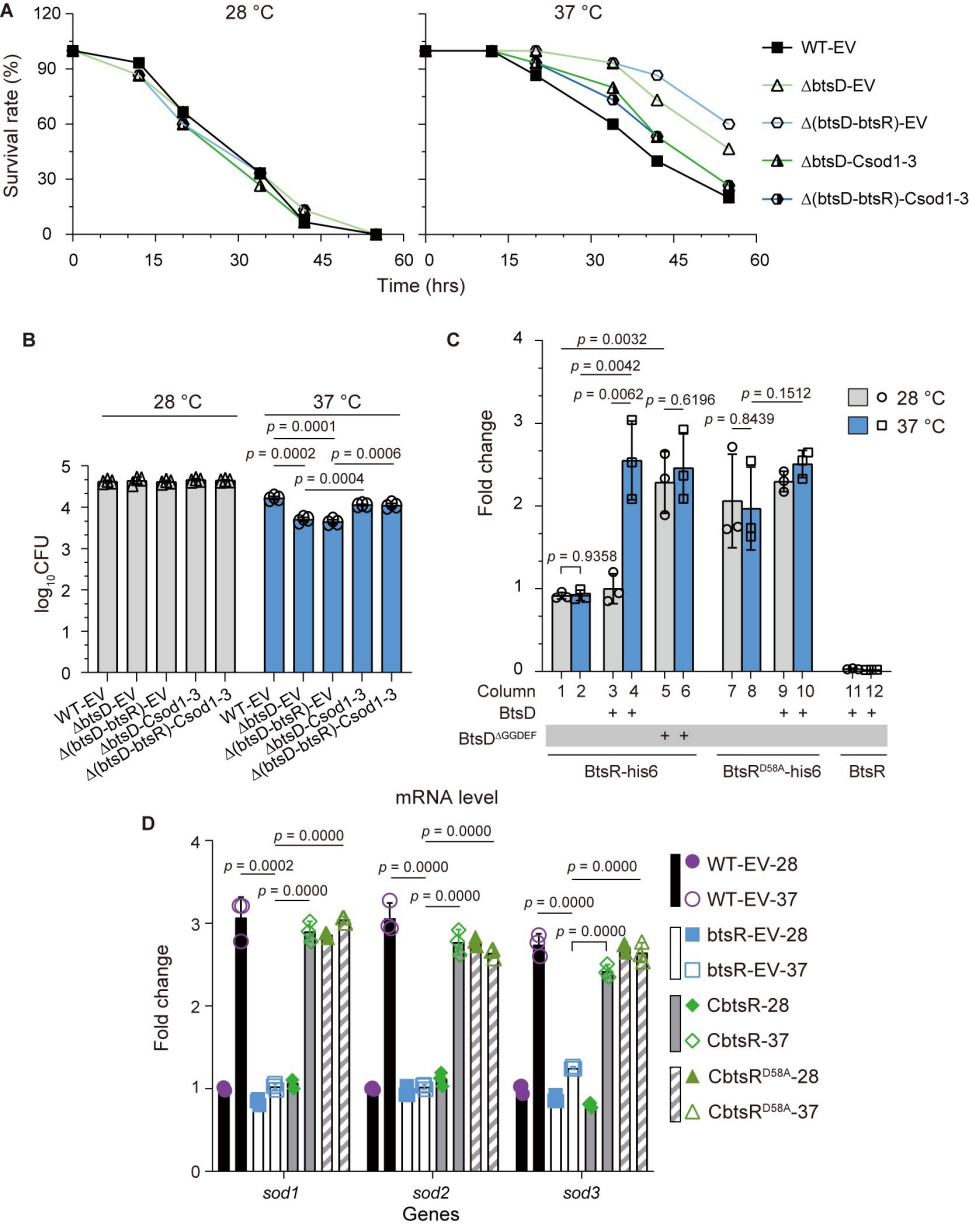
The BtsD-BtsK-BtsR pathway upregulates *sod1-3* transcription, enhancing pathogenicity and *in vivo* adaptability at 37°C. **(A)** *G*. *mellonella* larvae killing assay, demonstrating larval mortality following injection of specified bacterial cultures at 28°C and 37°C, respectively. The data averages are presented, with the original data dots and errors shown in S1D Fig. **(B)** Bacterial count assay, displaying the numbers of viable bacteria after injection and collection from *G*. *mellonella* larvae. **(C)** The ChIP-qPCR assay quantifying binding of His-tagged BtsR or BtsR^D58A^ with the *sod1-3* promoter in the presence of BtsD or its recombinant form BtsD^ΔGGDEF^, or in its absence with the non-tagged BtsR as the negative control. The presence or recombination of both BtsD and BtsR in the used strains is denoted below the panel. The recombination of BtsR includes BtsR-his6, the His-tagged BtsR, BtsR^D58A^-his6, the His-tagged BtsR^D58A^, and BtsR, the wild type BtsR. BtsD and BtsD^ΔGGDEF^, as described above, are denoted by a + sign to indicate their presence. **(D)** The transcript levels of *sod1-3* quantified by qRT-PCR in the presence or absence of BtsR at 28°C and 37°C. -28 and -37 represent the temperatures used to culture the specified strains. The shown data are representative of three independent experiments with comparable outcomes. Data are presented as mean ± SD of three independent replicates. Statistical significance was evaluated using the two-tailed unpaired Student’s *t-*test. The *p* values for each of the specified two groups are presented upside. ΔbtsD-Csod1-3 and Δ(btsD-btsR)-Csod1-3: the strains constitutively expressing the sod1-3 cluster in the backgrounds where *btsD* or both *btsD* and *btsR* are deleted. The other strains are described above.

Within the putative target genes, we observed that BtsD positively regulates the expression of a gene cluster consisting of three genes: *GL002776* (*sod1*), *GL002777* (*sod2*), and *GL002778* (*sod3*) (S1 Table). Among the proteins encoded by the cluster, Sod1 is identified as a superoxide dismutase, Sod2 as an oxidoreductase, and Sod3 remains uncharacterized (S2 Table). Given the elevated reactive oxygen species (ROS) levels in infected hosts and in bacteria undergoing a temperature upshift [33,34], we hypothesized that *sod1-3* are targets regulated by the BtsD-BtsK-BtsR pathway. This regulation may enhance bacterial pathogenicity and *in vivo* adaptability at 37°C by shielding against ROS-induced damage through the production of proteins that counteract ROS. To test this hypothesis, we initially investigated whether the expression of these three genes could rescue deficiencies in strains lacking either *btsD* or both *btsD* and *btsR*. Our results showed significant enhancements in bacterial pathogenicity and *in vivo* adaptability when *sod1-3* were expressed, as evidenced by increased larvae mortality and bacterial survival following the injection of strains ΔbtsD-Csod1-3 and Δ(btsD-btsR)-Csod1-3 compared to control strains ΔbtsD-EV and Δ(btsD-btsR)-EV (Figs 4A, 4B, and S1D). This indicates that the *sod1-3* gene cluster operates downstream of the BtsD-BtsK-BtsR pathway, playing a positive role in regulating bacterial pathogenicity and *in vivo* adaptability at 37°C.

Next, we explored the molecular mechanisms behind the regulation of the *sod1-3* expression by the BtsD-BtsK-BtsR pathway. Given that BtsR is a potential transcriptional factor with Trans_reg_C capable of binding to the promoter and initiating gene transcription (Fig 3B), we hypothesized that BtsR directly interacts with the *sod1-3* promoter to control its transcription. This hypothesis was validated using chromatin immunoprecipitation coupled with quantitative PCR (ChIP-qPCR). A notable increase in the amount of the promoter co-immunoprecipitated by anti-His monoclonal antibodies was observed in the strain expressing both BtsR-his6 and BtsD following a temperature change from 28°C to 37°C (Fig 4C, columns 3–4), indicating a direct binding interaction between BtsR and the *sod1-3* promoter, particularly at 37°C. This increase was not observed in the absence of BtsD (Fig 4C, columns 1–2), indicating that BtsD regulates the interaction between BtsR and the *sod1-3* promoter. Furthermore, the increased binding was sustained when BtsD was replaced by BtsD^ΔGGDEF^ or BtsR-His was substituted by BtsR^D58A^-His even in the absence of BtsD (Fig 4C, columns 5–10). These results align with the data presented in Figs 1 and 3, demonstrating that BtsD^ΔGGDEF^ and BtsR^D58A^ mimic the constitutively active forms of their respective proteins, suggesting the removal of the suppressive effect of the GGDEF domain on the N-terminal region of BtsD and the dephosphorylation of BtsR at 37°C. Furthermore, we conducted real-time quantitative polymerase chain reaction (qRT-PCR) and observed a significant decrease in *sod1-3* transcripts upon *btsR* deletion (strain ΔbtsR-EV) with a recovery observed upon the complementation of *btsR* (strain CbtsR), as well as *btsR*^*D58A*^ (CbtsR^D58A^) (Fig 4D). These findings suggest a positive role of BtsR and demonstrate the effectiveness of BtsR^D58A^ in mimicking the activated form of BtsR in regulating *sod1-3* expression. Therefore, the BtsD-BtsK-BtsR pathway directly regulates *sod1-3* transcription via the transcriptional activator BtsR capable of binding to the *sod1-3* promoter. Moreover, the regulation is activated by the dephosphorylation of BtsR following the stimulation of BtsD by 37°C.

In summary, the BtsD-BtsK-BtsR pathway enhances *sod1-3* expression through a series of cascade reactions: BtsD senses the temperature of 37°C, leading to conformational changes that reduce its synthetase activity. This in turn triggers self-activation by relieving the suppressive effect of the GGDEF domain on its N-terminal region. Subsequently, BtsD, via its N-terminal region, triggers the dephosphorylation of BtsR by putatively interacting with BtsK, which may reduce the autokinase activity of BtsK involved in phosphorylating BtsR through a common phosphotransfer mechanism between TCS components. BtsR, as a transcriptional activator, then stimulates the transcription of the *sod1-3* cluster by directly binding to the *sod1-3* promoter. The upregulation of *sod1-3* transcription significantly enhances the pathogenicity and *in vivo* adaptability of *S*. *maltophilia*, although it may necessitate modulation of the expression of other genes as well.

### BtsD releases its N-terminal region from c-di-GMP binding at 37°C and repositions it to bind with BtsK, reducing BtsK′s autokinase activity and decreasing phosphorylation levels of BtsKR

**Fig 5:**
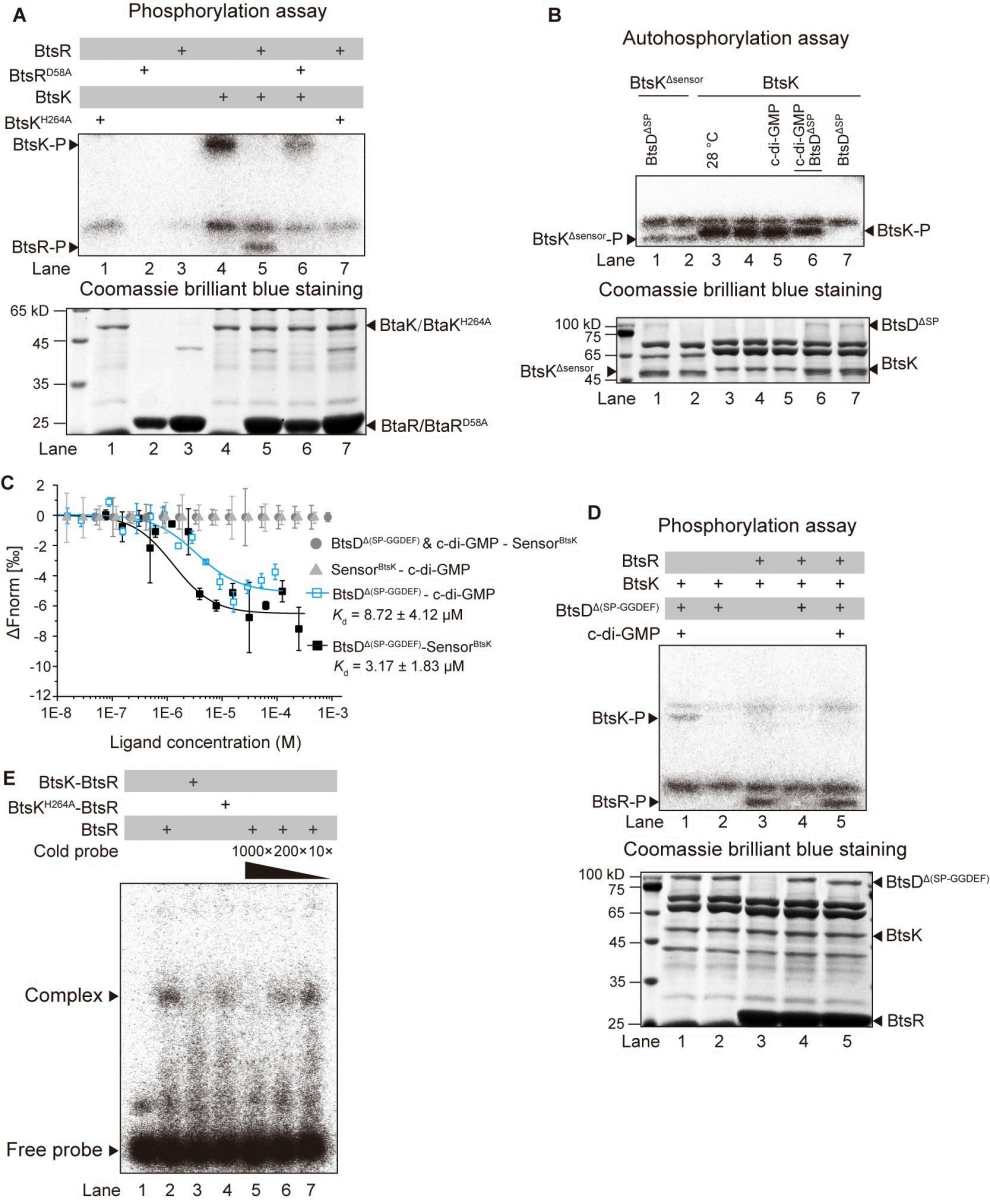
BtsD reduces BtsKR phosphorylation by enabling its N-terminal region to bind with BtsK′s sensor domain. **(A)** BtsK and BtsR constitutes a TCS. The upper panel shows phosphorylation bands of BtsK and BtsR. BtsK was incubated with excessive ATP to evaluate its autokinase activity, while BtsR was incubated with phosphorylated BtsK to access the phosphotransfer from BtsK to BtsR. BtsK^H264A^ and BtsR^D58A^ served as negative controls. The lower panel presents a Coomassie brilliant blue-stained gel indicating the protein amounts used in the assay. **(B)** BtsD^ΔSP^ inhibited the autokinase activity of BtsK by interacting with its sensor domain, which was reversed by preincubating BtsD^ΔSP^ with c-di-GMP. Preincubation of BtsK with BtsD^ΔSP^ or the preincubated BtsD^ΔSP^ and c-di-GMP mixture was conducted before the autophosphorylation assay. All reactions were conducted at 37°C, except for one reaction specified to be 28°C. **(C)** The N-terminal region of BtsD (BtsD^Δ(SP-GGDEF)^) bound with both c-di-GMP and the sensor domain of BtsK (Sensor^BtsK^), and preincubation of BtsD^Δ(SP-GGDEF)^ with c-di-GMP prevented the binding of BtsD^Δ(SP-GGDEF)^ with Sensor^BtsK^. MST assays were used the check binding affinities between indicated molecules. BtsD^Δ(SP-GGDEF)^ & c-di-GMP represents a preincubated mixture of BtsD^Δ(SP-GGDEF)^ and c-di-GMP. Data are presented as mean ± SD of three independent replicates. **(D)** The N-terminal region of BtsD reduced phosphorylation levels of both BtsK and BtsR, which was reversed by preincubating it with c-di-GMP. Preincubation of BtsD^Δ(SP-GGDEF)^ or the preincubated mixture of BtsD^Δ(SP-GGDEF)^ and c-di-GMP with BtsK or both BtsK and BtsR was done before initiating phosphorylation assays. **(E)** Unphosphorylated BtsR showed higher binding affinity with the *sod1-3* promoter than phosphorylated BtsR. BtsR was incubated with labeled *sod1-3* promoter probes with or without the competition of unlabeled probes (cold probes) to access its binding with the *sod1-3* promoter. Relative folds of the cold probes to the labeled probes were indicated. Preincubating BtsR with BtsK was done to obtain phosphorylated BtsR, and BtsK^H264A^ served as a negative control for preincubation with BtsR. All the shown data are representative of three independent experiments with comparable outcomes. All the proteins are described above. -P denotes the phosphorylation bands of the specified proteins.

Next, we conducted experiments to unravel the biochemical mechanisms involved in alleviating the GGDEF domain′s suppressive effect on the N-terminal region within the protein BtsD, and the reduced phosphorylation of BtsR activated by BtsD at 37°C. Initially, we confirmed that BtsK and BtsR constitute a TCS by observing bands of phosphorylated BtsK proteins upon incubation of BtsK, rather than BtsK^H264A^, with [γ-^32^P]-ATP, and bands of phosphorylated BtsR after a forty-five seconds-incubation of BtsR, but not BtsR^D58A^, with pre-phosphorylated BtsK (Fig 5A). These results indicate that BtsD may lower the phosphorylation of BtsR by inhibiting BtsK′s autokinase activity, consequently reducing phosphotransfer between BtsK and BtsR. Moreover, consistent intensity of phosphorylated BtsK bands at 28°C or 37°C, with or without c-di-GMP (Fig 5B, lanes 3–5), suggests a protein-protein interaction between BtsD and BtsK, rather than a response to c-di-GMP or temperature changes altering BtsK′s autokinase activity.

Therefore, we incubated BtsK with BtsD^ΔSP^ in the reaction system and found a significant decrease in BtsK′s phosphorylation level in the absence of c-di-GMP, but a recovery in the presence of c-di-GMP (Fig 5B, lanes 4, 6, and 7). Moreover, we detected no remarkable decrease when BtsK was replaced by BtsK^Δsensor^ (the recombinant protein with 35–468 aa of BtsK) (Fig 5B, lanes 1–2). These observations indicate BtsK might directly bind with BtsD through its periplasmic sensor domain, as well as a hypothesized manner used by BtsD to remove the suppressive effect of the GGDEF domain on its N-terminal region: the GGDEF domain reduces its synthetase activity at 37°C, resulting a decrease in the c-di-GMP level, which frees the N-terminal region from binding with c-di-GMP and enables it to bind with the sensor domain of BtsK. This hypothesis was validated by the MicroScale Thermophoresis (MST) analyses results, showing that BtsD^Δ(SP-GGDEF)^ directly bound with c-di-GMP and Sensor^BtsK^, and that pre-incubation of BtsD^Δ(SP-GGDEF)^ with c-di-GMP prevented BtsD^Δ(SP-GGDEF)^ from binding with Sensor^BtsK^ (Fig 5C). Furthermore, it is supported by subsequent molecular docking analyses results, showing two putative hydron bonds between c-di-GMP and the N-terminal region of BtsD (S9A and S9B Fig). Between the two hydron bond-forming sites, Asn^133^ is situated within a reported c-di-GMP binding motif, RXXD [35]. Subsequently, we tested the role of BtsD^Δ(SP-GGDEF)^ in regulating phosphorylation levels of BtsK and its paired BtsR. We observed no recognizable phosphorylated BtsK or BtsR bands in the presence of BtsD^Δ(SP-GGDEF)^ unless BtsD^Δ(SP-GGDEF)^ had been preincubated with c-di-GMP (Fig 5D). Therefore, BtsD utilizes its N-terminal region to bind with the periplasmic sensor domain of BtsK, leading to a reduction in BtsK′s autokinase activity, resulting in low levels of phosphorylated BtsK and consequently BtsR. Additionally, besides the potential involvement in BtsD′s N-terminal region in interacting with BtsK′s sensor domain through a possible direct c-di-GMP-binding mechanism, the FN3 domain is also essential for regulating its DGC activity. This is indicated by the significant reduction in c-di-GMP synthesis in BtsD^Δ(SP-FN3)^ (the recombinant BtsD with the SP and FN3 domain deleted) compared to BtsD^ΔSP^ (S9C Fig). The exact roles of the FN3 domain of BtsD merit further study.

## Discussion

**Fig 6:**
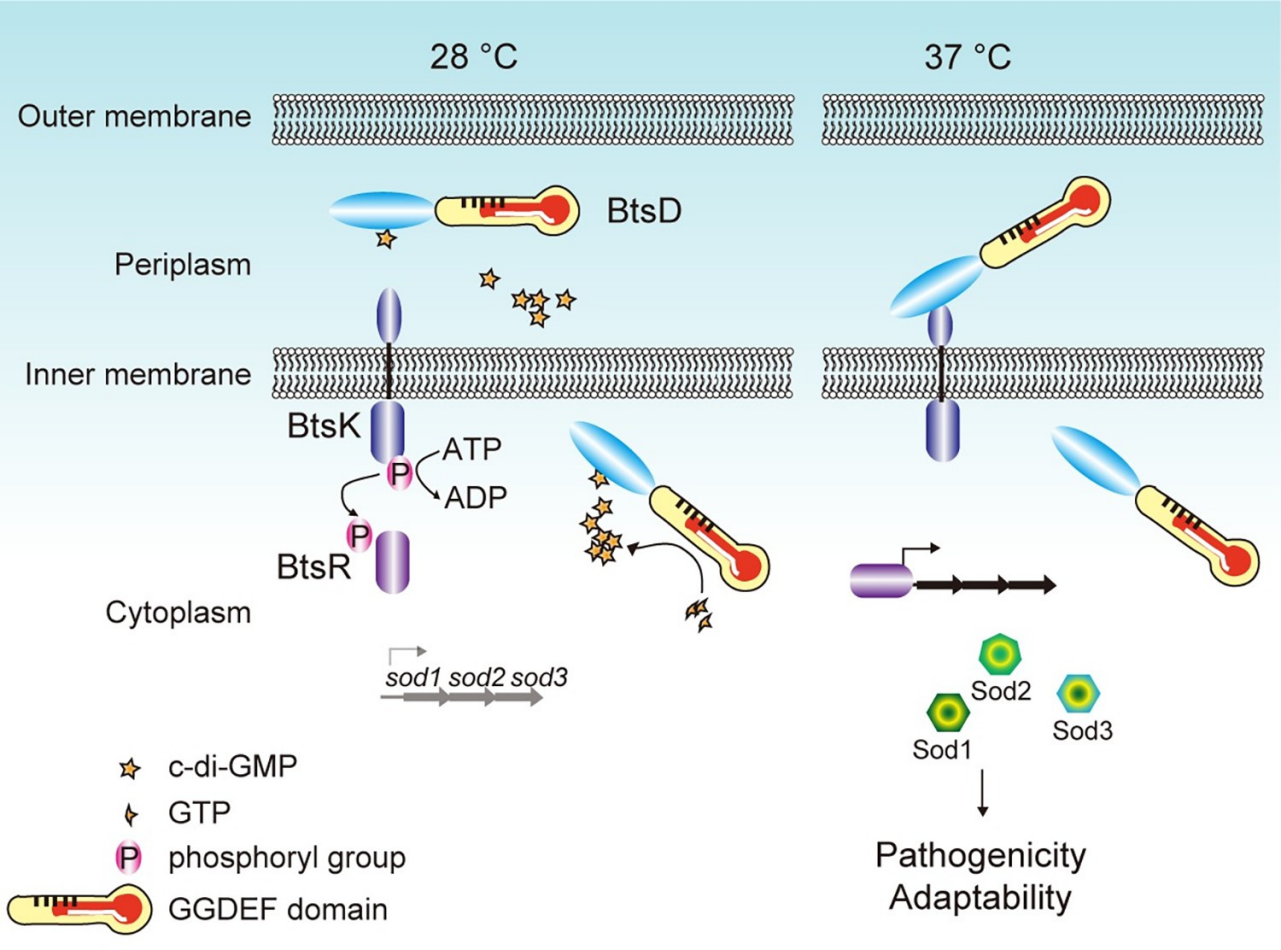
The BtsD-BtsK-BtsR system detects 37°C to regulate *S*. *maltophilia*′s pathogenicity and *in vivo* adaptability. BtsD acts as a thermosensor, detecting 37°C to decrease its c-di-GMP synthetase activity, reducing the periplasmic c-di-GMP level and thereby releasing its N-terminal region from binding with c-di-GMP. The release allows the N-terminal region to interact with the sensor domain of BtsK, causing the inhibition of BtsK′s autokinase activity, leading to decreased phosphorylation levels of both BtsK and its paired BtsR. Consequently, BtsR binds to the *sod1-3* promoter, enhancing *sod1-3* transcription and thereby boosting the pathogenicity and *in vivo* adaptability of *S*. *maltophilia* during its invasion of *G*. *mellonella* larvae at 37°C.

The c-di-GMP signaling network is widespread in bacteria and plays a crucial role in signal perception and regulation [19]. While there has been significant progress in understanding this network in the cytoplasm of bacteria, limited advancements have been made in identifying the c-di-GMP signaling network in other bacterial compartments [18, 36]. In this study, we identified a DGC, BtsD, as a thermosensor for the body temperature of mammalian hosts. BtsD detects 37°C to decrease its c-di-GMP generation, reducing the periplasmic c-di-GMP level. A c-di-GMP signaling cascade is then initiated within the BtsD domains, where the decreased periplasmic c-di-GMP level releases the N-terminal region of BtsD from c-di-GMP binding, allowing it to interact with the periplasmic sensor domain of BtsK. This binding event activates the TCS BtsKR by reducing BtsK’s autokinase activity, subsequently lowering BtsK′s phosphorylation level and hindering its phosphotransfer to BtsR, ultimately leading to a decrease in phosphorylation of BtsR. As a result, BtsR, acting as a transcriptional activator, upregulates the expression of *sod1-3*, a process necessary though not solely responsible for the pathogenicity and *in vivo* adaptability of *S*. *maltophilia* infecting *G*. *mellonella* larvae at 37°C (Fig 6).

Traditionally, it has been believed that c-di-GMP primarily operates within the cytoplasm of bacteria [37]. Here, we report for the first time the presence of a local c-di-GMP pool in the periplasm of *S*. *maltophilia*. Water-soluble solutes in bacterial cytoplasm, with a molecular weight greater than 600 Daltons [38,39], remain in the periplasm to form a compartmentalized pool once transported there through specific transportation systems or by interacting with certain periplasm-localized proteins. Given the role of BtsD in regulating periplasmic c-d-GMP levels (Fig 2C), along with its characteristics, including the c-di-GMP generation and binding activity (Figs 2C, 2D, 5C, S9A, and S9B), periplasmic localization (Fig 2B), and the absence of detectable GTP in the periplasm (S4 Fig), it is logical to speculate that BtsD generates c-di-GMP in the cytoplasm and transports c-di-GMP to the periplasm, though further evidence is required. This aligns with the current understanding of the periplasm, where reactions occurring in this space-such as protein hydrolysis by proteases, nucleic acid cleavage by nucleases, and dephosphorylation by phosphatases-are powered independently of NTPs, relying on the intrinsic chemical energy within their substrates and the structural configuration of their active sites [38], indicating that NTP pools are not required in the periplasm. Additionally, generating and binding c-di-GMP via a single protein module might be a strategy to maintain an effective local c-di-GMP pool for regulation. Furthermore, the critical role of the periplasmic c-di-GMP level in regulating bacterial adaptability and pathogenicity in response to temperature changes, underscores the importance of a local c-di-GMP pool over a global one.

DGCs that detect specific stimuli typically employ specific sensor domains to perceive signals. For instance, DGCs with globin domains in *Escherichia coli* use these domains to bind specifically with oxygen and enhance their synthetase activity [40]. DgcZ, a DGC from *E*. *coli*, utilizes its regulatory chemoreceptor zinc-binding (CZB) domain to sense hypochlorous acid (HOCl) and boost its cyclase activity [41]. Interestingly, we identified the importance of the GGDEF domain in enabling BtsD to sense temperature changes since that the solo GGDEF domain of BtsD changes its cyclase activity in response to temperature changes (S5B Fig), indicating its potential sensory role for the temperature. However, further analysis of the dynamic conformational changes of BtsD in response to temperature alterations is necessary to uncover the underlying thermosensing mechanism. Additionally, unlike most DGCs active in their dimers, BtsD is active in its tetramers (S5A Fig). Accompanying the findings that the GGDEF domain is irreplaceable for the role of BtsD in perceiving temperature changes to alter its cyclase activity (S5C Fig), and that FN3 domain is critical for the cyclase activity of BtsD (S9C Fig), it is believed that there is a complex interaction between domains of BtsD, though details are needed to be uncovered by further study.

The c-di-GMP system is often reported to be interlinked to the two-component signal transduction system (TCS) in a manner that the RR of the TCS acts as a c-di-GMP metabolizing enzyme to regulate cellular c-di-GMP levels. In such a condition, the TCS is activated by extracellular stimuli and transforms them into cellular c-di-GMP levels [42,43]. However, in this case, we report a reversed schedule of the two systems in the interlinked system with the c-di-GMP signaling upstream of the TCS. BtsD detects temperature changes to alter its c-di-GMP synthetase activity, resulting in a decrease in the periplasmic c-di-GMP level (Fig 2). The reduced c-di-GMP level releases BtsD′s N-terminal free from binding with c-di-GMP, and resets it to bind to BtsK′s sensor domain, thereby activating BtsK as well as BtsR (Fig 5). The finding expands our understanding of the interaction between the c-di-GMP signaling system and the TCS.

## Materials and methods

### Bacterial strains, plasmids and culture conditions

Bacterial strains and recombinant plasmids utilized in this study are detailed in S3 Table. The bacteria were cultured in lysogeny broth (LB) medium (tryptone 10 g/L, yeast extract 5 g/L, NaCl 10 g/L) with agitation or on LB agar plates with appropriate antibiotics, except for those involving the preparation of electro-component cells cultured in 210 medium (sucrose 5 g/L, casein enzymatic hydrolysate 8 g/L, yeast extract 4 g/L, K_2_HPO_4_ 3 g/L, and MgSO_4_∙7H_2_O 0.3 g/L). The *Escherichia coli* strain DH5α was used as the host for constructing all recombinant plasmids in this study, while BL21(DE3) was utilized for expressing all recombinant proteins. *S*. *maltophilia* strains were typically cultured at 28°C, unless otherwise indicated at 37°C, whereas *E*. *coli* stains were incubated at 37°C, except for at 16°C during protein expression induction in BL21(DE3). Antibiotics were utilized at the specified concentrations: kanamycin 50 mg/L, ampicillin 100 mg/L, chloromycetin 34 mg/L, spectinomycin 150 mg/L.

### Construction of strains and recombinant plasmids

We adhered to the protocols outlined in our prior publications for the construction of recombinant plasmids, mutants, and genetic complementary strains [26,44]. The primers used can be found in S4 Table. In summary, in-frame deletion and insertional inactivation mutants were constructed through homologous double-crossover and single-crossover recombination methods using pK18mobsacB and pK18mob suicide vectors, respectively. Complemented strains were developed by introducing recombinant broad-host vectors, pBBR1MCS1 or pBBR1MCS2, containing the specified full-length gene inserted at the multiclonal sites, into *S*. *maltophilia*.

### Phenotypic characterization

The mortality of *G*. *mellonella* larvae and the survival rates of bacteria *in vivo* were assessed according to the instructions provided [45, 46]. *G*. *mellonella* larvae, weighing approximately 300 mg, were obtained from Tianjin Huiyude Biotech Company, Tianjin, China, and were stored in the dark at 4°C on woodchips until needed. *S*. *maltophilia* strains were cultured overnight, washed twice with phosphate-buffered saline (PBS, pH 7.4), and the bacterial cell concentrations were determined by plate count. A total of 1×10^6^ CFU of the specified bacterial cultures were injected to the hemolymph of the preincubated *G*. *mellonella* larvae at 28°C and 37°C for 2 hours. Following injection, the larvae were incubated at 28°C and 37°C, and their mortality was recorded at specific time points. For the measurement of bacterial *in vivo* survival, the hemolymph of the larvae was collected as described two hours after bacterial injection, and the viable bacteria were quantified by plate count. 15–20 larvae were used for the mortality assay for each bacterial strain, while 9 larvae per strain were used for measuring the *in vivo* survival.

### Protein expression and purification

As referenced earlier [25,42,44], we undertook the expression and purification of the proteins. To provide a brief overview, we employed *E*. *coli* BL21 (DE3) for protein expression, and used GST- and His_6_-tags for protein purification. Purification of His_6_-tagged proteins was conducted using an AKTA purifier system following its manuals. Following this, the His_6_-tagged proteins were directly utilized in molecular interaction or enzymatic assays, whereas the GST-tagged proteins underwent tag cleavage before use. Inverted membrane of BtsK and BtsK^H264A^ proteins were prepared as detailed in our previous studies. The purified proteins were concentrated using Centricon YM-10 columns (Millipore, Billerica, MA, USA), and the elution buffer was exchanged for a storage buffer for further use (50 mM Tris-HCl, pH 8.0; 0.5 mM EDTA; 50 mM NaCl; and 5% glycerol).

### Separation and collection of cell components

At OD_600_ = 0.6, *S*. *maltophilia* cells were harvested to isolate their cellular components following the procedures outlined in the pET system manual and previous references with some modifications [47]. Initially, the bacterial cells were gathered through centrifugation and rinsed twice with a buffer solution containing 30 mM Tris-HCl (pH 7.0) and 150 mM NaCl. Subsequently, the pellet was reconstituted in a buffer consisting of 10 mM Tris-HCl (pH 8.4), 200 mM MgCl_2_, 0.5 mg/mL lysozyme, and 1 mM PMSF, and gently agitated for 30 minutes at room temperature. The resulting supernatant was separated by centrifugation (11000 g, 15 minutes at 4°C), concentrated through vacuum freeze-drying, and utilized as the periplasmic fraction. Meanwhile, the pellet was suspended in a buffer made up of 30 mM Tris-HCl (pH 8.0), 10 mM MgCl_2_, and subjected to sonication for cell disruption. The cellular debris was then removed by low-speed centrifugation (5000 g, 10 minutes at 4°C), followed by collection of the supernatant through a high-speed centrifugation (200,000 g, 40 minutes at 4°C) for the cytoplasmic fraction. Finally, the remaining pellet was resuspended in 0.4% Triton-X100 overnight at 4°C to extract the inner-membrane components.

### Western blotting analysis

Western blotting analyses were conducted in accordance with the protocols described in our previous publications [44]. Particularly, following the transfer of proteins to the membranes, we sectioned the membranes into segments and employed specific antibody for each segment to detect the target proteins. Anti-his monoclonal antibody was employed for detecting His-tagged BtsD and its recombinant proteins, while anti-HA was utilized for the detection of HA-tagged β-lactamase. The polyclonal antibodies targeting GL004030 and RNAP were generated through immunizing rats or rabbits as documented [44] and subsequently employed for their detection. After transferring proteins to the membranes, we trimmed the membranes into several parts according to sizes of the proteins to be tested and used the corresponding antibodies for the detection. The immunoblots were visualized using StarSignal Plus Reagent (GenStar, Beijing, China).

### Extraction and quantification of periplasmic c-di-GMP and GTP levels

Extraction of the bacterial periplasmic fraction was conducted following the procedures reported with modifications [47]. *S*. *maltophilia* cells at OD_600_ = 0.6 were incubated at 28°C and 37°C, respectively, for two hours, and subsequently collected by centrifugation at 4°C. The bacterial cells were washed twice with the pre-cooled buffer (30 mM Tris-HCl, pH 7.0, 150 mM NaCl). Afterward, the cells were numbered by plate count, followed by centrifugation and resuspension in the pre-cooled buffer (10 mM Tris-HCl, pH 8.4, 200 mM MgCl_2_, 0.5 mg/mL lysozyme, and 1 mM PMSF), while gently shaking for 2 hours at 4°C. The resulting supernatant was collected by centrifugation (11000 g, 15 min at 4°C) and divided into two portions. One portion was used for Western blot analysis to check for potential contamination by cytosolic components and the other was treated at 95°C for 10 min to inactivate all possible enzymes. The treated supernatant was then centrifuged to remove any possible pellets of inactivated proteins, concentrated via vacuum-freeze drying, and subsequently resuspended in water to quantify c-di-GMP and GTP using reverse phase or HSS T3 column-coupled HPLC-MS/MS[42]. The relative concentrations of c-di-GMP were calculated based on the cell numbers used for the periplasmic c-di-GMP extraction.

### *In vitro* analysis of diguanylate cyclase activity

The assays were carried out in accordance with our previous study with modifications [25, 42, 48]. 10 μM of the purified proteins, either the recombinant BtsD or DncV, were incubated with 1 mM GTP containing [α-^32^P] GTP (Perkin-Elmer) in the reaction buffer (300 mM NaCl, 50 mM Tris-HCl, pH 7.5, 20 mM MgCl_2_, 2 mM DTT) for two hours at 28°C and 37°C, respectively. Following this, the samples underwent a heating step at 75°C for 15 minutes to halt the reaction and were subsequently run in cellulose PEI TLC plates using a running buffer of [1:1.5 (vol/vol) of saturated (NH_4_)_2_SO_4_ and 1.5 M KH_2_PO_4_ (pH 3.6)] in order to separate the generated c-di-GMP. The c-di-GMP bands were then detected by autoradiographic signals, which were scanned using a Typhoon FLA700 (GE Healthcare).

### Polymeric structure analysis of BtsD using native gel electrophoresis

The purified BtsD^ΔSP^ proteins were incubated in the test buffer (2 mM HEPES, 20 mM K_2_HPO_4_, pH 7.9) and subjected to two distinct treatments. In the first treatment, the reactions were directly incubated at the specified temperatures for 2 hours. In the second treatment, the reactions underwent a gradual 30-min temperature increase from 0°C to the specified temperatures, followed by a 2-hour incubation at those temperatures. Subsequently, the samples were subjected to native gel analysis as reported [49].

### RNA-sequencing, RT-PCR, and qRT-PCR assay

The bacterial cultures with OD_600_ = 0.6 were divided into two groups, one of which was preincubated at 28°C for 2 hours, while the other group was preincubated at 37°C. Afterward, the bacterial cells from each sample were collected through centrifugation and used for total RNA extraction. The total RNA extraction from the bacterial cells was conducted using Trizol. To generate RNA libraries for sequencing, TruSeq RNA kits from Illumina (CA, USA) were utilized. The Illumina HiSeq 2000 platform was employed for RNA-seq, and three biological replicates were conducted. The resulting data was aligned and analyzed using SOAPaligner/SOAP2 and CLC Genomic Workbench for RNA-seq, respectively. The RNA-sequencing results are representatives of two independent repeats. For qRT-PCR, cDNA of tmRNA served as an internal control for amplification, and the procedure was performed as previously described [44]. To remove DNA contamination in total-RNA samples, DNA-free DNase (Ambion, Austen, TX, USA) was used. The first strand of cDNA was synthesized using random primers from Promega (Fitchburg, WI, USA). qRT-PCR was carried out in a DNA Engine Option 2 System (Bio-Rad) using Maxima SYBR Green (Fermentas, Vilnius, Lithuania). The primers used for qRT-PCR and RT-PCR can be found in S4 Table, and the latter was performed to amplify potential intergenic transcripts. The RT-PCR and qRT-PCR results are representatives of three independent repeats.

### ChIP-qPCR assay

The bacterial cultures were divided into two parts when their OD_600_ reached 0.6. One part was preincubated at 28°C for 2 hours, while the other was preincubated at 37°C for the same duration. Subsequently, ChIP-qPCR was conducted as detailed previously with modifications [44]. In brief, 1% formaldehyde was added to the cultures to conduct crosslinking, followed by a 10-minute incubation. This was then terminated by the addition of 0.5 M glycine and a further 5-minute incubation. For DNA preparation, the cultures were harvested, washed twice in 10 mL cold TBS buffer (20 mM Tris-HCl, pH 7.5; 150 mM NaCl), and resuspended in 1 mL lysis buffer (10 mM Tris, pH 8.0; 20% sucrose; 50 mM NaCl; 10 mM EDTA; 10 mg/mL lysozyme). Subsequently, they were sonicated using a Diagenode Bioruptor UCD-300 (Diagenode, Seraing, Belgium). The resulting supernatants were collected, mixed with IP buffer (50 mM HEPES-KOH, pH 7.5; 150 mM NaCl; 1 mM EDTA; 1% Triton X-100; 0.1% sodium deoxycholate; 0.1% SDS; 1 mM PMSF) at a 1:4 ratio, and the bacterial DNAs were fragmented to 200–500 bp using a Diagenode Bioruptor UCD-300 (Diagenode, Seraing, Belgium). Following centrifugation, a 100 μL aliquot was kept as the control DNA (input). For immunoprecipitation, a 20 μL 50% slurry of protein A sepharose and 2 μL of antibody (anti-His) were added to an 800 μL aliquot and incubated at 4°C overnight. The sepharose beads were collected by centrifugation and washed with IP buffer, IP buffer with 500 mM NaCl, wash buffer, and TE buffer, respectively. Immunoprecipitated chromatin was removed from the beads by adding 100 μL of elution buffer (50 mM Tris, pH 7.5; 10 mM EDTA; 1% SDS) and incubated for 10 min at 65°C. Samples were subsequently purified by RNase A, and proteinase K was used for reverse cross-linking. Finally, the DNA was purified using a PCR purification kit (Qiagen, Duesseldorf, Germany). For qPCR, 1 μL of the eluted DNA and 1 μL of the control DNA were used as templates for real-time PCR with appropriate primers (S4 Table). The quantities of the captured DNA were normalized to the control DNA, as mentioned in previous studies.

### Phosphorylation assays

The *in vitro* phosphorylation assays were performed according to previous studies [42, 44, 48]. In the autokinase activity test, 5 μM BtsK or its recombinant protein was incubated with 20 μM ATP containing 10 μCi [γ-^32^P]-ATP (PerkinElmer, Norwalk, CT, USA) in the autophosphorylation buffer (50 mM Tris-HCl, pH 7.8, 2 mM DTT, 25 mM NaCl, 25 mM KCl, 5 mM MgCl_2_) at either 28°C or 37°C, as indicated. For the phosphotransfer test, BtsK or its recombinant protein was first autophosphorylated and then incubated with 40 μM ThrR or its recombinant protein for forty-five seconds. If necessary, a preincubation of BtsK or its recombinant protein with 5 μM c-di-GMP, or 2 μM BtsD or its recombinant protein, or a mixture of c-di-GMP and the protein was performed before the phosphorylation assays. The reactions were stopped by adding SDS-PAGE loading buffer. The proteins in the samples were separated using 12% or 15% SDS-PAGE gels. The isotopic signal was detected using a Phosphor Image system Typhoon FLA7000 (Amersham Biosciences, Bath, UK).

### MST assays

The MST assays were performed according to the methods described in references [42, 48]. In brief, BtsD^Δ(SP-GGDEF)^ was expressed and purified as mentioned and Sensor^BtsK^ was synthesized. Both of them were labeled using the Monolith NT.115 Protein Labeling Kit Red NHS (a dye that reacts with amines, specifically designed for MicroScale Thermophoresis). They were then incubated with c-di-GMP in PBS buffer (pH 6.5) to check their binding interaction with c-di-GMP. BtsD^Δ(SP-GGDEF)^ was preincubated with c-di-GMP, and the resulting mixture and BtsD^Δ(SP-GGDEF)^ alone were used as ligands, respectively, to investigate their binding with the labeled Sensor^BtsK^. The interactions between the proteins and the ligands were detected using a Monolith NT.115 MicroScale Thermophoresis instrument (manufactured by NanoTemper Technologies, GMBH, Munich, Germany) and Monolith NT.115 Series capillaries. The curves obtained from the measurements were fitted and the dissociation constant (*K*_*d*_) values were calculated using the KD Fit function of the NanoTemper Analysis Software Version 2.3.

### Molecular docking analyses

The molecular docking analyses were performed following the established protocols with certain modifications outlined in references [25]. The structure of BtsD^Δ(SP-GGDEF)^ was obtained from the AlphaFold Protein Structure Database and was used for molecular docking and structural difference analyses. The structure of c-di-GMP was constructed and optimized using ChemDraw and MOPAC software. Autodock 4.2.6 software was utilized for the docking calculations, and the lowest energy conformation was selected. Subsequently, Amber14 software was employed to obtain an optimized conformation based on this result.

### Sequence alignment

A BlastP analysis was performed using the NCBI databases to find homologs of BtsD, with the parameters set to a minimum of 70% coverage and 30% identity. ClustalW was employed for sequence alignment, and the Maximum likelihood method was used for phylogenetic analysis. All sequences used in the analysis were downloaded from the NCBI databases.

### EMSA

The EMSA was performed following the established protocols with certain modifications outlined in references [25, 26]. The probes used in this assay were amplified via PCR and labeled with [γ-^32^P] ATP using T4 polynucleotide kinase. Free [γ-^32^P] ATP was then removed using a ProbeQuant G-50 column (GE, New York, NY, USA). To carry out the assay, 400 ng of BtsR proteins were incubated with 2 fmol of labeled probes in the EMSA reaction buffer (10 mM Tris-HCl, pH 7.0, 50 mM KCl, 1 mM DTT, 2.5% glycerol) for 40 minutes at room temperature. For the preparation of phosphorylated BtsR, BtsK and BtsR were initially incubated in a phosphorylation reaction system for 10 minutes, with BtsR incubated with BtsK^H264A^ serving as negative controls. The phosphorylation reaction was then stopped by exchanging the phosphorylation buffer to the EMSA reaction buffer using a PD Spin Trap G-25 column. The resulting mixture, consisting of phosphorylated BtsR, was used to assess its binding affinity with the labeled probes. The reactions were terminated by adding 2 mM EDTA. The samples were mixed with 4 μL of 40% sucrose and loaded into a 4% native polyacrylamide gel electrophoresis (PAGE) gel. Electrophoresis was conducted at 100 V for approximately 50 minutes using 0.5 × TBE buffer. Subsequently, the gel was placed into a zip bag, and the signals were recorded using a Typhoon FLA700 imaging system (GE Healthcare).

### Statistical analysis

GraphPad Prism Software (GraphPad Prism 8.0.2) or Microsoft Excel 2021 was used to perform statistical analyses. Data from RNA-seq were analyzed using Burrows-wheeler (Bowtie2), RSEM, and DESeq2. All other experiments were using the two-tailed unpaired Student′s *t*-test, and data are presented as mean ± SD. Statistical significance was defined as *p* < 0.05

## Supporting information

S1 Fig *G*. *mellonella* larvae killing assay, demonstrating larval mortality at 28°C and 37°C. Panels **(A)** through **(F)** correspond to Figs 1A, 3D, 3E, and 4A, respectively. The figure presents the original data dots from three independent replicates along with the associated errors. The strains used are described above.
(TIF)

S2 Fig *btsD* deletion does not significantly change the growth curve of *S*. *maltophilia*. The shown data is represented as mean ± SD of three independent replicates. The used strains are described above.
(TIF)

S3 Fig Deletion of the SP of BtsD significantly reduced the swimming motility of *S*. *maltophilia*. The swimming motilities of the specified strains were investigated, and representative data from three independent replicates are presented. CbtsD^ΔSP^: the complementary stain constitutively expressing the recombinant *btsD* with the SP-encoding sequences deleted in the *btsD* deletion background. Details of the other strains are described above.
(TIF)

S4 Fig No detectable GTP was identified in the periplasm of *S*. *maltophilia*. The isolated periplasmic fraction of *S*. *maltophilia*, verified to be free of contamination, was subjected to LC-MS/MS analysis for GTP concentration measurement. The isolated cytoplasmic fraction served as a positive control. Cyto. denotes the LC-MS/MS analysis result of the isolated cytoplasmic fraction, Peri. represents the isolated periplasmic fraction, and Stan. corresponds to the GTP standards. Representative results from independent replicates with comparable outcomes are presented.
(TIF)

S5 Fig Critical roles of BtsD′s tetrameric form and GGDEF domain in its diguanylate cyclase activity. **(A)** Analysis of the polymeric forms of BtsD using Native gels at 37°C and 28°C. Slow represents samples subjected to a 30-min temperature upshift from 0°C to the specified temperatures, while Fast denotes samples directly incubated at the specified temperatures. **(B)** The GGDEF domain of BtsD reduces its c-di-GMP synthesis activity at 37°C compared to 28°C. **(C)** Replacing the GGDEF domain of BtsD by that of WspR abolishes the diguanylate cyclase activity of recombinant protein. All reactions in (B) and (C) were conducted at the specified temperatures for 2 hours, followed by TLC analyses. Blank denotes the reaction without protein, indicating the location of GTP bands. WspR is a diguanylate cyclase active at 37°C, encoded by *P*. *aeruginosa*. BtsD^sub^ represents the recombinant BtsD with its GGDEF domain replaced by the GGDEF domain of WspR. All other recombinant proteins are described in Fig 2A. All presented data are representative of three independent repetitions, yielding consistent outcomes.
(TIF)

S6 Fig Sequence alignment highlights critical sites in the GGDEF domain of BtsD. The sequence alignment of the GGDEF domain of BtsD was analyzed using ClustalW, comparing it to the GGDEF domains of other diguanylate cyclases active at 37°C. Amino acid residues with same or similar characteristics are color-coded. Critical variations are highlighted with black boxes, and their specific locations in BtsD are annotated.
(TIF)

S7 Fig BtsD′s SP is conserved in some homologs from various *Stenotrophomonas* species and two *Pseudomonas* species. **(A)** The ClustalW analysis of the SP sequences between BtsD and its homologs. The signal peptide regions are highlighted in a black box, with several representative *Stenotrophomonas* species, both conserved and varied, listed. (B) The phylogenetic tree, based on the sequence alignments shown in (A), was constructed using the maximum likelihood method.
(TIF)

S8 Fig GO and KEGG analyses on genes regulated by BtsD at 37°C compared to 28°C. The details and proportions of the gene groups classified by the first and secondary levels of GO **(A)** and KEGG **(B)** classification.
(TIF)

S9 Fig Involvement of BtsD′s FN3 domain in c-di-GMP binding and regulation of cyclase activity. **(A)** and **(B)** Molecular docking analysis showing the binding pocket location, docking sites and intermolecular forces between BtsD^Δ(SP-GGDEF)^ and c-di-GMP. In (A), the protein is in blue with the exception of the FN3 domain in yellow, while the c-di-GMP molecule is showcased in gray. In (B), the amino acid residues participating in hydrogen bonding is highlighted in yellow, with the hydrogen bonds depicted as yellow dotted lines. **(C)** Deletion of the FN3 domain significantly decreased the diguanylate cyclase activity of recombinant BtsD. The reactions were conducted at the specified temperatures for 2 hours, followed by TLC analyses. Blank denotes the reaction without protein, indicating the location of GTP bands. All the recombinant proteins are described in Fig 2A. The presented data are representative of three independent repetitions, yielding consistent outcomes.
(TIF)

S1 Table Genes significantly changed upon the temperature change. (DOCX)

S2 Table Gene annotations by GO and KEGG. (DOCX)

S3 Table Bacterial strains and plasmids used in this study. (DOCX)

S4 Table Primers used in this study. (DOCX)

S1 Data Numerical values used to generate graphs. (XLSX)
