# Supplementary figures and images for: Stenotrophomonas maltophilia uses a c-di-GMP module to sense the mammalian body temperature during infection
Source: PLoS Pathog. 2024 Sep 4;20(9):e1012533. doi: 10.1371/journal.ppat.1012533 (PMC11404848; doi:10.1371/journal.ppat.1012533)

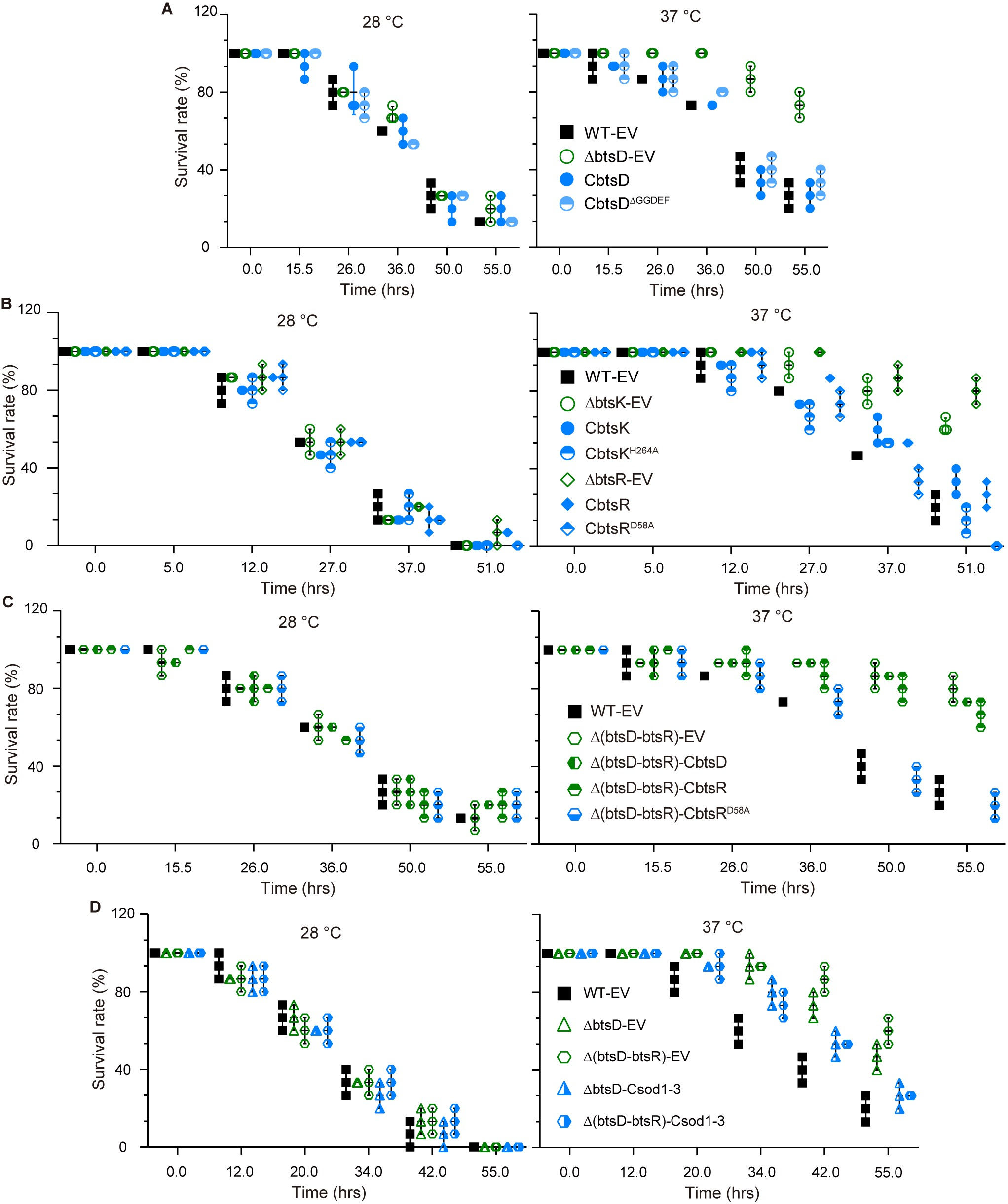

Supplement: S1 Fig — Panels (A) through (F) correspond to Figs 1A, 3D, 3E, and 4A, respectively. The figure presents the original data dots from three independent replicates along with the associated errors. The strains used are described above. (TIF) [file ppat.1012533.s001.tif]

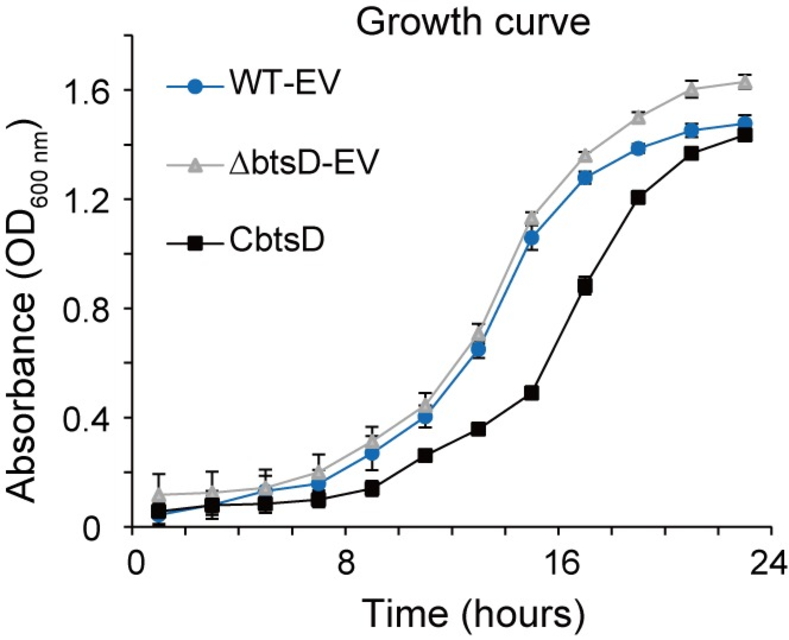

Supplement: S2 Fig — The shown data is represented as mean ± SD of three independent replicates. The used strains are described above. (TIF) [file ppat.1012533.s002.tif]

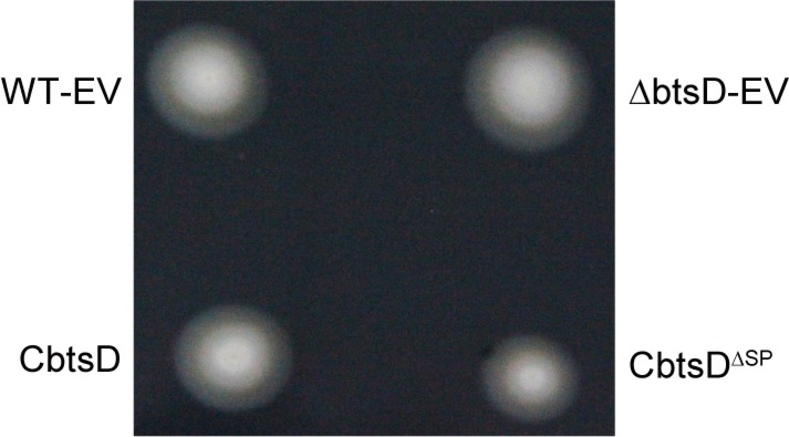

Supplement: S3 Fig — The swimming motilities of the specified strains were investigated, and representative data from three independent replicates are presented. CbtsDΔSP: the complementary stain constitutively expressing the recombinant btsD with the SP-encoding sequences deleted in the btsD deletion background. Details of the other strains are described above. (TIF) [file ppat.1012533.s003.tif]

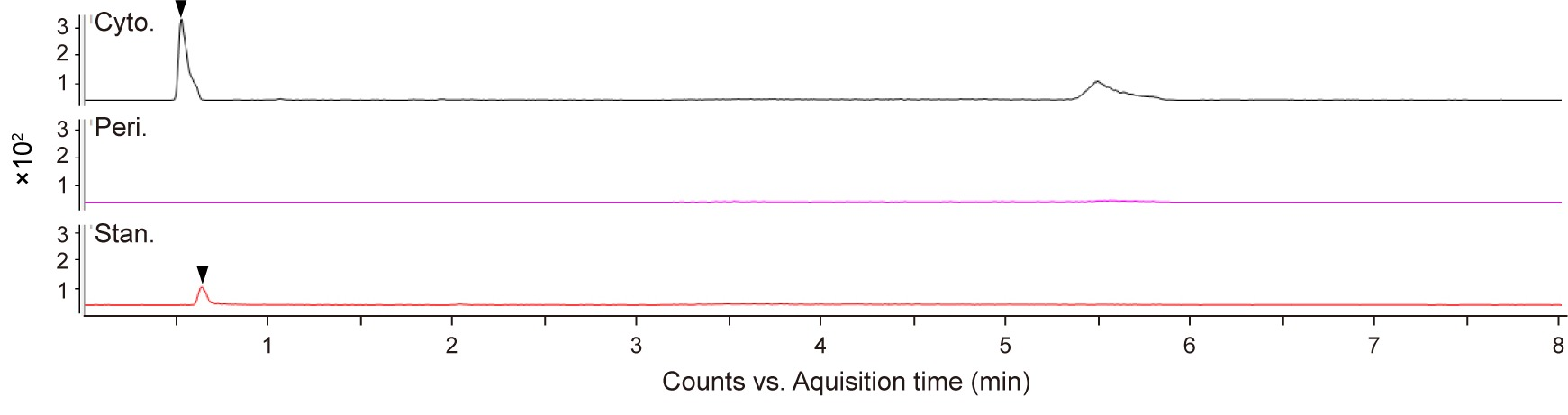

Supplement: S4 Fig — The isolated periplasmic fraction of S. maltophilia, verified to be free of contamination, was subjected to LC-MS/MS analysis for GTP concentration measurement. The isolated cytoplasmic fraction served as a positive control. Cyto. denotes the LC-MS/MS analysis result of the isolated cytoplasmic fraction, Peri. represents the isolated periplasmic fraction, and Stan. corresponds to the GTP standards. Representative results from independent replicates with comparable outcomes are presented. (TIF) [file ppat.1012533.s004.tif]

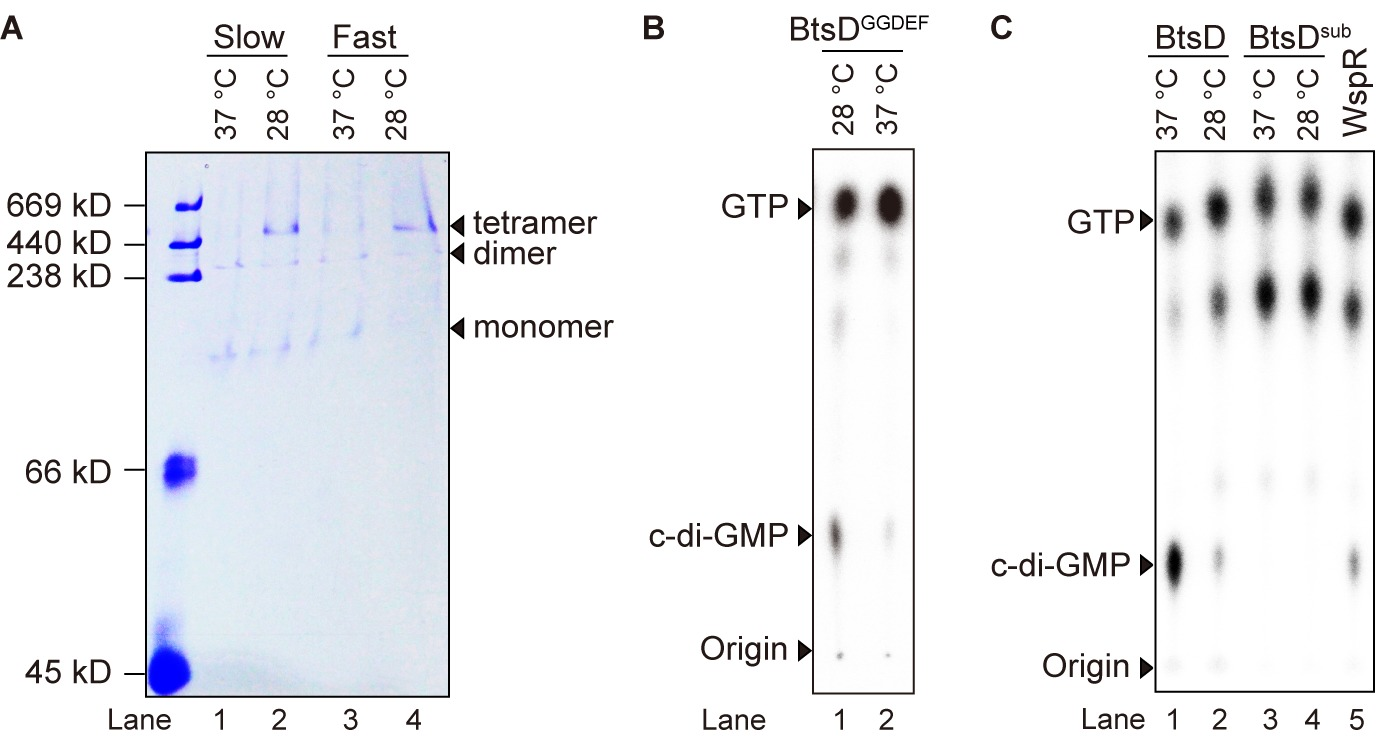

Supplement: S5 Fig — (A) Analysis of the polymeric forms of BtsD using Native gels at 37°C and 28°C. Slow represents samples subjected to a 30-min temperature upshift from 0°C to the specified temperatures, while Fast denotes samples directly incubated at the specified temperatures. (B) The GGDEF domain of BtsD reduces its c-di-GMP synthesis activity at 37°C compared to 28°C. (C) Replacing the GGDEF domain of BtsD by that of WspR abolishes the diguanylate cyclase activity of recombinant protein. All reactions in (B) and (C) were conducted at the specified temperatures for 2 hours, followed by TLC analyses. Blank denotes the reaction without protein, indicating the location of GTP bands. WspR is a diguanylate cyclase active at 37°C, encoded by P. aeruginosa. BtsDsub represents the recombinant BtsD with its GGDEF domain replaced by the GGDEF domain of WspR. All other recombinant proteins are described in Fig 2A. All presented data are representative of three independent repetitions, yielding consistent outcomes. (TIF) [file ppat.1012533.s005.tif]

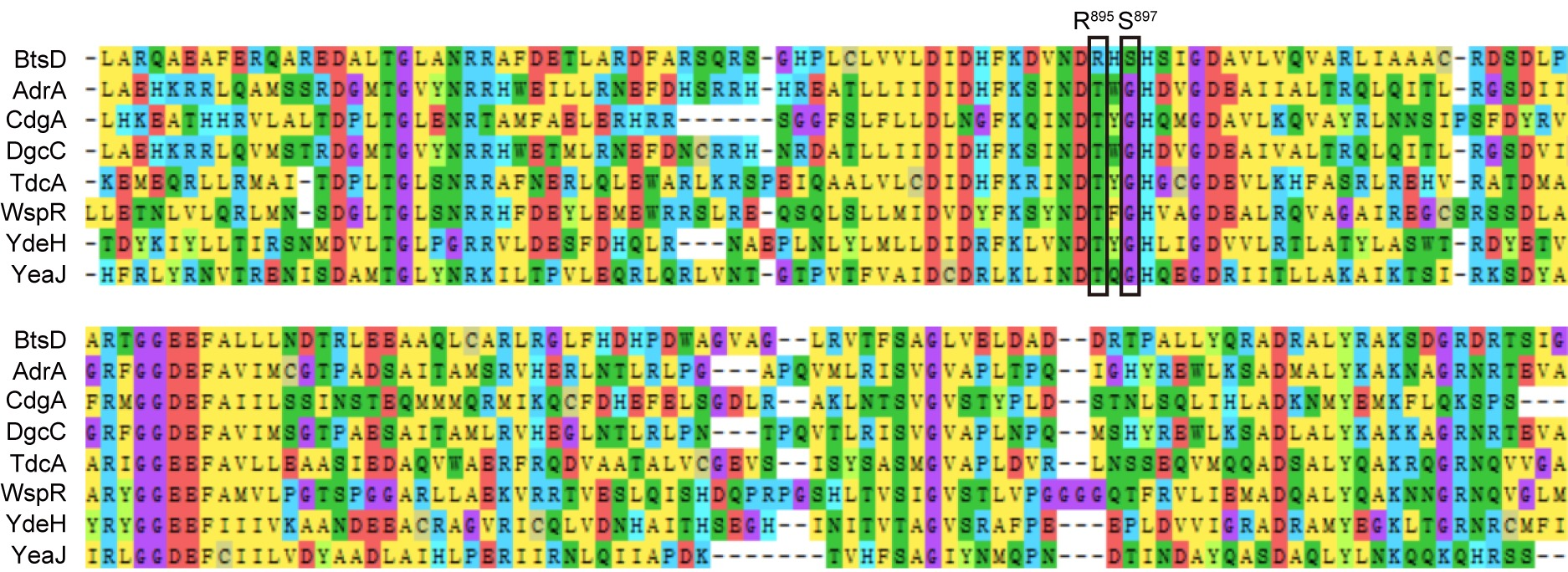

Supplement: S6 Fig — The sequence alignment of the GGDEF domain of BtsD was analyzed using ClustalW, comparing it to the GGDEF domains of other diguanylate cyclases active at 37°C. Amino acid residues with same or similar characteristics are color-coded. Critical variations are highlighted with black boxes, and their specific locations in BtsD are annotated. (TIF) [file ppat.1012533.s006.tif]

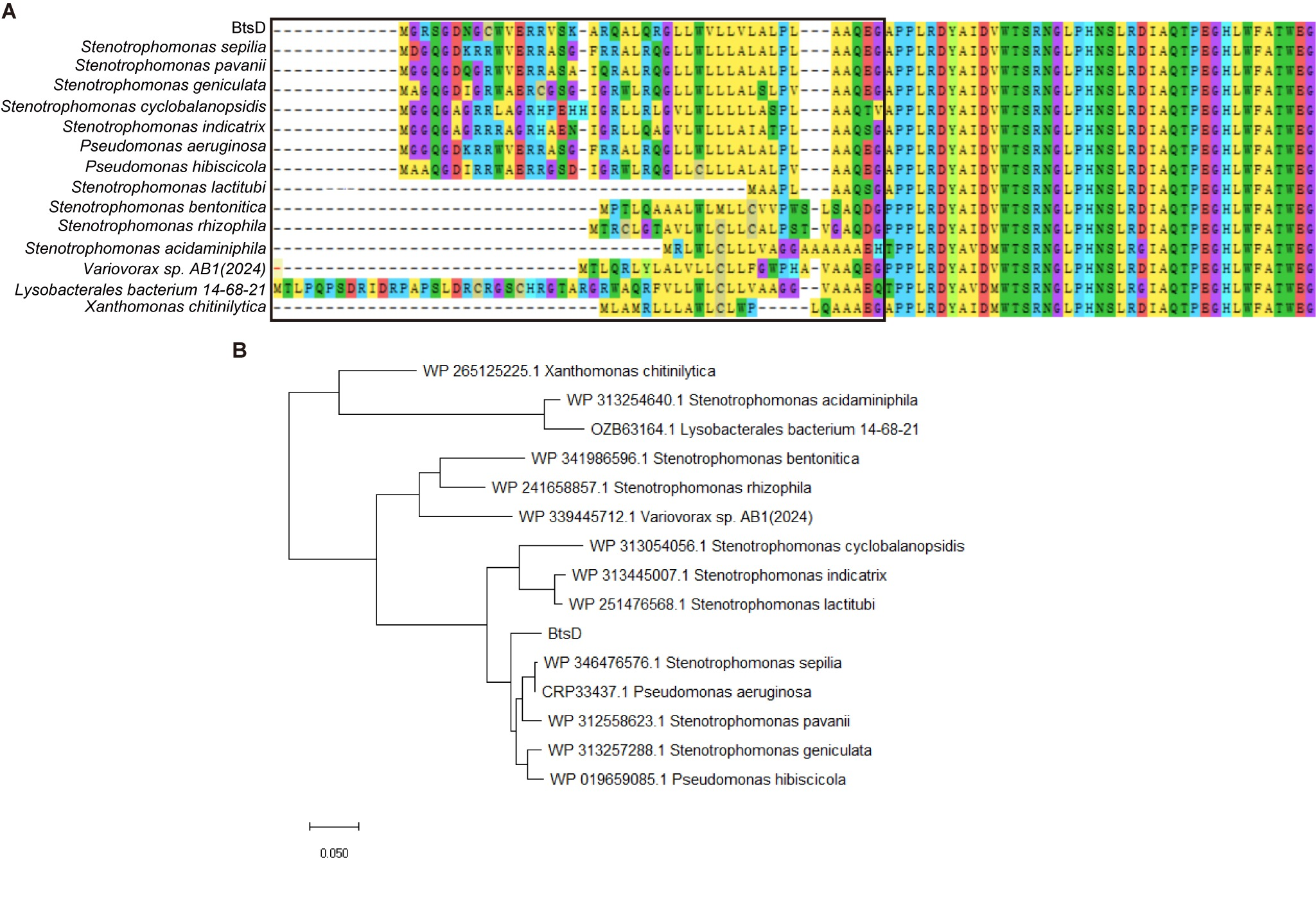

Supplement: S7 Fig — (A) The ClustalW analysis of the SP sequences between BtsD and its homologs. The signal peptide regions are highlighted in a black box, with several representative Stenotrophomonas species, both conserved and varied, listed. (B) The phylogenetic tree, based on the sequence alignments shown in (A), was constructed using the maximum likelihood method. (TIF) [file ppat.1012533.s007.tif]

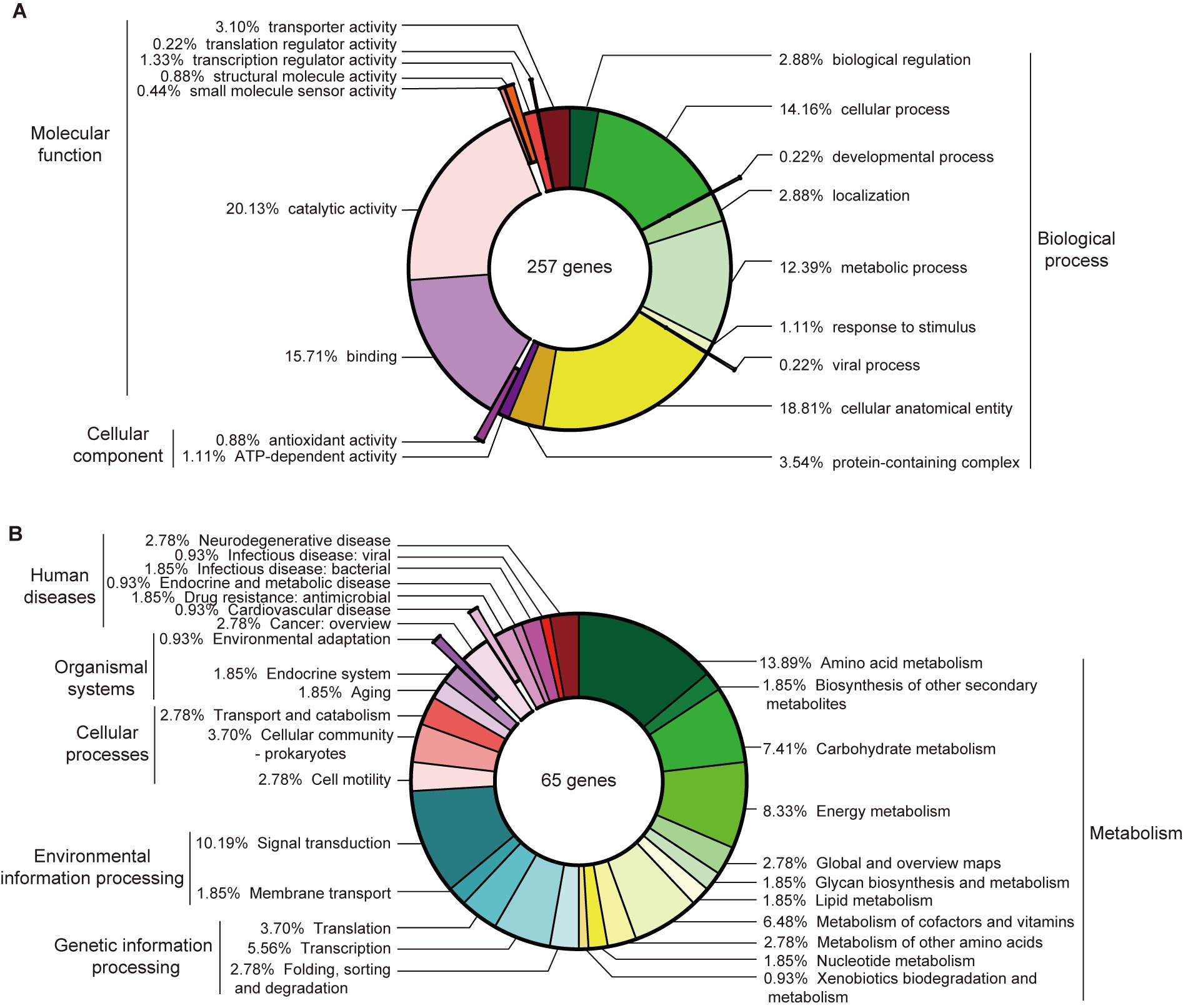

Supplement: S8 Fig — The details and proportions of the gene groups classified by the first and secondary levels of GO (A) and KEGG (B) classification. (TIF) [file ppat.1012533.s008.tif]

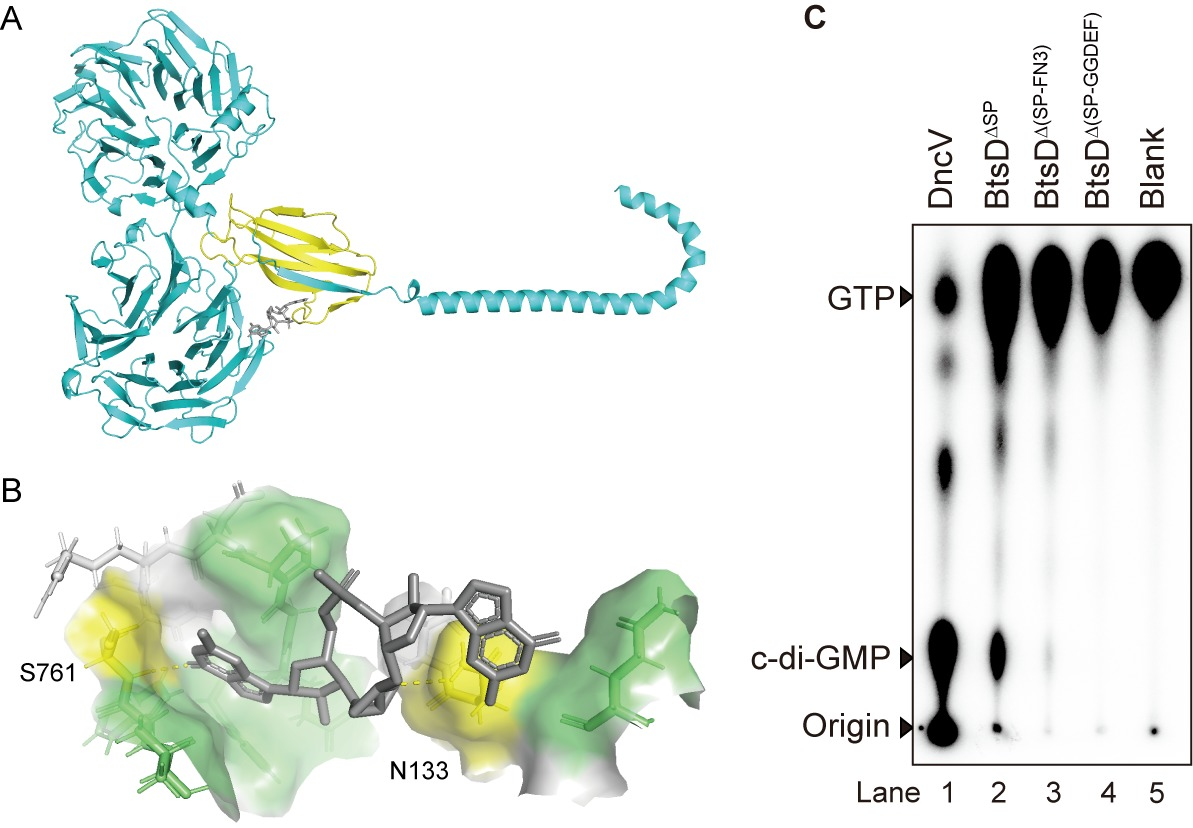

Supplement: S9 Fig — (A) and (B) Molecular docking analysis showing the binding pocket location, docking sites and intermolecular forces between BtsDΔ(SP-GGDEF) and c-di-GMP. In (A), the protein is in blue with the exception of the FN3 domain in yellow, while the c-di-GMP molecule is showcased in gray. In (B), the amino acid residues participating in hydrogen bonding is highlighted in yellow, with the hydrogen bonds depicted as yellow dotted lines. (C) Deletion of the FN3 domain significantly decreased the diguanylate cyclase activity of recombinant BtsD. The reactions were conducted at the specified temperatures for 2 hours, followed by TLC analyses. Blank denotes the reaction without protein, indicating the location of GTP bands. All the recombinant proteins are described in Fig 2A. The presented data are representative of three independent repetitions, yielding consistent outcomes. (TIF) [file ppat.1012533.s009.tif]
