# Supplementary material for: Stenotrophomonas maltophilia uses a c-di-GMP module to sense the mammalian body temperature during infection
Source: PLoS Pathog. 2024 Sep 4;20(9):e1012533. doi: 10.1371/journal.ppat.1012533 (PMC11404848; doi:10.1371/journal.ppat.1012533)
Supplement: S1 Table — (DOCX) [file ppat.1012533.s010.docx]

| **S1 Table. Genes significantly changed upon the temperature change** | |
| --- | --- |
| Gene_id | Fold (37 °C/28 °C) |
| Genes significantly changed in WT | |
| *S.maltophilia.CGMCC.1.1788GL000101* | 0.47 ± 0.03 |
| *S.maltophilia.CGMCC.1.1788GL000215* | 2.88 ± 1.03 |
| *S.maltophilia.CGMCC.1.1788GL000224* | 2.39 ± 0.21 |
| *S.maltophilia.CGMCC.1.1788GL000265* | 0.20 ± 0.12 |
| *S.maltophilia.CGMCC.1.1788GL000268* | 0.42 ± 0.20 |
| *S.maltophilia.CGMCC.1.1788GL000278* | 0.50 ± 0.15 |
| *S.maltophilia.CGMCC.1.1788GL000280* | 0.39 ± 0.13 |
| *S.maltophilia.CGMCC.1.1788GL000319* | 0.50 ± 0.12 |
| *S.maltophilia.CGMCC.1.1788GL000393* | 3.00 ± 1.19 |
| *S.maltophilia.CGMCC.1.1788GL000464* | 0.49 ± 0.21 |
| *S.maltophilia.CGMCC.1.1788GL000521* | 0.42 ± 0.16 |
| *S.maltophilia.CGMCC.1.1788GL000592* | 2.98 ± 1.38 |
| *S.maltophilia.CGMCC.1.1788GL000691* | 0.50 ± 0.04 |
| *S.maltophilia.CGMCC.1.1788GL000711* | 2.59 ± 0.51 |
| *S.maltophilia.CGMCC.1.1788GL000793* | 2.84 ± 1.09 |
| *S.maltophilia.CGMCC.1.1788GL000798* | 2.51 ± 0.15 |
| *S.maltophilia.CGMCC.1.1788GL000834* | 3.25 ± 1.56 |
| *S.maltophilia.CGMCC.1.1788GL000996* | 4.25 ± 0.35 |
| *S.maltophilia.CGMCC.1.1788GL001053* | 0.19 ± 0.09 |
| *S.maltophilia.CGMCC.1.1788GL001200* | 0.50 ± 0.04 |
| *S.maltophilia.CGMCC.1.1788GL001422* | 0.50 ± 0.12 |
| *S.maltophilia.CGMCC.1.1788GL001473* | 3.40 ± 1.20 |
| *S.maltophilia.CGMCC.1.1788GL001494* | 2.86 ± 0.10 |
| *S.maltophilia.CGMCC.1.1788GL001513* | 0.40 ± 0.07 |
| *S.maltophilia.CGMCC.1.1788GL001545* | 2.96 ± 1.21 |
| *S.maltophilia.CGMCC.1.1788GL001546* | 3.03 ± 1.38 |
| *S.maltophilia.CGMCC.1.1788GL001719* | 2.63 ± 0.23 |
| *S.maltophilia.CGMCC.1.1788GL001721* | 3.62 ± 0.15 |
| *S.maltophilia.CGMCC.1.1788GL001735* | 3.28 ± 1.13 |
| *S.maltophilia.CGMCC.1.1788GL001788* | 3.03 ± 1.70 |
| *S.maltophilia.CGMCC.1.1788GL001794* | 2.89 ± 1.65 |
| *S.maltophilia.CGMCC.1.1788GL001822* | 2.90 ± 1.14 |
| *S.maltophilia.CGMCC.1.1788GL001832* | 0.50 ± 0.05 |
| *S.maltophilia.CGMCC.1.1788GL001895* | 3.89 ± 2.30 |
| *S.maltophilia.CGMCC.1.1788GL001953* | 0.50 ± 0.08 |
| *S.maltophilia.CGMCC.1.1788GL002008* | 0.50 ± 0.12 |
| *S.maltophilia.CGMCC.1.1788GL002037* | 3.03 ± 1.33 |
| *S.maltophilia.CGMCC.1.1788GL002046* | 2.96 ± 1.57 |
| *S.maltophilia.CGMCC.1.1788GL002113* | 11.62 ± 4.20 |
| *S.maltophilia.CGMCC.1.1788GL002180* | 0.50 ± 0.00 |
| *S.maltophilia.CGMCC.1.1788GL002185* | 2.89 ± 0.59 |
| *S.maltophilia.CGMCC.1.1788GL002187* | 2.86 ± 1.32 |
| *S.maltophilia.CGMCC.1.1788GL002194* | 6.31 ± 2.86 |
| *S.maltophilia.CGMCC.1.1788GL002195* | 5.04 ± 2.68 |
| *S.maltophilia.CGMCC.1.1788GL002196* | 9.65 ± 1.99 |
| *S.maltophilia.CGMCC.1.1788GL002197* | 4.07 ± 1.95 |
| *S.maltophilia.CGMCC.1.1788GL002198* | 3.72 ± 1.54 |
| *S.maltophilia.CGMCC.1.1788GL002206* | 2.84 ± 0.76 |
| *S.maltophilia.CGMCC.1.1788GL002305* | 0.15 ± 0.03 |
| *S.maltophilia.CGMCC.1.1788GL002308* | 0.12 ± 0.00 |
| *S.maltophilia.CGMCC.1.1788GL002324* | 0.34 ± 0.16 |
| *S.maltophilia.CGMCC.1.1788GL002458* | 0.50 ± 0.06 |
| *S.maltophilia.CGMCC.1.1788GL002665* | 0.50 ± 0.28 |
| *S.maltophilia.CGMCC.1.1788GL002671* | 0.50 ± 0.24 |
| *S.maltophilia.CGMCC.1.1788GL002732* | 2.91 ± 0.48 |
| *S.maltophilia.CGMCC.1.1788GL002734* | 3.07 ± 0.08 |
| *S.maltophilia.CGMCC.1.1788GL002761* | 4.49 ± 2.41 |
| *S.maltophilia.CGMCC.1.1788GL002772* | 0.34 ± 0.18 |
| *S.maltophilia.CGMCC.1.1788GL002776* | 2.87 ± 0.68 |
| *S.maltophilia.CGMCC.1.1788GL002777* | 2.84 ± 0.49 |
| *S.maltophilia.CGMCC.1.1788GL002778* | 2.03 ± 0.38 |
| *S.maltophilia.CGMCC.1.1788GL002806* | 0.50 ± 0.23 |
| *S.maltophilia.CGMCC.1.1788GL002940* | 4.24 ± 2.56 |
| *S.maltophilia.CGMCC.1.1788GL002955* | 2.72 ± 0.08 |
| *S.maltophilia.CGMCC.1.1788GL002980* | 3.24 ± 0.83 |
| *S.maltophilia.CGMCC.1.1788GL003013* | 4.30 ± 2.56 |
| *S.maltophilia.CGMCC.1.1788GL003122* | 0.50 ± 0.05 |
| *S.maltophilia.CGMCC.1.1788GL003134* | 2.90 ± 1.24 |
| *S.maltophilia.CGMCC.1.1788GL003158* | 2.91 ± 1.38 |
| *S.maltophilia.CGMCC.1.1788GL003169* | 2.92 ± 0.97 |
| *S.maltophilia.CGMCC.1.1788GL003348* | 0.50 ± 0.11 |
| *S.maltophilia.CGMCC.1.1788GL003354* | 0.50 ± 0.12 |
| *S.maltophilia.CGMCC.1.1788GL003370* | 2.91 ± 1.49 |
| *S.maltophilia.CGMCC.1.1788GL003371* | 0.50 ± 0.08 |
| *S.maltophilia.CGMCC.1.1788GL003487* | 2.89 ± 0.83 |
| *S.maltophilia.CGMCC.1.1788GL003554* | 3.05 ± 1.17 |
| *S.maltophilia.CGMCC.1.1788GL003588* | 0.50 ± 0.04 |
| *S.maltophilia.CGMCC.1.1788GL003625* | 2.65 ± 0.99 |
| *S.maltophilia.CGMCC.1.1788GL003683* | 2.99 ± 1.85 |
| *S.maltophilia.CGMCC.1.1788GL003875* | 0.41 ± 0.20 |
| *S.maltophilia.CGMCC.1.1788GL003968* | 0.50 ± 0.16 |
| *S.maltophilia.CGMCC.1.1788GL004052* | 3.16 ± 1.51 |
| *S.maltophilia.CGMCC.1.1788GL004070* | 2.70 ± 0.84 |
| *S.maltophilia.CGMCC.1.1788GL004090* | 2.90 ± 1.17 |
| *S.maltophilia.CGMCC.1.1788GL004098* | 0.50 ± 0.10 |
| *S.maltophilia.CGMCC.1.1788GL004099* | 0.49 ± 0.07 |
| *S.maltophilia.CGMCC.1.1788GL004100* | 0.50 ± 0.05 |
| *S.maltophilia.CGMCC.1.1788GL004116* | 3.12 ± 1.77 |
| *S.maltophilia.CGMCC.1.1788GL004141* | 2.32 ± 0.47 |
| *S.maltophilia.CGMCC.1.1788GL004195* | 0.50 ± 0.10 |
| *S.maltophilia.CGMCC.1.1788GL004249* | 3.25 ± 0.51 |
| *S.maltophilia.CGMCC.1.1788GL004286* | 2.93 ± 0.57 |
| *S.maltophilia.CGMCC.1.1788GL004324* | 3.19 ± 0.95 |
| *S.maltophilia.CGMCC.1.1788GL004526* | 0.50 ± 0.00 |
| *S.maltophilia.CGMCC.1.1788GL004532* | 3.35 ± 1.81 |
| *S.maltophilia.CGMCC.1.1788GL004601* | 3.37 ± 0.64 |
| *S.maltophilia.CGMCC.1.1788GL004626* | 4.05 ± 1.44 |
| *novel0012* | 0.50 ± 0.05 |
| *novel0014* | 15.67 ± 0.11 |
| *novel0018* | 0.50 ± 0.17 |
| *novel0020* | 0.50 ± 0.18 |
| *novel0021* | 0.50 ± 0.14 |
| *novel0067* | 0.46 ± 0.09 |
| *novel0083* | 2.68 ± 0.19 |
| *novel0100* | 0.50 ± 0.25 |
| *novel0151* | 0.41 ± 0.17 |
| *novel0156* | 20.55 ± 0.80 |
| *novel0192* | 0.50 ± 0.02 |
| *novel0239* | 3.25 ± 0.04 |
| *novel0323* | 2.56 ± 0.15 |
| *novel0339* | 0.50 ± 0.13 |
| Genes significantly changed in ΔbtsD | |
| *S.maltophilia.CGMCC.1.1788GL000009* | 0.47 ± 0.08 |
| *S.maltophilia.CGMCC.1.1788GL000064* | 2.60 ± 0.93 |
| *S.maltophilia.CGMCC.1.1788GL000084* | 2.13 ± 0.22 |
| *S.maltophilia.CGMCC.1.1788GL000093* | 0.44 ± 0.03 |
| *S.maltophilia.CGMCC.1.1788GL000158* | 0.47 ± 0.02 |
| *S.maltophilia.CGMCC.1.1788GL000159* | 0.48 ± 0.05 |
| *S.maltophilia.CGMCC.1.1788GL000192* | 6.4 ± 0.97 |
| *S.maltophilia.CGMCC.1.1788GL000199* | 0.39 ± 0.04 |
| *S.maltophilia.CGMCC.1.1788GL000248* | 0.48 ± 0.19 |
| *S.maltophilia.CGMCC.1.1788GL000249* | 11.40 ± 3.93 |
| *S.maltophilia.CGMCC.1.1788GL000284* | 2.38 ± 0.37 |
| *S.maltophilia.CGMCC.1.1788GL000362* | 2.20 ± 0.52 |
| *S.maltophilia.CGMCC.1.1788GL000394* | 2.18 ± 0.26 |
| *S.maltophilia.CGMCC.1.1788GL000416* | 0.50 ± 0.22 |
| *S.maltophilia.CGMCC.1.1788GL000445* | 0.50 ± 0.04 |
| *S.maltophilia.CGMCC.1.1788GL000500* | 0.50 ± 0.20 |
| *S.maltophilia.CGMCC.1.1788GL000534* | 0.40 ± 0.02 |
| *S.maltophilia.CGMCC.1.1788GL000558* | 2.14 ± 0.42 |
| *S.maltophilia.CGMCC.1.1788GL000662* | 0.48 ± 0.07 |
| *S.maltophilia.CGMCC.1.1788GL000724* | 0.50 ± 0.02 |
| *S.maltophilia.CGMCC.1.1788GL000746* | 0.50 ± 0.14 |
| *S.maltophilia.CGMCC.1.1788GL000805* | 0.50 ± 0.14 |
| *S.maltophilia.CGMCC.1.1788GL000828* | 0.44 ± 0.01 |
| *S.maltophilia.CGMCC.1.1788GL000831* | 2.15 ± 0.08 |
| *S.maltophilia.CGMCC.1.1788GL000843* | 0.49 ± 0.14 |
| *S.maltophilia.CGMCC.1.1788GL000846* | 0.47 ± 0.01 |
| *S.maltophilia.CGMCC.1.1788GL000852* | 0.48 ± 0.04 |
| *S.maltophilia.CGMCC.1.1788GL000873* | 0.50 ± 0.06 |
| *S.maltophilia.CGMCC.1.1788GL000876* | 0.42 ± 0.02 |
| *S.maltophilia.CGMCC.1.1788GL000877* | 0.50 ± 0.05 |
| *S.maltophilia.CGMCC.1.1788GL000960* | 0.50 ± 0.08 |
| *S.maltophilia.CGMCC.1.1788GL000963* | 0.50 ± 0.07 |
| *S.maltophilia.CGMCC.1.1788GL000964* | 0.50 ± 0.01 |
| *S.maltophilia.CGMCC.1.1788GL000966* | 0.30 ± 0.01 |
| *S.maltophilia.CGMCC.1.1788GL000967* | 0.46 ± 0.02 |
| *S.maltophilia.CGMCC.1.1788GL000968* | 0.50 ± 0.10 |
| *S.maltophilia.CGMCC.1.1788GL001006* | 0.50 ± 0.13 |
| *S.maltophilia.CGMCC.1.1788GL001012* | 2.33 ± 0.62 |
| *S.maltophilia.CGMCC.1.1788GL001045* | 0.48 ± 0.02 |
| *S.maltophilia.CGMCC.1.1788GL001062* | 3.62 ± 1.10 |
| *S.maltophilia.CGMCC.1.1788GL001066* | 2.43 ± 0.22 |
| *S.maltophilia.CGMCC.1.1788GL001092* | 4.56 ± 1.87 |
| *S.maltophilia.CGMCC.1.1788GL001105* | 0.49 ± 0.04 |
| *S.maltophilia.CGMCC.1.1788GL001146* | 0.33 ± 0.15 |
| *S.maltophilia.CGMCC.1.1788GL001233* | 0.50 ± 0.01 |
| *S.maltophilia.CGMCC.1.1788GL001446* | 0.39 ± 0.18 |
| *S.maltophilia.CGMCC.1.1788GL001455* | 0.50 ± 0.03 |
| *S.maltophilia.CGMCC.1.1788GL001488* | 2.16 ± 0.52 |
| *S.maltophilia.CGMCC.1.1788GL001495* | 2.13 ± 0.26 |
| *S.maltophilia.CGMCC.1.1788GL001571* | 0.47 ± 0.03 |
| *S.maltophilia.CGMCC.1.1788GL001601* | 2.07 ± 0.04 |
| *S.maltophilia.CGMCC.1.1788GL001603* | 0.45 ± 0.02 |
| *S.maltophilia.CGMCC.1.1788GL001605* | 0.41 ± 0.03 |
| *S.maltophilia.CGMCC.1.1788GL001652* | 0.49 ± 0.10 |
| *S.maltophilia.CGMCC.1.1788GL001701* | 2.49 ± 1.18 |
| *S.maltophilia.CGMCC.1.1788GL001733* | 2.02 ± 0.17 |
| *S.maltophilia.CGMCC.1.1788GL001756* | 0.50 ± 0.09 |
| *S.maltophilia.CGMCC.1.1788GL001927* | 2.03 ± 0.05 |
| *S.maltophilia.CGMCC.1.1788GL001943* | 0.50 ± 0.01 |
| *S.maltophilia.CGMCC.1.1788GL001955* | 0.44 ± 0.01 |
| *S.maltophilia.CGMCC.1.1788GL001956* | 0.50 ± 0.14 |
| *S.maltophilia.CGMCC.1.1788GL002026* | 2.24 ± 0.53 |
| *S.maltophilia.CGMCC.1.1788GL002082* | 0.50 ± 0.05 |
| *S.maltophilia.CGMCC.1.1788GL002098* | 0.49 ± 0.00 |
| *S.maltophilia.CGMCC.1.1788GL002110* | 0.50 ± 0.18 |
| *S.maltophilia.CGMCC.1.1788GL002126* | 2.06 ± 0.33 |
| *S.maltophilia.CGMCC.1.1788GL002151* | 4.10 ± 0.84 |
| *S.maltophilia.CGMCC.1.1788GL002153* | 2.44 ± 0.02 |
| *S.maltophilia.CGMCC.1.1788GL002178* | 3.19 ± 0.49 |
| *S.maltophilia.CGMCC.1.1788GL002202* | 0.50 ± 0.13 |
| *S.maltophilia.CGMCC.1.1788GL002216* | 0.55 ± 0.15 |
| *S.maltophilia.CGMCC.1.1788GL002228* | 0.50 ± 0.01 |
| *S.maltophilia.CGMCC.1.1788GL002230* | 0.50 ± 0.03 |
| *S.maltophilia.CGMCC.1.1788GL002465* | 2.27 ± 0.12 |
| *S.maltophilia.CGMCC.1.1788GL002524* | 0.50 ± 0.01 |
| *S.maltophilia.CGMCC.1.1788GL002537* | 2.16 ± 0.07 |
| *S.maltophilia.CGMCC.1.1788GL002538* | 0.50 ± 0.01 |
| *S.maltophilia.CGMCC.1.1788GL002572* | 2.57 ± 0.83 |
| *S.maltophilia.CGMCC.1.1788GL002598* | 2.62 ± 0.26 |
| *S.maltophilia.CGMCC.1.1788GL002627* | 2.185 ± 0.29 |
| *S.maltophilia.CGMCC.1.1788GL002632* | 2.24 ± 0.36 |
| *S.maltophilia.CGMCC.1.1788GL002643* | 3.26 ± 1.19 |
| *S.maltophilia.CGMCC.1.1788GL002673* | 2.98 ± 0.17 |
| *S.maltophilia.CGMCC.1.1788GL002674* | 3.51 ± 0.63 |
| *S.maltophilia.CGMCC.1.1788GL002675* | 2.45 ± 0.73 |
| *S.maltophilia.CGMCC.1.1788GL002717* | 72.94 ± 20.18 |
| *S.maltophilia.CGMCC.1.1788GL002718* | 56.08 ± 26.11 |
| *S.maltophilia.CGMCC.1.1788GL002724* | 16.67 ± 6.09 |
| *S.maltophilia.CGMCC.1.1788GL002725* | 16.59 ± 6.35 |
| *S.maltophilia.CGMCC.1.1788GL002726* | 18.83 ± 6.72 |
| *S.maltophilia.CGMCC.1.1788GL002728* | 16.48 ± 5.39 |
| *S.maltophilia.CGMCC.1.1788GL002729* | 10.18 ± 2.62 |
| *S.maltophilia.CGMCC.1.1788GL002739* | 0.50 ± 0.31 |
| *S.maltophilia.CGMCC.1.1788GL002798* | 2.16 ± 0.16 |
| *S.maltophilia.CGMCC.1.1788GL002811* | 2.69 ± 0.03 |
| *S.maltophilia.CGMCC.1.1788GL002849* | 2.18 ± 0.37 |
| *S.maltophilia.CGMCC.1.1788GL002911* | 2.42 ± 0.91 |
| *S.maltophilia.CGMCC.1.1788GL002947* | 0.45 ± 0.10 |
| *S.maltophilia.CGMCC.1.1788GL003027* | 2.32 ± 0.67 |
| *S.maltophilia.CGMCC.1.1788GL003031* | 2.15 ± 0.32 |
| *S.maltophilia.CGMCC.1.1788GL003038* | 0.50 ± 0.05 |
| *S.maltophilia.CGMCC.1.1788GL003040* | 0.50 ± 0.04 |
| *S.maltophilia.CGMCC.1.1788GL003102* | 0.50 ± 0.16 |
| *S.maltophilia.CGMCC.1.1788GL003121* | 0.47 ± 0.02 |
| *S.maltophilia.CGMCC.1.1788GL003149* | 0.45 ± 0.02 |
| *S.maltophilia.CGMCC.1.1788GL003275* | 0.27 ± 0.08 |
| *S.maltophilia.CGMCC.1.1788GL003499* | 0.50 ± 0.03 |
| *S.maltophilia.CGMCC.1.1788GL003587* | 0.50 ± 0.12 |
| *S.maltophilia.CGMCC.1.1788GL003589* | 0.50 ± 0.03 |
| *S.maltophilia.CGMCC.1.1788GL003590* | 0.50 ± 0.10 |
| *S.maltophilia.CGMCC.1.1788GL003596* | 0.50 ± 0.30 |
| *S.maltophilia.CGMCC.1.1788GL003641* | 0.50 ± 0.14 |
| *S.maltophilia.CGMCC.1.1788GL003685* | 0.50 ± 0.14 |
| *S.maltophilia.CGMCC.1.1788GL003710* | 2.47 ± 0.56 |
| *S.maltophilia.CGMCC.1.1788GL003756* | 2.31 ± 0.52 |
| *S.maltophilia.CGMCC.1.1788GL003780* | 0.50 ± 0.14 |
| *S.maltophilia.CGMCC.1.1788GL003865* | 0.50 ± 0.02 |
| *S.maltophilia.CGMCC.1.1788GL003876* | 0.50 ± 0.11 |
| *S.maltophilia.CGMCC.1.1788GL003898* | 0.50 ± 0.07 |
| *S.maltophilia.CGMCC.1.1788GL003904* | 0.50 ± 0.09 |
| *S.maltophilia.CGMCC.1.1788GL003953* | 0.49 ± 0.02 |
| *S.maltophilia.CGMCC.1.1788GL003981* | 0.48 ± 0.08 |
| *S.maltophilia.CGMCC.1.1788GL004178* | 2.50 ± 0.46 |
| *S.maltophilia.CGMCC.1.1788GL004238* | 2.02 ± 0.79 |
| *S.maltophilia.CGMCC.1.1788GL004246* | 2.20 ± 0.75 |
| *S.maltophilia.CGMCC.1.1788GL004318* | 0.50 ± 0.08 |
| *S.maltophilia.CGMCC.1.1788GL004417* | 2.28 ± 0.08 |
| *S.maltophilia.CGMCC.1.1788GL004698* | 0.50 ± 0.05 |
| *S.maltophilia.CGMCC.1.1788GL004707* | 0.48 ± 0.05 |
| *novel0015* | 0.50 ± 0.15 |
| *novel0025* | 0.50 ± 0.14 |
| *novel0068* | 0.50 ± 0.01 |
| *novel0104* | 0.49 ± 0.01 |
| *novel0105* | 0.47 ± 0.00 |
| *novel0106* | 0.50 ± 0.20 |
| *novel0109* | 0.47 ± 0.03 |
| *novel0205* | 0.09 ± 0.06 |
| *novel0226* | 0.47 ± 0.15 |
| *novel0235* | 102.64 ± 42.38 |
| *novel0238* | 21.96 ± 9.56 |
| *novel0245* | 0.50 ± 0.27 |
| *novel0277* | 0.50 ± 0.07 |
| *novel0307* | 0.50 ± 0.16 |
| *novel0404* | 0.20± 0.00 |
| *sRNA0395* | 0.06 ± 0.05 |
| *sRNA0498* | 0.50 ± 0.23 |
