# Supplementary material for: Stenotrophomonas maltophilia uses a c-di-GMP module to sense the mammalian body temperature during infection
Source: PLoS Pathog. 2024 Sep 4;20(9):e1012533. doi: 10.1371/journal.ppat.1012533 (PMC11404848; doi:10.1371/journal.ppat.1012533)
Supplement: S2 Table — (DOCX) [file ppat.1012533.s011.docx]

| **S2 Table. Gene annotations by GO and KEGG.** | | |  |
| --- | --- | --- | --- |
| Go annotation | | |  |
| Gene_id | Go_term | GO_id |  |
| biological_process | biological regulation | GO:0065007 |  |
| *S.maltophilia.CGMCC.1.1788GL001955* | BP:regulation of transcription, DNA-templated;MF:DNA binding; | GO:0006355;GO:0003677 |  |
| *S.maltophilia.CGMCC.1.1788GL002717* | BP:regulation of transcription, DNA-templated; | GO:0006355 |  |
| *S.maltophilia.CGMCC.1.1788GL002572* | BP:regulation of transcription, DNA-templated;MF:sequence-specific DNA binding;MF:ATP binding; | GO:0006355;GO:0043565;GO:0005524 |  |
| *novel0277* | BP:mRNA catabolic process;BP:RNA processing;CC:cytoplasm;MF:magnesium ion binding;MF:polyribonucleotide nucleotidyltransferase activity;MF:RNA binding; | GO:0006402;GO:0006396;GO:0005737;GO:0000287;GO:0004654;GO:0003723 |  |
| *S.maltophilia.CGMCC.1.1788GL001733* | BP:phosphorelay signal transduction system;BP:regulation of transcription, DNA-templated;MF:DNA binding; | GO:0000160;GO:0006355;GO:0003677 |  |
| *S.maltophilia.CGMCC.1.1788GL000199* | BP:intracellular signal transduction;BP:cyclic nucleotide biosynthetic process;MF:adenylate cyclase activity; | GO:0035556;GO:0009190;GO:0004016 |  |
| *novel0156* | BP:cellular iron ion homeostasis;BP:iron ion transport;MF:ferric iron binding;MF:ferroxidase activity; | GO:0006879;GO:0006826;GO:0008199;GO:0004322 |  |
| *S.maltophilia.CGMCC.1.1788GL004052* | BP:regulation of cell shape;BP:cell morphogenesis;CC:cytoplasm;MF:ATP binding; | GO:0008360;GO:0000902;GO:0005737;GO:0005524 |  |
| *S.maltophilia.CGMCC.1.1788GL002026* | BP:negative regulation of transcription, DNA-templated;MF:DNA binding; | GO:0045892;GO:0003677 |  |
| *novel0235* | BP:regulation of transcription, DNA-templated; | GO:0006355 |  |
| *S.maltophilia.CGMCC.1.1788GL002324* | BP:phosphorelay signal transduction system;MF:DNA binding; | GO:0000160;GO:0003677 |  |
| *S.maltophilia.CGMCC.1.1788GL002811* | BP:regulation of transcription, DNA-templated;MF:DNA binding; | GO:0006355;GO:0003677 |  |
| *S.maltophilia.CGMCC.1.1788GL003588* | BP:regulation of transcription, DNA-templated; | GO:0006355 |  |
| biological_process | cellular process | GO:0009987 |  |
| *S.maltophilia.CGMCC.1.1788GL003134* | BP:generation of precursor metabolites and energy;MF:metal ion binding;MF:4 iron, 4 sulfur cluster binding;MF:fumarate hydratase activity; | GO:0006091;GO:0046872;GO:0051539;GO:0004333 |  |
| *S.maltophilia.CGMCC.1.1788GL002082* | BP:translation;CC:ribonucleoprotein complex;CC:ribosome;MF:structural constituent of ribosome;MF:RNA binding; | GO:0006412;GO:1990904;GO:0005840;GO:0003735;GO:0003723 |  |
| *S.maltophilia.CGMCC.1.1788GL002732* | BP:Mo-molybdopterin cofactor biosynthetic process;MF:nucleotidyltransferase activity;MF:catalytic activity;MF:GTP binding; | GO:0006777;GO:0016779;GO:0003824;GO:0005525 |  |
| *S.maltophilia.CGMCC.1.1788GL001601* | BP:phosphorylation;BP:'de novo' CTP biosynthetic process;CC:cytoplasm;MF:UMP kinase activity;MF:ATP binding; | GO:0016310;GO:0044210;GO:0005737;GO:0033862;GO:0005524 |  |
| *S.maltophilia.CGMCC.1.1788GL000393* | BP:heme O biosynthetic process;CC:integral component of membrane;CC:plasma membrane;MF:protoheme IX farnesyltransferase activity; | GO:0048034;GO:0016021;GO:0005886;GO:0008495 |  |
| *S.maltophilia.CGMCC.1.1788GL002729* | BP:nitrate metabolic process;CC:plasma membrane;CC:nitrate reductase complex;MF:nitrate reductase activity;MF:molybdopterin cofactor binding;MF:4 iron, 4 sulfur cluster binding;MF:metal ion binding; | GO:0042126;GO:0005886;GO:0009325;GO:0008940;GO:0043546;GO:0051539;GO:0046872 |  |
| *S.maltophilia.CGMCC.1.1788GL002728* | BP:nitrate metabolic process;CC:nitrate reductase complex;MF:nitrate reductase activity; | GO:0042126;GO:0009325;GO:0008940 |  |
| *S.maltophilia.CGMCC.1.1788GL002098* | BP:ribosome biogenesis;MF:GTP binding; | GO:0042254;GO:0005525 |  |
| *S.maltophilia.CGMCC.1.1788GL002726* | BP:nitrate assimilation;CC:nitrate reductase complex;CC:integral component of membrane;CC:plasma membrane;MF:nitrate reductase activity;MF:metal ion binding; | GO:0042128;GO:0009325;GO:0016021;GO:0005886;GO:0008940;GO:0046872 |  |
| *S.maltophilia.CGMCC.1.1788GL003625* | BP:protein folding;CC:cytoplasm;MF:unfolded protein binding; | GO:0006457;GO:0005737;GO:0051082 |  |
| *S.maltophilia.CGMCC.1.1788GL003158* | BP:enzyme-directed rRNA pseudouridine synthesis;MF:rRNA pseudouridine synthase activity;MF:lyase activity;MF:RNA binding;MF:hydrolase activity, acting on glycosyl bonds; | GO:0000455;GO:0120159;GO:0016829;GO:0003723;GO:0016798 |  |
| *S.maltophilia.CGMCC.1.1788GL000846* | BP:transcription, DNA-templated;CC:DNA-directed RNA polymerase complex;MF:magnesium ion binding;MF:zinc ion binding;MF:DNA binding;MF:DNA-directed RNA polymerase activity; | GO:0006351;GO:0000428;GO:0000287;GO:0008270;GO:0003677;GO:0003899 |  |
| *S.maltophilia.CGMCC.1.1788GL000843* | BP:translation;CC:ribonucleoprotein complex;CC:ribosome;MF:structural constituent of ribosome; | GO:0006412;GO:1990904;GO:0005840;GO:0003735 |  |
| *S.maltophilia.CGMCC.1.1788GL002718* | BP:cysteine biosynthetic process;MF:oxidoreductase activity;MF:FMN binding; | GO:0019344;GO:0016491;GO:0010181 |  |
| *S.maltophilia.CGMCC.1.1788GL001719* | BP:protein secretion by the type II secretion system;CC:integral component of membrane;CC:type II protein secretion system complex;CC:plasma membrane; | GO:0015628;GO:0016021;GO:0015627;GO:0005886 |  |
| *S.maltophilia.CGMCC.1.1788GL003898* | BP:valine biosynthetic process;BP:isoleucine biosynthetic process;MF:NADP binding;MF:isomerase activity;MF:magnesium ion binding;MF:ketol-acid reductoisomerase activity; | GO:0009099;GO:0009097;GO:0050661;GO:0016853;GO:0000287;GO:0004455 |  |
| *S.maltophilia.CGMCC.1.1788GL000876* | BP:translation;CC:small ribosomal subunit;MF:structural constituent of ribosome;MF:rRNA binding; | GO:0006412;GO:0015935;GO:0003735;GO:0019843 |  |
| *S.maltophilia.CGMCC.1.1788GL003169* | BP:cell division; | GO:0051301 |  |
| *novel0277* | S1 RNA-binding domain-containing protein, partial [Stenotrophomonas maltophilia] | GO:0006402;GO:0006396;GO:0005737;GO:0000287;GO:0004654;GO:0003723 |  |
| *S.maltophilia.CGMCC.1.1788GL000852* | BP:translation;CC:ribonucleoprotein complex;CC:ribosome;MF:structural constituent of ribosome;MF:tRNA binding; | GO:0006412;GO:1990904;GO:0005840;GO:0003735;GO:0000049 |  |
| *S.maltophilia.CGMCC.1.1788GL001721* | BP:type IV pilus biogenesis;CC:integral component of membrane; | GO:0043683;GO:0016021 |  |
| *S.maltophilia.CGMCC.1.1788GL000009* | BP:protein transport;CC:integral component of membrane;CC:plasma membrane; | GO:0015031;GO:0016021;GO:0005886 |  |
| *S.maltophilia.CGMCC.1.1788GL001488* | BP:protein transport;CC:cell outer membrane; | GO:0015031;GO:0009279 |  |
| *S.maltophilia.CGMCC.1.1788GL001735* | BP:establishment of competence for transformation;CC:integral component of membrane;CC:plasma membrane; | GO:0030420;GO:0016021;GO:0005886 |  |
| *S.maltophilia.CGMCC.1.1788GL001733* | BP:phosphorelay signal transduction system;BP:regulation of transcription, DNA-templated;MF:DNA binding; | GO:0000160;GO:0006355;GO:0003677 |  |
| *S.maltophilia.CGMCC.1.1788GL000199* | BP:intracellular signal transduction;BP:cyclic nucleotide biosynthetic process;MF:adenylate cyclase activity; | GO:0035556;GO:0009190;GO:0004016 |  |
| *S.maltophilia.CGMCC.1.1788GL000877* | BP:transcription, DNA-templated;CC:cytoplasm;CC:DNA-directed RNA polymerase complex;MF:DNA binding;MF:DNA-directed RNA polymerase activity;MF:protein dimerization activity; | GO:0006351;GO:0005737;GO:0000428;GO:0003677;GO:0003899;GO:0046983 |  |
| *S.maltophilia.CGMCC.1.1788GL000873* | BP:intracellular protein transmembrane transport;BP:protein targeting;BP:protein transport by the Sec complex;CC:integral component of membrane;CC:plasma membrane; | GO:0065002;GO:0006605;GO:0043952;GO:0016021;GO:0005886 |  |
| *S.maltophilia.CGMCC.1.1788GL001233* | BP:fatty acid biosynthetic process;BP:cellular carbohydrate metabolic process;MF:carboxy-lyase activity; | GO:0006633;GO:0044262;GO:0016831 |  |
| *S.maltophilia.CGMCC.1.1788GL004070* | BP:protein unfolding;BP:proteolysis;CC:HslUV protease complex;MF:proteasome-activating ATPase activity;MF:ATPase activity;MF:peptidase activity;MF:ATP binding; | GO:0043335;GO:0006508;GO:0009376;GO:0036402;GO:0016887;GO:0008233;GO:0005524 |  |
| *novel0156* | MULTISPECIES: bacterioferritin [Stenotrophomonas] | GO:0006879;GO:0006826;GO:0008199;GO:0004322 |  |
| *S.maltophilia.CGMCC.1.1788GL002113* | BP:glutamine metabolic process;MF:transferase activity; | GO:0006541;GO:0016740 |  |
| *S.maltophilia.CGMCC.1.1788GL002110* | BP:nucleoside metabolic process;MF:catalytic activity; | GO:0009116;GO:0003824 |  |
| *S.maltophilia.CGMCC.1.1788GL002324* | BP:phosphorelay signal transduction system;MF:DNA binding; | GO:0000160;GO:0003677 |  |
| *S.maltophilia.CGMCC.1.1788GL004178* | BP:DNA mediated transformation; | GO:0009294 |  |
| *S.maltophilia.CGMCC.1.1788GL001446* | BP:transposition, DNA-mediated;MF:transposase activity;MF:DNA binding; | GO:0006313;GO:0004803;GO:0003677 |  |
| *S.maltophilia.CGMCC.1.1788GL003685* | BP:cell division;BP:cell cycle;CC:integral component of membrane;CC:cell outer membrane; | GO:0051301;GO:0007049;GO:0016021;GO:0009279 |  |
| *S.maltophilia.CGMCC.1.1788GL003683* | BP:queuosine biosynthetic process;MF:magnesium ion binding;MF:S-adenosyl-L-methionine binding;MF:4 iron, 4 sulfur cluster binding;MF:carbon-nitrogen lyase activity; | GO:0008616;GO:0000287;GO:1904047;GO:0051539;GO:0016840 |  |
| *S.maltophilia.CGMCC.1.1788GL002724* | BP:nitrate assimilation;CC:integral component of membrane;MF:nitrate transmembrane transporter activity; | GO:0042128;GO:0016021;GO:0015112 |  |
| *S.maltophilia.CGMCC.1.1788GL004324* | BP:transmembrane transport;CC:integral component of plasma membrane; | GO:0055085;GO:0005887 |  |
| *S.maltophilia.CGMCC.1.1788GL004601* | BP:response to stimulus;BP:DNA-templated transcription, initiation;MF:sigma factor activity;MF:DNA binding; | GO:0050896;GO:0006352;GO:0016987;GO:0003677 |  |
| *S.maltophilia.CGMCC.1.1788GL001895* | BP:alanine biosynthetic process;BP:pantothenate biosynthetic process;CC:cytoplasm;MF:aspartate 1-decarboxylase activity; | GO:0006523;GO:0015940;GO:0005737;GO:0004068 |  |
| *S.maltophilia.CGMCC.1.1788GL002305* | BP:protein secretion;BP:bacterial-type flagellum assembly;CC:integral component of membrane;CC:plasma membrane; | GO:0009306;GO:0044780;GO:0016021;GO:0005886 |  |
| *S.maltophilia.CGMCC.1.1788GL002308* | BP:bacterial-type flagellum assembly;BP:protein targeting;CC:integral component of membrane;CC:plasma membrane;CC:bacterial-type flagellum basal body; | GO:0044780;GO:0006605;GO:0016021;GO:0005886;GO:0009425 |  |
| *S.maltophilia.CGMCC.1.1788GL001652* | BP:phosphate ion transmembrane transport;CC:ATP-binding cassett;MF:phosphate ion binding; | GO:0035435;GO:0043190;GO:0042301 |  |
| *S.maltophilia.CGMCC.1.1788GL002202* | BP:glycolytic process;MF:glucokinase activity;MF:ATP binding;MF:glucose binding; | GO:0006096;GO:0004340;GO:0005524;GO:0005536 |  |
| *novel0106* | BP:transcription, DNA-templated;CC:DNA-directed RNA polymerase complex;MF:magnesium ion binding;MF:zinc ion binding;MF:DNA binding;MF:DNA-directed RNA polymerase activity; | GO:0006351;GO:0000428;GO:0000287;GO:0008270;GO:0003677;GO:0003899 |  |
| *novel0104* | BP:transcription, DNA-templated;CC:DNA-directed RNA polymerase complex;MF:DNA-directed RNA polymerase activity;MF:ribonucleoside binding;MF:DNA binding; | GO:0006351;GO:0000428;GO:0003899;GO:0032549;GO:0003677 |  |
| *novel0109* | BP:transcription, DNA-templated;CC:DNA-directed RNA polymerase complex;MF:magnesium ion binding;MF:zinc ion binding;MF:DNA binding;MF:DNA-directed RNA polymerase activity; | GO:0006351;GO:0000428;GO:0000287;GO:0008270;GO:0003677;GO:0003899 |  |
| *S.maltophilia.CGMCC.1.1788GL004238* | BP:chorismate biosynthetic process;BP:aromatic amino acid family biosynthetic process;BP:cellular amino acid biosynthetic process;MF:3-dehydroquinate dehydratase activity; | GO:0009423;GO:0009073;GO:0008652;GO:0003855 |  |
| *S.maltophilia.CGMCC.1.1788GL002671* | BP:DNA-templated transcription, initiation;MF:sigma factor activity;MF:DNA binding; | GO:0006352;GO:0016987;GO:0003677 |  |
| *S.maltophilia.CGMCC.1.1788GL002046* | BP:queuosine biosynthetic process;CC:cytoplasm;MF:S-adenosylmethionin;MF:transferase activity;MF:isomerase activity; | GO:0008616;GO:0005737;GO:0051075;GO:0016740;GO:0016853 |  |
| *S.maltophilia.CGMCC.1.1788GL002198* | BP:histidine biosynthetic process;CC:cytoplasm;MF:imidazoleglycerol-phosphate synthase activity;MF:lyase activity; | GO:0000105;GO:0005737;GO:0000107;GO:0016829 |  |
| *S.maltophilia.CGMCC.1.1788GL002196* | BP:glutamine metabolic process;BP:histidine biosynthetic process;CC:cytoplasm;MF:hydrolase activity;MF:glutaminase activity;MF:lyase activity;MF:imidazoleglycerol-phosphate synthase activity; | GO:0006541;GO:0000105;GO:0005737;GO:0016787;GO:0004359;GO:0016829;GO:0000107 |  |
| *S.maltophilia.CGMCC.1.1788GL002197* | BP:histidine biosynthetic process;CC:cytoplasm;MF:1; | GO:0000105;GO:0005737;GO:0003949 |  |
| *S.maltophilia.CGMCC.1.1788GL002194* | BP:histidine biosynthetic process;MF:pyridoxal phosphate binding;MF:histidinol-phosphate transaminase activity; | GO:0000105;GO:0030170;GO:0004400 |  |
| *S.maltophilia.CGMCC.1.1788GL002195* | BP:histidine biosynthetic process;CC:cytoplasm;MF:metal ion binding;MF:imidazoleglycerol-phosphate dehydratase activity;MF:histidinol-phosphatase activity; | GO:0000105;GO:0005737;GO:0046872;GO:0004424;GO:0004401 |  |
| *S.maltophilia.CGMCC.1.1788GL003876* | BP:protein transport;CC:integral component of membrane;CC:plasma membrane;MF:transmembrane transporter activity; | GO:0015031;GO:0016021;GO:0005886;GO:0022857 |  |
| *S.maltophilia.CGMCC.1.1788GL003875* | BP:protein transport;CC:integral component of membrane;CC:plasma membrane;MF:transmembrane transporter activity; | GO:0015031;GO:0016021;GO:0005886;GO:0022857 |  |
| *S.maltophilia.CGMCC.1.1788GL003904* | BP:leucine biosynthetic process;MF:2-isopropylmalate synthase activity; | GO:0009098;GO:0003852 |  |
| *S.maltophilia.CGMCC.1.1788GL003589* | BP:propionate metabolic process, methylcitrate cycle;MF:citrate dehydratase activity;MF:aconitate hydratase activity;MF:iron-sulfur cluster binding;MF:metal ion binding; | GO:0019679;GO:0047780;GO:0003994;GO:0051536;GO:0046872 |  |
| *novel0307* | MULTISPECIES: peptidoglycan-associated lipoprotein Pal [Stenotrophomonas] | GO:0051301;GO:0007049;GO:0016021;GO:0009279 |  |
| *S.maltophilia.CGMCC.1.1788GL002185* | BP:phosphorylation;BP:threonine biosynthetic process;CC:cytoplasm;MF:homoserine kinase activity;MF:ATP binding; | GO:0016310;GO:0009088;GO:0005737;GO:0004413;GO:0005524 |  |
| *S.maltophilia.CGMCC.1.1788GL004249* | BP:protein dephosphorylation;CC:integral component of membrane;MF:protein tyrosine/serine/threonine phosphatase activity; | GO:0006470;GO:0016021;GO:0008138 |  |
| biological_process | developmental process | GO:0032502 |  |
| *S.maltophilia.CGMCC.1.1788GL004052* | BP:regulation of cell shape;BP:cell morphogenesis;CC:cytoplasm;MF:ATP binding; | GO:0008360;GO:0000902;GO:0005737;GO:0005524 |  |
| biological_process | localization | GO:0051179 |  |
| *S.maltophilia.CGMCC.1.1788GL000534* | BP:ion transport;CC:cell outer membrane;CC:pore complex;MF:porin activity;MF:cobalamin-transporting ATPase activity; | GO:0006811;GO:0009279;GO:0046930;GO:0015288;GO:0015420 |  |
| *S.maltophilia.CGMCC.1.1788GL001719* | BP:protein secretion by the type II secretion system;CC:integral component of membrane;CC:type II protein secretion system complex;CC:plasma membrane; | GO:0015628;GO:0016021;GO:0015627;GO:0005886 |  |
| *S.maltophilia.CGMCC.1.1788GL000009* | BP:protein transport;CC:integral component of membrane;CC:plasma membrane; | GO:0015031;GO:0016021;GO:0005886 |  |
| *S.maltophilia.CGMCC.1.1788GL001488* | BP:protein transport;CC:cell outer membrane; | GO:0015031;GO:0009279 |  |
| *S.maltophilia.CGMCC.1.1788GL000873* | BP:intracellular protein transmembrane transport;BP:protein targeting;BP:protein transport by the Sec complex;CC:integral component of membrane;CC:plasma membrane; | GO:0065002;GO:0006605;GO:0043952;GO:0016021;GO:0005886 |  |
| *novel0156* | BP:cellular iron ion homeostasis;BP:iron ion transport;MF:ferric iron binding;MF:ferroxidase activity; | GO:0006879;GO:0006826;GO:0008199;GO:0004322 |  |
| *S.maltophilia.CGMCC.1.1788GL004324* | BP:transmembrane transport;CC:integral component of plasma membrane; | GO:0055085;GO:0005887 |  |
| *S.maltophilia.CGMCC.1.1788GL002305* | BP:protein secretion;BP:bacterial-type flagellum assembly;CC:integral component of membrane;CC:plasma membrane; | GO:0009306;GO:0044780;GO:0016021;GO:0005886 |  |
| *S.maltophilia.CGMCC.1.1788GL002308* | BP:bacterial-type flagellum assembly;BP:protein targeting;CC:integral component of membrane;CC:plasma membrane;CC:bacterial-type flagellum basal body; | GO:0044780;GO:0006605;GO:0016021;GO:0005886;GO:0009425 |  |
| *S.maltophilia.CGMCC.1.1788GL001652* | BP:phosphate ion transmembrane transport;CC:ATP-binding cassett;MF:phosphate ion binding; | GO:0035435;GO:0043190;GO:0042301 |  |
| *S.maltophilia.CGMCC.1.1788GL002206* | BP:copper ion transport;CC:integral component of membrane;CC:plasma membrane;MF:cation-transporting ATPase activity;MF:ATPase activity;MF:copper ion binding;MF:ATP binding; | GO:0006825;GO:0016021;GO:0005886;GO:0019829;GO:0016887;GO:0005507;GO:0005524 |  |
| *S.maltophilia.CGMCC.1.1788GL003876* | BP:protein transport;CC:integral component of membrane;CC:plasma membrane;MF:transmembrane transporter activity; | GO:0015031;GO:0016021;GO:0005886;GO:0022857 |  |
| *S.maltophilia.CGMCC.1.1788GL003875* | BP:protein transport;CC:integral component of membrane;CC:plasma membrane;MF:transmembrane transporter activity; | GO:0015031;GO:0016021;GO:0005886;GO:0022857 |  |
| biological_process | metabolic process | GO:0008152 |  |
| *S.maltophilia.CGMCC.1.1788GL003134* | BP:generation of precursor metabolites and energy;MF:metal ion binding;MF:4 iron, 4 sulfur cluster binding;MF:fumarate hydratase activity; | GO:0006091;GO:0046872;GO:0051539;GO:0004333 |  |
| *S.maltophilia.CGMCC.1.1788GL002082* | BP:translation;CC:ribonucleoprotein complex;CC:ribosome;MF:structural constituent of ribosome;MF:RNA binding; | GO:0006412;GO:1990904;GO:0005840;GO:0003735;GO:0003723 |  |
| *S.maltophilia.CGMCC.1.1788GL002732* | BP:Mo-molybdopterin cofactor biosynthetic process;MF:nucleotidyltransferase activity;MF:catalytic activity;MF:GTP binding; | GO:0006777;GO:0016779;GO:0003824;GO:0005525 |  |
| *S.maltophilia.CGMCC.1.1788GL001601* | BP:phosphorylation;BP:'de novo' CTP biosynthetic process;CC:cytoplasm;MF:UMP kinase activity;MF:ATP binding; | GO:0016310;GO:0044210;GO:0005737;GO:0033862;GO:0005524 |  |
| *S.maltophilia.CGMCC.1.1788GL000393* | BP:heme O biosynthetic process;CC:integral component of membrane;CC:plasma membrane;MF:protoheme IX farnesyltransferase activity; | GO:0048034;GO:0016021;GO:0005886;GO:0008495 |  |
| *S.maltophilia.CGMCC.1.1788GL002729* | BP:nitrate metabolic process;CC:plasma membrane;CC:nitrate reductase complex;MF:nitrate reductase activity;MF:molybdopterin cofactor binding;MF:4 iron, 4 sulfur cluster binding;MF:metal ion binding; | GO:0042126;GO:0005886;GO:0009325;GO:0008940;GO:0043546;GO:0051539;GO:0046872 |  |
| *S.maltophilia.CGMCC.1.1788GL002728* | BP:nitrate metabolic process;CC:nitrate reductase complex;MF:nitrate reductase activity; | GO:0042126;GO:0009325;GO:0008940 |  |
| *S.maltophilia.CGMCC.1.1788GL002726* | BP:nitrate assimilation;CC:nitrate reductase complex;CC:integral component of membrane;CC:plasma membrane;MF:nitrate reductase activity;MF:metal ion binding; | GO:0042128;GO:0009325;GO:0016021;GO:0005886;GO:0008940;GO:0046872 |  |
| *S.maltophilia.CGMCC.1.1788GL001701* | BP:peptidoglycan metabolic process;CC:membrane;MF:lytic transglycosylase activity; | GO:0000270;GO:0016020;GO:0008933 |  |
| *S.maltophilia.CGMCC.1.1788GL003158* | BP:enzyme-directed rRNA pseudouridine synthesis;MF:rRNA pseudouridine synthase activity;MF:lyase activity;MF:RNA binding;MF:hydrolase activity, acting on glycosyl bonds; | GO:0000455;GO:0120159;GO:0016829;GO:0003723;GO:0016798 |  |
| *S.maltophilia.CGMCC.1.1788GL000846* | BP:transcription, DNA-templated;CC:DNA-directed RNA polymerase complex;MF:magnesium ion binding;MF:zinc ion binding;MF:DNA binding;MF:DNA-directed RNA polymerase activity; | GO:0006351;GO:0000428;GO:0000287;GO:0008270;GO:0003677;GO:0003899 |  |
| *S.maltophilia.CGMCC.1.1788GL000843* | BP:translation;CC:ribonucleoprotein complex;CC:ribosome;MF:structural constituent of ribosome; | GO:0006412;GO:1990904;GO:0005840;GO:0003735 |  |
| *S.maltophilia.CGMCC.1.1788GL002718* | BP:cysteine biosynthetic process;MF:oxidoreductase activity;MF:FMN binding; | GO:0019344;GO:0016491;GO:0010181 |  |
| *S.maltophilia.CGMCC.1.1788GL003898* | BP:valine biosynthetic process;BP:isoleucine biosynthetic process;MF:NADP binding;MF:isomerase activity;MF:magnesium ion binding;MF:ketol-acid reductoisomerase activity; | GO:0009099;GO:0009097;GO:0050661;GO:0016853;GO:0000287;GO:0004455 |  |
| *S.maltophilia.CGMCC.1.1788GL000876* | BP:translation;CC:small ribosomal subunit;MF:structural constituent of ribosome;MF:rRNA binding; | GO:0006412;GO:0015935;GO:0003735;GO:0019843 |  |
| *novel0277* | S1 RNA-binding domain-containing protein, partial [Stenotrophomonas maltophilia] | GO:0006402;GO:0006396;GO:0005737;GO:0000287;GO:0004654;GO:0003723 |  |
| *S.maltophilia.CGMCC.1.1788GL000852* | BP:translation;CC:ribonucleoprotein complex;CC:ribosome;MF:structural constituent of ribosome;MF:tRNA binding; | GO:0006412;GO:1990904;GO:0005840;GO:0003735;GO:0000049 |  |
| *S.maltophilia.CGMCC.1.1788GL001495* | BP:nitrogen compound metabolic process;BP:primary metabolic process;BP:macromolecule metabolic process;MF:catalytic activity; | GO:0006807;GO:0044238;GO:0043170;GO:0003824 |  |
| *S.maltophilia.CGMCC.1.1788GL003953* | BP:carbohydrate metabolic process;MF:hydrolase activity, acting on carbon-nitroge; | GO:0005975;GO:0016810 |  |
| *S.maltophilia.CGMCC.1.1788GL000199* | BP:intracellular signal transduction;BP:cyclic nucleotide biosynthetic process;MF:adenylate cyclase activity; | GO:0035556;GO:0009190;GO:0004016 |  |
| *S.maltophilia.CGMCC.1.1788GL000877* | BP:transcription, DNA-templated;CC:cytoplasm;CC:DNA-directed RNA polymerase complex;MF:DNA binding;MF:DNA-directed RNA polymerase activity;MF:protein dimerization activity; | GO:0006351;GO:0005737;GO:0000428;GO:0003677;GO:0003899;GO:0046983 |  |
| *S.maltophilia.CGMCC.1.1788GL000724* | BP:proteolysis;MF:serine-type peptidase activity; | GO:0006508;GO:0008236 |  |
| *S.maltophilia.CGMCC.1.1788GL001233* | BP:fatty acid biosynthetic process;BP:cellular carbohydrate metabolic process;MF:carboxy-lyase activity; | GO:0006633;GO:0044262;GO:0016831 |  |
| *S.maltophilia.CGMCC.1.1788GL000500* | BP:proteolysis;MF:metal ion binding;MF:metalloendopeptidase activity; | GO:0006508;GO:0046872;GO:0004222 |  |
| *S.maltophilia.CGMCC.1.1788GL004070* | BP:protein unfolding;BP:proteolysis;CC:HslUV protease complex;MF:proteasome-activating ATPase activity;MF:ATPase activity;MF:peptidase activity;MF:ATP binding; | GO:0043335;GO:0006508;GO:0009376;GO:0036402;GO:0016887;GO:0008233;GO:0005524 |  |
| *S.maltophilia.CGMCC.1.1788GL002113* | BP:glutamine metabolic process;MF:transferase activity; | GO:0006541;GO:0016740 |  |
| *S.maltophilia.CGMCC.1.1788GL002110* | BP:nucleoside metabolic process;MF:catalytic activity; | GO:0009116;GO:0003824 |  |
| *S.maltophilia.CGMCC.1.1788GL003354* | BP:nitrogen compound metabolic process;MF:amino-acid racemase activity; | GO:0006807;GO:0047661 |  |
| *S.maltophilia.CGMCC.1.1788GL001446* | BP:transposition, DNA-mediated;MF:transposase activity;MF:DNA binding; | GO:0006313;GO:0004803;GO:0003677 |  |
| *S.maltophilia.CGMCC.1.1788GL003683* | BP:queuosine biosynthetic process;MF:magnesium ion binding;MF:S-adenosyl-L-methionine binding;MF:4 iron, 4 sulfur cluster binding;MF:carbon-nitrogen lyase activity; | GO:0008616;GO:0000287;GO:1904047;GO:0051539;GO:0016840 |  |
| *S.maltophilia.CGMCC.1.1788GL002724* | BP:nitrate assimilation;CC:integral component of membrane;MF:nitrate transmembrane transporter activity; | GO:0042128;GO:0016021;GO:0015112 |  |
| *S.maltophilia.CGMCC.1.1788GL004090* | BP:proteolysis;MF:metal ion binding;MF:metalloendopeptidase activity; | GO:0006508;GO:0046872;GO:0004222 |  |
| *S.maltophilia.CGMCC.1.1788GL004601* | BP:response to stimulus;BP:DNA-templated transcription, initiation;MF:sigma factor activity;MF:DNA binding; | GO:0050896;GO:0006352;GO:0016987;GO:0003677 |  |
| *S.maltophilia.CGMCC.1.1788GL001895* | BP:alanine biosynthetic process;BP:pantothenate biosynthetic process;CC:cytoplasm;MF:aspartate 1-decarboxylase activity; | GO:0006523;GO:0015940;GO:0005737;GO:0004068 |  |
| *novel0025* | MULTISPECIES: isovaleryl-CoA dehydrogenase [Stenotrophomonas] | GO:1901565;GO:0003995;GO:0050660 |  |
| *S.maltophilia.CGMCC.1.1788GL002202* | BP:glycolytic process;MF:glucokinase activity;MF:ATP binding;MF:glucose binding; | GO:0006096;GO:0004340;GO:0005524;GO:0005536 |  |
| *S.maltophilia.CGMCC.1.1788GL000224* | BP:lipid catabolic process;MF:hydrolase activity; | GO:0016042;GO:0016787 |  |
| *novel0106* | hypothetical protein, partial [Corallococcus sp. AB049A] | GO:0006351;GO:0000428;GO:0000287;GO:0008270;GO:0003677;GO:0003899 |  |
| *novel0104* | DNA-directed RNA polymerase subunit beta [Stenotrophomonas maltophilia] | GO:0006351;GO:0000428;GO:0003899;GO:0032549;GO:0003677 |  |
| *novel0109* | hypothetical protein, partial [Enterobacter hormaechei] | GO:0006351;GO:0000428;GO:0000287;GO:0008270;GO:0003677;GO:0003899 |  |
| *S.maltophilia.CGMCC.1.1788GL004238* | BP:chorismate biosynthetic process;BP:aromatic amino acid family biosynthetic process;BP:cellular amino acid biosynthetic process;MF:3-dehydroquinate dehydratase activity; | GO:0009423;GO:0009073;GO:0008652;GO:0003855 |  |
| *S.maltophilia.CGMCC.1.1788GL002671* | BP:DNA-templated transcription, initiation;MF:sigma factor activity;MF:DNA binding; | GO:0006352;GO:0016987;GO:0003677 |  |
| *S.maltophilia.CGMCC.1.1788GL002216* | BP:carbohydrate metabolic process;MF:hydrolase activity, hydrolyzing O-glycosyl compounds; | GO:0005975;GO:0004553 |  |
| *S.maltophilia.CGMCC.1.1788GL002046* | BP:queuosine biosynthetic process;CC:cytoplasm;MF:S-adenosylmethionin;MF:transferase activity;MF:isomerase activity; | GO:0008616;GO:0005737;GO:0051075;GO:0016740;GO:0016853 |  |
| *S.maltophilia.CGMCC.1.1788GL002198* | BP:histidine biosynthetic process;CC:cytoplasm;MF:imidazoleglycerol-phosphate synthase activity;MF:lyase activity; | GO:0000105;GO:0005737;GO:0000107;GO:0016829 |  |
| *S.maltophilia.CGMCC.1.1788GL002196* | BP:glutamine metabolic process;BP:histidine biosynthetic process;CC:cytoplasm;MF:hydrolase activity;MF:glutaminase activity;MF:lyase activity;MF:imidazoleglycerol-phosphate synthase activity; | GO:0006541;GO:0000105;GO:0005737;GO:0016787;GO:0004359;GO:0016829;GO:0000107 |  |
| *S.maltophilia.CGMCC.1.1788GL002197* | BP:histidine biosynthetic process;CC:cytoplasm;MF:1; | GO:0000105;GO:0005737;GO:0003949 |  |
| *S.maltophilia.CGMCC.1.1788GL002194* | BP:histidine biosynthetic process;MF:pyridoxal phosphate binding;MF:histidinol-phosphate transaminase activity; | GO:0000105;GO:0030170;GO:0004400 |  |
| *S.maltophilia.CGMCC.1.1788GL002195* | BP:histidine biosynthetic process;CC:cytoplasm;MF:metal ion binding;MF:imidazoleglycerol-phosphate dehydratase activity;MF:histidinol-phosphatase activity; | GO:0000105;GO:0005737;GO:0046872;GO:0004424;GO:0004401 |  |
| *S.maltophilia.CGMCC.1.1788GL002538* | BP:proteolysis;MF:serine-type endopeptidase activity; | GO:0006508;GO:0004252 |  |
| *S.maltophilia.CGMCC.1.1788GL003904* | BP:leucine biosynthetic process;MF:2-isopropylmalate synthase activity; | GO:0009098;GO:0003852 |  |
| *S.maltophilia.CGMCC.1.1788GL003589* | BP:propionate metabolic process, methylcitrate cycle;MF:citrate dehydratase activity;MF:aconitate hydratase activity;MF:iron-sulfur cluster binding;MF:metal ion binding; | GO:0019679;GO:0047780;GO:0003994;GO:0051536;GO:0046872 |  |
| *S.maltophilia.CGMCC.1.1788GL002185* | BP:phosphorylation;BP:threonine biosynthetic process;CC:cytoplasm;MF:homoserine kinase activity;MF:ATP binding; | GO:0016310;GO:0009088;GO:0005737;GO:0004413;GO:0005524 |  |
| *S.maltophilia.CGMCC.1.1788GL004249* | BP:protein dephosphorylation;CC:integral component of membrane;MF:protein tyrosine/serine/threonine phosphatase activity; | GO:0006470;GO:0016021;GO:0008138 |  |
| *S.maltophilia.CGMCC.1.1788GL003590* | BP:tricarboxylic acid cycle;CC:cytoplasm;MF:transferase activity, transferring acyl groups, acyl groups converted into alkyl on transfer;MF:citrate synthase activity; | GO:0006099;GO:0005737;GO:0046912;GO:0036440 |  |
| *S.maltophilia.CGMCC.1.1788GL003040* | BP:proteolysis;MF:serine-type peptidase activity; | GO:0006508;GO:0008236 |  |
| biological_process | response to stimulus | GO:0050896 |  |
| *S.maltophilia.CGMCC.1.1788GL001735* | BP:establishment of competence for transformation;CC:integral component of membrane;CC:plasma membrane; | GO:0030420;GO:0016021;GO:0005886 |  |
| *S.maltophilia.CGMCC.1.1788GL004698* | BP:response to oxidative stress;MF:glutathione peroxidase activity; | GO:0006979;GO:0004602 |  |
| *S.maltophilia.CGMCC.1.1788GL004601* | BP:response to stimulus;BP:DNA-templated transcription, initiation;MF:sigma factor activity;MF:DNA binding; | GO:0050896;GO:0006352;GO:0016987;GO:0003677 |  |
| *S.maltophilia.CGMCC.1.1788GL001012* | BP:response to chemical;CC:integral component of plasma membrane;MF:transmembrane transporter activity; | GO:0042221;GO:0005887;GO:0022857 |  |
| *S.maltophilia.CGMCC.1.1788GL003596* | BP:response to antibiotic;MF:aminoglycoside 6'-N-acetyltransferase activity; | GO:0046677;GO:0047663 |  |
| biological_process | viral process | GO:0016032 |  |
| *S.maltophilia.CGMCC.1.1788GL000280* | BP:viral capsid assembly;MF:ATP binding; | GO:0019069;GO:0005524 |  |
| cellular_component | cellular anatomical entity | GO:0110165 |  |
| *S.maltophilia.CGMCC.1.1788GL002082* | BP:translation;CC:ribonucleoprotein complex;CC:ribosome;MF:structural constituent of ribosome;MF:RNA binding; | GO:0006412;GO:1990904;GO:0005840;GO:0003735;GO:0003723 |  |
| *S.maltophilia.CGMCC.1.1788GL001513* | CC:integral component of membrane; | GO:0016021 |  |
| *S.maltophilia.CGMCC.1.1788GL001601* | BP:phosphorylation;BP:'de novo' CTP biosynthetic process;CC:cytoplasm;MF:UMP kinase activity;MF:ATP binding; | GO:0016310;GO:0044210;GO:0005737;GO:0033862;GO:0005524 |  |
| *S.maltophilia.CGMCC.1.1788GL000393* | BP:heme O biosynthetic process;CC:integral component of membrane;CC:plasma membrane;MF:protoheme IX farnesyltransferase activity; | GO:0048034;GO:0016021;GO:0005886;GO:0008495 |  |
| *S.maltophilia.CGMCC.1.1788GL001943* | CC:integral component of membrane; | GO:0016021 |  |
| *S.maltophilia.CGMCC.1.1788GL000834* | CC:cell outer membrane; | GO:0009279 |  |
| *S.maltophilia.CGMCC.1.1788GL004318* | CC:integral component of membrane;CC:plasma membrane; | GO:0016021;GO:0005886 |  |
| *S.maltophilia.CGMCC.1.1788GL002729* | BP:nitrate metabolic process;CC:plasma membrane;CC:nitrate reductase complex;MF:nitrate reductase activity;MF:molybdopterin cofactor binding;MF:4 iron, 4 sulfur cluster binding;MF:metal ion binding; | GO:0042126;GO:0005886;GO:0009325;GO:0008940;GO:0043546;GO:0051539;GO:0046872 |  |
| *S.maltophilia.CGMCC.1.1788GL002726* | BP:nitrate assimilation;CC:nitrate reductase complex;CC:integral component of membrane;CC:plasma membrane;MF:nitrate reductase activity;MF:metal ion binding; | GO:0042128;GO:0009325;GO:0016021;GO:0005886;GO:0008940;GO:0046872 |  |
| *S.maltophilia.CGMCC.1.1788GL002947* | CC:integral component of membrane; | GO:0016021 |  |
| *S.maltophilia.CGMCC.1.1788GL001701* | BP:peptidoglycan metabolic process;CC:membrane;MF:lytic transglycosylase activity; | GO:0000270;GO:0016020;GO:0008933 |  |
| *S.maltophilia.CGMCC.1.1788GL000534* | BP:ion transport;CC:cell outer membrane;CC:pore complex;MF:porin activity;MF:cobalamin-transporting ATPase activity; | GO:0006811;GO:0009279;GO:0046930;GO:0015288;GO:0015420 |  |
| *S.maltophilia.CGMCC.1.1788GL003625* | BP:protein folding;CC:cytoplasm;MF:unfolded protein binding; | GO:0006457;GO:0005737;GO:0051082 |  |
| *S.maltophilia.CGMCC.1.1788GL002739* | CC:integral component of membrane; | GO:0016021 |  |
| *S.maltophilia.CGMCC.1.1788GL004532* | CC:integral component of membrane;CC:plasma membrane; | GO:0016021;GO:0005886 |  |
| *S.maltophilia.CGMCC.1.1788GL002151* | CC:integral component of membrane; | GO:0016021 |  |
| *S.maltophilia.CGMCC.1.1788GL000843* | BP:translation;CC:ribonucleoprotein complex;CC:ribosome;MF:structural constituent of ribosome; | GO:0006412;GO:1990904;GO:0005840;GO:0003735 |  |
| *S.maltophilia.CGMCC.1.1788GL001603* | CC:cytoplasm;MF:translation elongation factor activity; | GO:0005737;GO:0003746 |  |
| *S.maltophilia.CGMCC.1.1788GL000711* | CC:integral component of membrane;MF:hydrolase activity; | GO:0016021;GO:0016787 |  |
| *S.maltophilia.CGMCC.1.1788GL000159* | CC:outer membrane; | GO:0019867 |  |
| *S.maltophilia.CGMCC.1.1788GL001719* | BP:protein secretion by the type II secretion system;CC:integral component of membrane;CC:type II protein secretion system complex;CC:plasma membrane; | GO:0015628;GO:0016021;GO:0015627;GO:0005886 |  |
| *S.maltophilia.CGMCC.1.1788GL001006* | CC:outer membrane; | GO:0019867 |  |
| *S.maltophilia.CGMCC.1.1788GL004707* | CC:integral component of membrane;MF:transmembrane transporter activity; | GO:0016021;GO:0022857 |  |
| *novel0277* | S1 RNA-binding domain-containing protein, partial [Stenotrophomonas maltophilia] | GO:0006402;GO:0006396;GO:0005737;GO:0000287;GO:0004654;GO:0003723 |  |
| *S.maltophilia.CGMCC.1.1788GL004100* | CC:integral component of membrane;CC:plasma membrane; | GO:0016021;GO:0005886 |  |
| *S.maltophilia.CGMCC.1.1788GL000852* | BP:translation;CC:ribonucleoprotein complex;CC:ribosome;MF:structural constituent of ribosome;MF:tRNA binding; | GO:0006412;GO:1990904;GO:0005840;GO:0003735;GO:0000049 |  |
| *S.maltophilia.CGMCC.1.1788GL001146* | CC:integral component of membrane; | GO:0016021 |  |
| *S.maltophilia.CGMCC.1.1788GL001721* | BP:type IV pilus biogenesis;CC:integral component of membrane; | GO:0043683;GO:0016021 |  |
| *S.maltophilia.CGMCC.1.1788GL004286* | CC:integral component of membrane; | GO:0016021 |  |
| *S.maltophilia.CGMCC.1.1788GL000805* | CC:integral component of membrane; | GO:0016021 |  |
| *S.maltophilia.CGMCC.1.1788GL000009* | BP:protein transport;CC:integral component of membrane;CC:plasma membrane; | GO:0015031;GO:0016021;GO:0005886 |  |
| *S.maltophilia.CGMCC.1.1788GL003554* | CC:integral component of membrane;CC:plasma membrane;MF:transferase activity, transferring phosphorus-containing groups;MF:sulfuric ester hydrolase activity; | GO:0016021;GO:0005886;GO:0016772;GO:0008484 |  |
| *S.maltophilia.CGMCC.1.1788GL003370* | CC:integral component of membrane; | GO:0016021 |  |
| *novel0192* | hypothetical protein [Stenotrophomonas maltophilia] | GO:0016021 |  |
| *S.maltophilia.CGMCC.1.1788GL002772* | CC:integral component of membrane;CC:plasma membrane; | GO:0016021;GO:0005886 |  |
| *S.maltophilia.CGMCC.1.1788GL001488* | BP:protein transport;CC:cell outer membrane; | GO:0015031;GO:0009279 |  |
| *S.maltophilia.CGMCC.1.1788GL001735* | BP:establishment of competence for transformation;CC:integral component of membrane;CC:plasma membrane; | GO:0030420;GO:0016021;GO:0005886 |  |
| *S.maltophilia.CGMCC.1.1788GL000192* | CC:integral component of membrane;MF:transmembrane transporter activity; | GO:0016021;GO:0022857 |  |
| *S.maltophilia.CGMCC.1.1788GL003968* | CC:integral component of membrane; | GO:0016021 |  |
| *S.maltophilia.CGMCC.1.1788GL000877* | BP:transcription, DNA-templated;CC:cytoplasm;CC:DNA-directed RNA polymerase complex;MF:DNA binding;MF:DNA-directed RNA polymerase activity;MF:protein dimerization activity; | GO:0006351;GO:0005737;GO:0000428;GO:0003677;GO:0003899;GO:0046983 |  |
| *S.maltophilia.CGMCC.1.1788GL000873* | BP:intracellular protein transmembrane transport;BP:protein targeting;BP:protein transport by the Sec complex;CC:integral component of membrane;CC:plasma membrane; | GO:0065002;GO:0006605;GO:0043952;GO:0016021;GO:0005886 |  |
| *S.maltophilia.CGMCC.1.1788GL001822* | CC:cytoplasm;MF:metal ion binding;MF:GTPase activity;MF:GTP binding; | GO:0005737;GO:0046872;GO:0003924;GO:0005525 |  |
| *S.maltophilia.CGMCC.1.1788GL004052* | BP:regulation of cell shape;BP:cell morphogenesis;CC:cytoplasm;MF:ATP binding; | GO:0008360;GO:0000902;GO:0005737;GO:0005524 |  |
| *novel0083* | hypothetical protein, partial [Mesorhizobium sp. M8A.F.Ca.ET.161.01.1.1] | GO:0016021;GO:0016787 |  |
| *S.maltophilia.CGMCC.1.1788GL000662* | CC:integral component of membrane; | GO:0016021 |  |
| *S.maltophilia.CGMCC.1.1788GL001045* | CC:integral component of membrane; | GO:0016021 |  |
| *S.maltophilia.CGMCC.1.1788GL003038* | CC:integral component of membrane; | GO:0016021 |  |
| *S.maltophilia.CGMCC.1.1788GL002037* | CC:integral component of membrane;MF:ABC-type transmembrane transporter activity;MF:ATP binding; | GO:0016021;GO:0140359;GO:0005524 |  |
| *S.maltophilia.CGMCC.1.1788GL000416* | CC:cell outer membrane;MF:carbohydrate binding; | GO:0009279;GO:0030246 |  |
| *S.maltophilia.CGMCC.1.1788GL003685* | BP:cell division;BP:cell cycle;CC:integral component of membrane;CC:cell outer membrane; | GO:0051301;GO:0007049;GO:0016021;GO:0009279 |  |
| *S.maltophilia.CGMCC.1.1788GL002724* | BP:nitrate assimilation;CC:integral component of membrane;MF:nitrate transmembrane transporter activity; | GO:0042128;GO:0016021;GO:0015112 |  |
| *S.maltophilia.CGMCC.1.1788GL003027* | CC:integral component of membrane; | GO:0016021 |  |
| *S.maltophilia.CGMCC.1.1788GL004324* | BP:transmembrane transport;CC:integral component of plasma membrane; | GO:0055085;GO:0005887 |  |
| *S.maltophilia.CGMCC.1.1788GL002798* | CC:integral component of membrane;MF:phosphorelay sensor kinase activity; | GO:0016021;GO:0000155 |  |
| *S.maltophilia.CGMCC.1.1788GL001200* | CC:integral component of membrane; | GO:0016021 |  |
| *S.maltophilia.CGMCC.1.1788GL000793* | CC:integral component of membrane;MF:transmembrane transporter activity; | GO:0016021;GO:0022857 |  |
| *S.maltophilia.CGMCC.1.1788GL004099* | CC:integral component of membrane;CC:plasma membrane;MF:ATP binding; | GO:0016021;GO:0005886;GO:0005524 |  |
| *S.maltophilia.CGMCC.1.1788GL001895* | BP:alanine biosynthetic process;BP:pantothenate biosynthetic process;CC:cytoplasm;MF:aspartate 1-decarboxylase activity; | GO:0006523;GO:0015940;GO:0005737;GO:0004068 |  |
| *S.maltophilia.CGMCC.1.1788GL002305* | BP:protein secretion;BP:bacterial-type flagellum assembly;CC:integral component of membrane;CC:plasma membrane; | GO:0009306;GO:0044780;GO:0016021;GO:0005886 |  |
| *S.maltophilia.CGMCC.1.1788GL002308* | BP:bacterial-type flagellum assembly;BP:protein targeting;CC:integral component of membrane;CC:plasma membrane;CC:bacterial-type flagellum basal body; | GO:0044780;GO:0006605;GO:0016021;GO:0005886;GO:0009425 |  |
| *S.maltophilia.CGMCC.1.1788GL001012* | BP:response to chemical;CC:integral component of plasma membrane;MF:transmembrane transporter activity; | GO:0042221;GO:0005887;GO:0022857 |  |
| *S.maltophilia.CGMCC.1.1788GL003641* | CC:integral component of membrane;CC:membrane; | GO:0016021;GO:0016020 |  |
| *S.maltophilia.CGMCC.1.1788GL002206* | BP:copper ion transport;CC:integral component of membrane;CC:plasma membrane;MF:cation-transporting ATPase activity;MF:ATPase activity;MF:copper ion binding;MF:ATP binding; | GO:0006825;GO:0016021;GO:0005886;GO:0019829;GO:0016887;GO:0005507;GO:0005524 |  |
| *S.maltophilia.CGMCC.1.1788GL004141* | CC:integral component of membrane;MF:transmembrane transporter activity; | GO:0016021;GO:0022857 |  |
| *S.maltophilia.CGMCC.1.1788GL003487* | CC:plasma membrane;MF:metal ion binding;MF:4 iron, 4 sulfur cluster binding;MF:electron carrier activity; | GO:0005886;GO:0046872;GO:0051539;GO:0009055 |  |
| *S.maltophilia.CGMCC.1.1788GL002598* | CC:membrane;CC:integral component of membrane;MF:transmembrane transporter activity; | GO:0016020;GO:0016021;GO:0022857 |  |
| *S.maltophilia.CGMCC.1.1788GL002046* | BP:queuosine biosynthetic process;CC:cytoplasm;MF:S-adenosylmethionin;MF:transferase activity;MF:isomerase activity; | GO:0008616;GO:0005737;GO:0051075;GO:0016740;GO:0016853 |  |
| *S.maltophilia.CGMCC.1.1788GL002198* | BP:histidine biosynthetic process;CC:cytoplasm;MF:imidazoleglycerol-phosphate synthase activity;MF:lyase activity; | GO:0000105;GO:0005737;GO:0000107;GO:0016829 |  |
| *S.maltophilia.CGMCC.1.1788GL002196* | BP:glutamine metabolic process;BP:histidine biosynthetic process;CC:cytoplasm;MF:hydrolase activity;MF:glutaminase activity;MF:lyase activity;MF:imidazoleglycerol-phosphate synthase activity; | GO:0006541;GO:0000105;GO:0005737;GO:0016787;GO:0004359;GO:0016829;GO:0000107 |  |
| *S.maltophilia.CGMCC.1.1788GL002197* | BP:histidine biosynthetic process;CC:cytoplasm;MF:1; | GO:0000105;GO:0005737;GO:0003949 |  |
| *S.maltophilia.CGMCC.1.1788GL002195* | BP:histidine biosynthetic process;CC:cytoplasm;MF:metal ion binding;MF:imidazoleglycerol-phosphate dehydratase activity;MF:histidinol-phosphatase activity; | GO:0000105;GO:0005737;GO:0046872;GO:0004424;GO:0004401 |  |
| *S.maltophilia.CGMCC.1.1788GL004246* | CC:integral component of membrane;MF:phosphatidate cytidylyltransferase activity;MF:nucleotidyltransferase activity;MF:transferase activity, transferring acyl groups; | GO:0016021;GO:0004605;GO:0016779;GO:0016746 |  |
| *S.maltophilia.CGMCC.1.1788GL000592* | CC:integral component of membrane;CC:plasma membrane; | GO:0016021;GO:0005886 |  |
| *S.maltophilia.CGMCC.1.1788GL003876* | BP:protein transport;CC:integral component of membrane;CC:plasma membrane;MF:transmembrane transporter activity; | GO:0015031;GO:0016021;GO:0005886;GO:0022857 |  |
| *S.maltophilia.CGMCC.1.1788GL003875* | BP:protein transport;CC:integral component of membrane;CC:plasma membrane;MF:transmembrane transporter activity; | GO:0015031;GO:0016021;GO:0005886;GO:0022857 |  |
| *S.maltophilia.CGMCC.1.1788GL003121* | CC:outer membrane; | GO:0019867 |  |
| *S.maltophilia.CGMCC.1.1788GL003122* | CC:integral component of membrane; | GO:0016021 |  |
| *S.maltophilia.CGMCC.1.1788GL004526* | CC:integral component of membrane; | GO:0016021 |  |
| *novel0307* | MULTISPECIES: peptidoglycan-associated lipoprotein Pal [Stenotrophomonas] | GO:0051301;GO:0007049;GO:0016021;GO:0009279 |  |
| *S.maltophilia.CGMCC.1.1788GL002185* | BP:phosphorylation;BP:threonine biosynthetic process;CC:cytoplasm;MF:homoserine kinase activity;MF:ATP binding; | GO:0016310;GO:0009088;GO:0005737;GO:0004413;GO:0005524 |  |
| *S.maltophilia.CGMCC.1.1788GL000746* | CC:integral component of membrane;CC:plasma membrane; | GO:0016021;GO:0005886 |  |
| *S.maltophilia.CGMCC.1.1788GL000362* | CC:integral component of plasma membrane;MF:potassium ion binding;MF:potassium-transporting ATPase activity; | GO:0005887;GO:0030955;GO:0008556 |  |
| *S.maltophilia.CGMCC.1.1788GL003865* | CC:integral component of membrane; | GO:0016021 |  |
| *S.maltophilia.CGMCC.1.1788GL004249* | BP:protein dephosphorylation;CC:integral component of membrane;MF:protein tyrosine/serine/threonine phosphatase activity; | GO:0006470;GO:0016021;GO:0008138 |  |
| *S.maltophilia.CGMCC.1.1788GL003590* | BP:tricarboxylic acid cycle;CC:cytoplasm;MF:transferase activity, transferring acyl groups, acyl groups converted into alkyl on transfer;MF:citrate synthase activity; | GO:0006099;GO:0005737;GO:0046912;GO:0036440 |  |
| cellular_component | protein-containing complex | GO:0032991 |  |
| *S.maltophilia.CGMCC.1.1788GL002082* | BP:translation;CC:ribonucleoprotein complex;CC:ribosome;MF:structural constituent of ribosome;MF:RNA binding; | GO:0006412;GO:1990904;GO:0005840;GO:0003735;GO:0003723 |  |
| *S.maltophilia.CGMCC.1.1788GL002729* | BP:nitrate metabolic process;CC:plasma membrane;CC:nitrate reductase complex;MF:nitrate reductase activity;MF:molybdopterin cofactor binding;MF:4 iron, 4 sulfur cluster binding;MF:metal ion binding; | GO:0042126;GO:0005886;GO:0009325;GO:0008940;GO:0043546;GO:0051539;GO:0046872 |  |
| *S.maltophilia.CGMCC.1.1788GL002728* | BP:nitrate metabolic process;CC:nitrate reductase complex;MF:nitrate reductase activity; | GO:0042126;GO:0009325;GO:0008940 |  |
| *S.maltophilia.CGMCC.1.1788GL002726* | BP:nitrate assimilation;CC:nitrate reductase complex;CC:integral component of membrane;CC:plasma membrane;MF:nitrate reductase activity;MF:metal ion binding; | GO:0042128;GO:0009325;GO:0016021;GO:0005886;GO:0008940;GO:0046872 |  |
| *S.maltophilia.CGMCC.1.1788GL000534* | BP:ion transport;CC:cell outer membrane;CC:pore complex;MF:porin activity;MF:cobalamin-transporting ATPase activity; | GO:0006811;GO:0009279;GO:0046930;GO:0015288;GO:0015420 |  |
| *S.maltophilia.CGMCC.1.1788GL000846* | BP:transcription, DNA-templated;CC:DNA-directed RNA polymerase complex;MF:magnesium ion binding;MF:zinc ion binding;MF:DNA binding;MF:DNA-directed RNA polymerase activity; | GO:0006351;GO:0000428;GO:0000287;GO:0008270;GO:0003677;GO:0003899 |  |
| *S.maltophilia.CGMCC.1.1788GL000843* | BP:translation;CC:ribonucleoprotein complex;CC:ribosome;MF:structural constituent of ribosome; | GO:0006412;GO:1990904;GO:0005840;GO:0003735 |  |
| *S.maltophilia.CGMCC.1.1788GL001719* | BP:protein secretion by the type II secretion system;CC:integral component of membrane;CC:type II protein secretion system complex;CC:plasma membrane; | GO:0015628;GO:0016021;GO:0015627;GO:0005886 |  |
| *S.maltophilia.CGMCC.1.1788GL000876* | BP:translation;CC:small ribosomal subunit;MF:structural constituent of ribosome;MF:rRNA binding; | GO:0006412;GO:0015935;GO:0003735;GO:0019843 |  |
| *S.maltophilia.CGMCC.1.1788GL000852* | BP:translation;CC:ribonucleoprotein complex;CC:ribosome;MF:structural constituent of ribosome;MF:tRNA binding; | GO:0006412;GO:1990904;GO:0005840;GO:0003735;GO:0000049 |  |
| *S.maltophilia.CGMCC.1.1788GL000877* | BP:transcription, DNA-templated;CC:cytoplasm;CC:DNA-directed RNA polymerase complex;MF:DNA binding;MF:DNA-directed RNA polymerase activity;MF:protein dimerization activity; | GO:0006351;GO:0005737;GO:0000428;GO:0003677;GO:0003899;GO:0046983 |  |
| *S.maltophilia.CGMCC.1.1788GL004070* | BP:protein unfolding;BP:proteolysis;CC:HslUV protease complex;MF:proteasome-activating ATPase activity;MF:ATPase activity;MF:peptidase activity;MF:ATP binding; | GO:0043335;GO:0006508;GO:0009376;GO:0036402;GO:0016887;GO:0008233;GO:0005524 |  |
| *S.maltophilia.CGMCC.1.1788GL001652* | BP:phosphate ion transmembrane transport;CC:ATP-binding cassett;MF:phosphate ion binding; | GO:0035435;GO:0043190;GO:0042301 |  |
| *novel0106* | hypothetical protein, partial [Corallococcus sp. AB049A] | GO:0006351;GO:0000428;GO:0000287;GO:0008270;GO:0003677;GO:0003899 |  |
| *novel0104* | DNA-directed RNA polymerase subunit beta [Stenotrophomonas maltophilia] | GO:0006351;GO:0000428;GO:0003899;GO:0032549;GO:0003677 |  |
| *novel0109* | hypothetical protein, partial [Enterobacter hormaechei] | GO:0006351;GO:0000428;GO:0000287;GO:0008270;GO:0003677;GO:0003899 |  |
| molecular_function | ATP-dependent activity | GO:0140657 |  |
| *S.maltophilia.CGMCC.1.1788GL000534* | BP:ion transport;CC:cell outer membrane;CC:pore complex;MF:porin activity;MF:cobalamin-transporting ATPase activity; | GO:0006811;GO:0009279;GO:0046930;GO:0015288;GO:0015420 |  |
| *S.maltophilia.CGMCC.1.1788GL004070* | BP:protein unfolding;BP:proteolysis;CC:HslUV protease complex;MF:proteasome-activating ATPase activity;MF:ATPase activity;MF:peptidase activity;MF:ATP binding; | GO:0043335;GO:0006508;GO:0009376;GO:0036402;GO:0016887;GO:0008233;GO:0005524 |  |
| *S.maltophilia.CGMCC.1.1788GL002037* | CC:integral component of membrane;MF:ABC-type transmembrane transporter activity;MF:ATP binding; | GO:0016021;GO:0140359;GO:0005524 |  |
| *S.maltophilia.CGMCC.1.1788GL002206* | BP:copper ion transport;CC:integral component of membrane;CC:plasma membrane;MF:cation-transporting ATPase activity;MF:ATPase activity;MF:copper ion binding;MF:ATP binding; | GO:0006825;GO:0016021;GO:0005886;GO:0019829;GO:0016887;GO:0005507;GO:0005524 |  |
| *S.maltophilia.CGMCC.1.1788GL000362* | CC:integral component of plasma membrane;MF:potassium ion binding;MF:potassium-transporting ATPase activity; | GO:0005887;GO:0030955;GO:0008556 |  |
| molecular_function | antioxidant activity | GO:0016209 |  |
| *S.maltophilia.CGMCC.1.1788GL002776* | MF:superoxide dismutase activity;MF:metal ion binding; | GO:0004784;GO:0046872 |  |
| *S.maltophilia.CGMCC.1.1788GL004698* | BP:response to oxidative stress;MF:glutathione peroxidase activity; | GO:0006979;GO:0004602 |  |
| *novel0014* | Dyp-type peroxidase [Stenotrophomonas sp. KAs 5-3] | GO:0004601;GO:0020037 |  |
| *novel0012* | Superoxide dismutase-like protein YojM [Stenotrophomonas maltophilia] | GO:0004784;GO:0046872 |  |
| molecular_function | binding | GO:0005488 |  |
| *S.maltophilia.CGMCC.1.1788GL003134* | BP:generation of precursor metabolites and energy;MF:metal ion binding;MF:4 iron, 4 sulfur cluster binding;MF:fumarate hydratase activity; | GO:0006091;GO:0046872;GO:0051539;GO:0004333 |  |
| *S.maltophilia.CGMCC.1.1788GL000828* | MF:ribosomal large subunit binding;MF:ribosome binding;MF:ATP binding;MF:ATPase activity;MF:GTP binding; | GO:0043023;GO:0043022;GO:0005524;GO:0016887;GO:0005525 |  |
| *S.maltophilia.CGMCC.1.1788GL002082* | BP:translation;CC:ribonucleoprotein complex;CC:ribosome;MF:structural constituent of ribosome;MF:RNA binding; | GO:0006412;GO:1990904;GO:0005840;GO:0003735;GO:0003723 |  |
| *S.maltophilia.CGMCC.1.1788GL002732* | BP:Mo-molybdopterin cofactor biosynthetic process;MF:nucleotidyltransferase activity;MF:catalytic activity;MF:GTP binding; | GO:0006777;GO:0016779;GO:0003824;GO:0005525 |  |
| *S.maltophilia.CGMCC.1.1788GL001601* | BP:phosphorylation;BP:'de novo' CTP biosynthetic process;CC:cytoplasm;MF:UMP kinase activity;MF:ATP binding; | GO:0016310;GO:0044210;GO:0005737;GO:0033862;GO:0005524 |  |
| *S.maltophilia.CGMCC.1.1788GL002729* | BP:nitrate metabolic process;CC:plasma membrane;CC:nitrate reductase complex;MF:nitrate reductase activity;MF:molybdopterin cofactor binding;MF:4 iron, 4 sulfur cluster binding;MF:metal ion binding; | GO:0042126;GO:0005886;GO:0009325;GO:0008940;GO:0043546;GO:0051539;GO:0046872 |  |
| *S.maltophilia.CGMCC.1.1788GL002098* | BP:ribosome biogenesis;MF:GTP binding; | GO:0042254;GO:0005525 |  |
| *S.maltophilia.CGMCC.1.1788GL002726* | BP:nitrate assimilation;CC:nitrate reductase complex;CC:integral component of membrane;CC:plasma membrane;MF:nitrate reductase activity;MF:metal ion binding; | GO:0042128;GO:0009325;GO:0016021;GO:0005886;GO:0008940;GO:0046872 |  |
| *S.maltophilia.CGMCC.1.1788GL002940* | MF:oxidoreductase activity;MF:FMN binding; | GO:0016491;GO:0010181 |  |
| *S.maltophilia.CGMCC.1.1788GL002632* | MF:transcription factor activity, sequence-specific DNA binding;MF:sequence-specific DNA binding; | GO:0003700;GO:0043565 |  |
| *S.maltophilia.CGMCC.1.1788GL001455* | MF:DNA binding; | GO:0003677 |  |
| *S.maltophilia.CGMCC.1.1788GL003625* | BP:protein folding;CC:cytoplasm;MF:unfolded protein binding; | GO:0006457;GO:0005737;GO:0051082 |  |
| *S.maltophilia.CGMCC.1.1788GL003158* | BP:enzyme-directed rRNA pseudouridine synthesis;MF:rRNA pseudouridine synthase activity;MF:lyase activity;MF:RNA binding;MF:hydrolase activity, acting on glycosyl bonds; | GO:0000455;GO:0120159;GO:0016829;GO:0003723;GO:0016798 |  |
| *S.maltophilia.CGMCC.1.1788GL001955* | ------ | GO:0006355;GO:0003677 |  |
| *S.maltophilia.CGMCC.1.1788GL002153* | MF:DNA binding; | GO:0003677 |  |
| *S.maltophilia.CGMCC.1.1788GL000846* | BP:transcription, DNA-templated;CC:DNA-directed RNA polymerase complex;MF:magnesium ion binding;MF:zinc ion binding;MF:DNA binding;MF:DNA-directed RNA polymerase activity; | GO:0006351;GO:0000428;GO:0000287;GO:0008270;GO:0003677;GO:0003899 |  |
| *S.maltophilia.CGMCC.1.1788GL002718* | BP:cysteine biosynthetic process;MF:oxidoreductase activity;MF:FMN binding; | GO:0019344;GO:0016491;GO:0010181 |  |
| *S.maltophilia.CGMCC.1.1788GL001603* | CC:cytoplasm;MF:translation elongation factor activity; | GO:0005737;GO:0003746 |  |
| *S.maltophilia.CGMCC.1.1788GL002572* | BP:regulation of transcription, DNA-templated;MF:sequence-specific DNA binding;MF:ATP binding; | GO:0006355;GO:0043565;GO:0005524 |  |
| *S.maltophilia.CGMCC.1.1788GL003898* | BP:valine biosynthetic process;BP:isoleucine biosynthetic process;MF:NADP binding;MF:isomerase activity;MF:magnesium ion binding;MF:ketol-acid reductoisomerase activity; | GO:0009099;GO:0009097;GO:0050661;GO:0016853;GO:0000287;GO:0004455 |  |
| *S.maltophilia.CGMCC.1.1788GL000876* | BP:translation;CC:small ribosomal subunit;MF:structural constituent of ribosome;MF:rRNA binding; | GO:0006412;GO:0015935;GO:0003735;GO:0019843 |  |
| *S.maltophilia.CGMCC.1.1788GL001927* | MF:16S rRN;MF:nucleic acid binding; | GO:0052913;GO:0003676 |  |
| *novel0277* | S1 RNA-binding domain-containing protein, partial [Stenotrophomonas maltophilia] | GO:0006402;GO:0006396;GO:0005737;GO:0000287;GO:0004654;GO:0003723 |  |
| *S.maltophilia.CGMCC.1.1788GL000852* | BP:translation;CC:ribonucleoprotein complex;CC:ribosome;MF:structural constituent of ribosome;MF:tRNA binding; | GO:0006412;GO:1990904;GO:0005840;GO:0003735;GO:0000049 |  |
| *S.maltophilia.CGMCC.1.1788GL000558* | MF:catalytic activity;MF:iron-sulfur cluster binding;MF:metal ion binding; | GO:0003824;GO:0051536;GO:0046872 |  |
| *S.maltophilia.CGMCC.1.1788GL002776* | MF:superoxide dismutase activity;MF:metal ion binding; | GO:0004784;GO:0046872 |  |
| *S.maltophilia.CGMCC.1.1788GL002777* | MF:oxidoreductase activity, acting on the CH-NH group of donors, NAD or NADP as acceptor;MF:FMN binding; | GO:0016646;GO:0010181 |  |
| *S.maltophilia.CGMCC.1.1788GL001733* | BP:phosphorelay signal transduction system;BP:regulation of transcription, DNA-templated;MF:DNA binding; | GO:0000160;GO:0006355;GO:0003677 |  |
| *S.maltophilia.CGMCC.1.1788GL000280* | BP:viral capsid assembly;MF:ATP binding; | GO:0019069;GO:0005524 |  |
| *S.maltophilia.CGMCC.1.1788GL003981* | MF:alcohol dehydrogenas;MF:alcohol dehydrogenas;MF:zinc ion binding;MF:oxidoreductase activity, acting on the CH-OH group of donors, NAD or NADP as acceptor; | GO:0008106;GO:0004022;GO:0008270;GO:0016616 |  |
| *S.maltophilia.CGMCC.1.1788GL000877* | BP:transcription, DNA-templated;CC:cytoplasm;CC:DNA-directed RNA polymerase complex;MF:DNA binding;MF:DNA-directed RNA polymerase activity;MF:protein dimerization activity; | GO:0006351;GO:0005737;GO:0000428;GO:0003677;GO:0003899;GO:0046983 |  |
| *S.maltophilia.CGMCC.1.1788GL000500* | BP:proteolysis;MF:metal ion binding;MF:metalloendopeptidase activity; | GO:0006508;GO:0046872;GO:0004222 |  |
| *S.maltophilia.CGMCC.1.1788GL004070* | BP:protein unfolding;BP:proteolysis;CC:HslUV protease complex;MF:proteasome-activating ATPase activity;MF:ATPase activity;MF:peptidase activity;MF:ATP binding; | GO:0043335;GO:0006508;GO:0009376;GO:0036402;GO:0016887;GO:0008233;GO:0005524 |  |
| *S.maltophilia.CGMCC.1.1788GL001822* | CC:cytoplasm;MF:metal ion binding;MF:GTPase activity;MF:GTP binding; | GO:0005737;GO:0046872;GO:0003924;GO:0005525 |  |
| *novel0156* | MULTISPECIES: bacterioferritin [Stenotrophomonas] | GO:0006879;GO:0006826;GO:0008199;GO:0004322 |  |
| *S.maltophilia.CGMCC.1.1788GL004052* | BP:regulation of cell shape;BP:cell morphogenesis;CC:cytoplasm;MF:ATP binding; | GO:0008360;GO:0000902;GO:0005737;GO:0005524 |  |
| *S.maltophilia.CGMCC.1.1788GL002026* | BP:negative regulation of transcription, DNA-templated;MF:DNA binding; | GO:0045892;GO:0003677 |  |
| *novel0014* | Dyp-type peroxidase [Stenotrophomonas sp. KAs 5-3] | GO:0004601;GO:0020037 |  |
| *S.maltophilia.CGMCC.1.1788GL000084* | MF:exonuclease activity;MF:nucleic acid binding; | GO:0004527;GO:0003676 |  |
| *S.maltophilia.CGMCC.1.1788GL000319* | MF:hydrolase activity;MF:ATP binding; | GO:0016787;GO:0005524 |  |
| *S.maltophilia.CGMCC.1.1788GL002037* | CC:integral component of membrane;MF:ABC-type transmembrane transporter activity;MF:ATP binding; | GO:0016021;GO:0140359;GO:0005524 |  |
| *S.maltophilia.CGMCC.1.1788GL002324* | BP:phosphorelay signal transduction system;MF:DNA binding; | GO:0000160;GO:0003677 |  |
| *S.maltophilia.CGMCC.1.1788GL002811* | BP:regulation of transcription, DNA-templated;MF:DNA binding; | GO:0006355;GO:0003677 |  |
| *S.maltophilia.CGMCC.1.1788GL000416* | CC:cell outer membrane;MF:carbohydrate binding; | GO:0009279;GO:0030246 |  |
| *S.maltophilia.CGMCC.1.1788GL001446* | BP:transposition, DNA-mediated;MF:transposase activity;MF:DNA binding; | GO:0006313;GO:0004803;GO:0003677 |  |
| *S.maltophilia.CGMCC.1.1788GL003683* | BP:queuosine biosynthetic process;MF:magnesium ion binding;MF:S-adenosyl-L-methionine binding;MF:4 iron, 4 sulfur cluster binding;MF:carbon-nitrogen lyase activity; | GO:0008616;GO:0000287;GO:1904047;GO:0051539;GO:0016840 |  |
| *novel0012* | Superoxide dismutase-like protein YojM [Stenotrophomonas maltophilia] | GO:0004784;GO:0046872 |  |
| *S.maltophilia.CGMCC.1.1788GL004099* | CC:integral component of membrane;CC:plasma membrane;MF:ATP binding; | GO:0016021;GO:0005886;GO:0005524 |  |
| *S.maltophilia.CGMCC.1.1788GL004098* | MF:ATP binding; | GO:0005524 |  |
| *S.maltophilia.CGMCC.1.1788GL004090* | BP:proteolysis;MF:metal ion binding;MF:metalloendopeptidase activity; | GO:0006508;GO:0046872;GO:0004222 |  |
| *S.maltophilia.CGMCC.1.1788GL004601* | BP:response to stimulus;BP:DNA-templated transcription, initiation;MF:sigma factor activity;MF:DNA binding; | GO:0050896;GO:0006352;GO:0016987;GO:0003677 |  |
| *S.maltophilia.CGMCC.1.1788GL003780* | MF:potassium ion binding;MF:magnesium ion binding;MF:pyruvate kinase activity;MF:kinase activity;MF:ATP binding; | GO:0030955;GO:0000287;GO:0004743;GO:0016301;GO:0005524 |  |
| *S.maltophilia.CGMCC.1.1788GL001092* | MF:ATP binding; | GO:0005524 |  |
| *S.maltophilia.CGMCC.1.1788GL001652* | BP:phosphate ion transmembrane transport;CC:ATP-binding cassett;MF:phosphate ion binding; | GO:0035435;GO:0043190;GO:0042301 |  |
| *novel0021* | acetyl-CoA carboxylase biotin carboxylase subunit, partial [Stenotrophomonas sp. KAs 5-3] | GO:0046872;GO:0016874;GO:0005524 |  |
| *novel0020* | 3-methylcrotonyl-CoA carboxylase, partial [Bacillus sp. Nf3] | GO:0046872;GO:0016874;GO:0005524 |  |
| *novel0025* | MULTISPECIES: isovaleryl-CoA dehydrogenase [Stenotrophomonas] | GO:1901565;GO:0003995;GO:0050660 |  |
| *S.maltophilia.CGMCC.1.1788GL002206* | BP:copper ion transport;CC:integral component of membrane;CC:plasma membrane;MF:cation-transporting ATPase activity;MF:ATPase activity;MF:copper ion binding;MF:ATP binding; | GO:0006825;GO:0016021;GO:0005886;GO:0019829;GO:0016887;GO:0005507;GO:0005524 |  |
| *S.maltophilia.CGMCC.1.1788GL002202* | BP:glycolytic process;MF:glucokinase activity;MF:ATP binding;MF:glucose binding; | GO:0006096;GO:0004340;GO:0005524;GO:0005536 |  |
| *novel0106* | hypothetical protein, partial [Corallococcus sp. AB049A] | GO:0006351;GO:0000428;GO:0000287;GO:0008270;GO:0003677;GO:0003899 |  |
| *novel0104* | DNA-directed RNA polymerase subunit beta [Stenotrophomonas maltophilia] | GO:0006351;GO:0000428;GO:0003899;GO:0032549;GO:0003677 |  |
| *novel0109* | hypothetical protein, partial [Enterobacter hormaechei] | GO:0006351;GO:0000428;GO:0000287;GO:0008270;GO:0003677;GO:0003899 |  |
| *S.maltophilia.CGMCC.1.1788GL002671* | BP:DNA-templated transcription, initiation;MF:sigma factor activity;MF:DNA binding; | GO:0006352;GO:0016987;GO:0003677 |  |
| *S.maltophilia.CGMCC.1.1788GL003487* | CC:plasma membrane;MF:metal ion binding;MF:4 iron, 4 sulfur cluster binding;MF:electron carrier activity; | GO:0005886;GO:0046872;GO:0051539;GO:0009055 |  |
| *S.maltophilia.CGMCC.1.1788GL002194* | BP:histidine biosynthetic process;MF:pyridoxal phosphate binding;MF:histidinol-phosphate transaminase activity; | GO:0000105;GO:0030170;GO:0004400 |  |
| *S.maltophilia.CGMCC.1.1788GL002195* | BP:histidine biosynthetic process;CC:cytoplasm;MF:metal ion binding;MF:imidazoleglycerol-phosphate dehydratase activity;MF:histidinol-phosphatase activity; | GO:0000105;GO:0005737;GO:0046872;GO:0004424;GO:0004401 |  |
| *S.maltophilia.CGMCC.1.1788GL003589* | BP:propionate metabolic process, methylcitrate cycle;MF:citrate dehydratase activity;MF:aconitate hydratase activity;MF:iron-sulfur cluster binding;MF:metal ion binding; | GO:0019679;GO:0047780;GO:0003994;GO:0051536;GO:0046872 |  |
| *S.maltophilia.CGMCC.1.1788GL002185* | BP:phosphorylation;BP:threonine biosynthetic process;CC:cytoplasm;MF:homoserine kinase activity;MF:ATP binding; | GO:0016310;GO:0009088;GO:0005737;GO:0004413;GO:0005524 |  |
| *S.maltophilia.CGMCC.1.1788GL002180* | MF:transcription factor activity, sequence-specific DNA binding;MF:sequence-specific DNA binding; | GO:0003700;GO:0043565 |  |
| *S.maltophilia.CGMCC.1.1788GL000362* | CC:integral component of plasma membrane;MF:potassium ion binding;MF:potassium-transporting ATPase activity; | GO:0005887;GO:0030955;GO:0008556 |  |
| *S.maltophilia.CGMCC.1.1788GL002980* | MF:FAD binding; | GO:0071949 |  |
| molecular_function | catalytic activity | GO:0003824 |  |
| *S.maltophilia.CGMCC.1.1788GL003134* | BP:generation of precursor metabolites and energy;MF:metal ion binding;MF:4 iron, 4 sulfur cluster binding;MF:fumarate hydratase activity; | GO:0006091;GO:0046872;GO:0051539;GO:0004333 |  |
| *S.maltophilia.CGMCC.1.1788GL000828* | MF:ribosomal large subunit binding;MF:ribosome binding;MF:ATP binding;MF:ATPase activity;MF:GTP binding; | GO:0043023;GO:0043022;GO:0005524;GO:0016887;GO:0005525 |  |
| *S.maltophilia.CGMCC.1.1788GL002732* | BP:Mo-molybdopterin cofactor biosynthetic process;MF:nucleotidyltransferase activity;MF:catalytic activity;MF:GTP binding; | GO:0006777;GO:0016779;GO:0003824;GO:0005525 |  |
| *S.maltophilia.CGMCC.1.1788GL001601* | BP:phosphorylation;BP:'de novo' CTP biosynthetic process;CC:cytoplasm;MF:UMP kinase activity;MF:ATP binding; | GO:0016310;GO:0044210;GO:0005737;GO:0033862;GO:0005524 |  |
| *S.maltophilia.CGMCC.1.1788GL000393* | BP:heme O biosynthetic process;CC:integral component of membrane;CC:plasma membrane;MF:protoheme IX farnesyltransferase activity; | GO:0048034;GO:0016021;GO:0005886;GO:0008495 |  |
| *S.maltophilia.CGMCC.1.1788GL003149* | MF:catalytic activity; | GO:0003824 |  |
| *S.maltophilia.CGMCC.1.1788GL000831* | MF:phosphorelay sensor kinase activity; | GO:0000155 |  |
| *S.maltophilia.CGMCC.1.1788GL002729* | BP:nitrate metabolic process;CC:plasma membrane;CC:nitrate reductase complex;MF:nitrate reductase activity;MF:molybdopterin cofactor binding;MF:4 iron, 4 sulfur cluster binding;MF:metal ion binding; | GO:0042126;GO:0005886;GO:0009325;GO:0008940;GO:0043546;GO:0051539;GO:0046872 |  |
| *S.maltophilia.CGMCC.1.1788GL002728* | BP:nitrate metabolic process;CC:nitrate reductase complex;MF:nitrate reductase activity; | GO:0042126;GO:0009325;GO:0008940 |  |
| *S.maltophilia.CGMCC.1.1788GL002726* | BP:nitrate assimilation;CC:nitrate reductase complex;CC:integral component of membrane;CC:plasma membrane;MF:nitrate reductase activity;MF:metal ion binding; | GO:0042128;GO:0009325;GO:0016021;GO:0005886;GO:0008940;GO:0046872 |  |
| *S.maltophilia.CGMCC.1.1788GL002940* | MF:oxidoreductase activity;MF:FMN binding; | GO:0016491;GO:0010181 |  |
| *S.maltophilia.CGMCC.1.1788GL001701* | BP:peptidoglycan metabolic process;CC:membrane;MF:lytic transglycosylase activity; | GO:0000270;GO:0016020;GO:0008933 |  |
| *S.maltophilia.CGMCC.1.1788GL003158* | BP:enzyme-directed rRNA pseudouridine synthesis;MF:rRNA pseudouridine synthase activity;MF:lyase activity;MF:RNA binding;MF:hydrolase activity, acting on glycosyl bonds; | GO:0000455;GO:0120159;GO:0016829;GO:0003723;GO:0016798 |  |
| *S.maltophilia.CGMCC.1.1788GL001953* | MF:catalytic activity;MF:exonuclease activity;MF:endonuclease activity; | GO:0003824;GO:0004527;GO:0004519 |  |
| *S.maltophilia.CGMCC.1.1788GL001956* | MF:protein deglycase activity; | GO:0036524 |  |
| *S.maltophilia.CGMCC.1.1788GL000846* | BP:transcription, DNA-templated;CC:DNA-directed RNA polymerase complex;MF:magnesium ion binding;MF:zinc ion binding;MF:DNA binding;MF:DNA-directed RNA polymerase activity; | GO:0006351;GO:0000428;GO:0000287;GO:0008270;GO:0003677;GO:0003899 |  |
| *S.maltophilia.CGMCC.1.1788GL002718* | BP:cysteine biosynthetic process;MF:oxidoreductase activity;MF:FMN binding; | GO:0019344;GO:0016491;GO:0010181 |  |
| *S.maltophilia.CGMCC.1.1788GL000711* | CC:integral component of membrane;MF:hydrolase activity; | GO:0016021;GO:0016787 |  |
| *S.maltophilia.CGMCC.1.1788GL003898* | BP:valine biosynthetic process;BP:isoleucine biosynthetic process;MF:NADP binding;MF:isomerase activity;MF:magnesium ion binding;MF:ketol-acid reductoisomerase activity; | GO:0009099;GO:0009097;GO:0050661;GO:0016853;GO:0000287;GO:0004455 |  |
| *S.maltophilia.CGMCC.1.1788GL001927* | MF:16S rRN;MF:nucleic acid binding; | GO:0052913;GO:0003676 |  |
| *novel0277* | S1 RNA-binding domain-containing protein, partial [Stenotrophomonas maltophilia] | GO:0006402;GO:0006396;GO:0005737;GO:0000287;GO:0004654;GO:0003723 |  |
| *S.maltophilia.CGMCC.1.1788GL001495* | BP:nitrogen compound metabolic process;BP:primary metabolic process;BP:macromolecule metabolic process;MF:catalytic activity; | GO:0006807;GO:0044238;GO:0043170;GO:0003824 |  |
| *S.maltophilia.CGMCC.1.1788GL003953* | BP:carbohydrate metabolic process;MF:hydrolase activity, acting on carbon-nitroge; | GO:0005975;GO:0016810 |  |
| *S.maltophilia.CGMCC.1.1788GL003554* | CC:integral component of membrane;CC:plasma membrane;MF:transferase activity, transferring phosphorus-containing groups;MF:sulfuric ester hydrolase activity; | GO:0016021;GO:0005886;GO:0016772;GO:0008484 |  |
| *S.maltophilia.CGMCC.1.1788GL000558* | MF:catalytic activity;MF:iron-sulfur cluster binding;MF:metal ion binding; | GO:0003824;GO:0051536;GO:0046872 |  |
| *S.maltophilia.CGMCC.1.1788GL002178* | MF:hydrolase activity;MF:choloylglycine hydrolase activity; | GO:0016787;GO:0045302 |  |
| *S.maltophilia.CGMCC.1.1788GL002776* | MF:superoxide dismutase activity;MF:metal ion binding; | GO:0004784;GO:0046872 |  |
| *S.maltophilia.CGMCC.1.1788GL002777* | MF:oxidoreductase activity, acting on the CH-NH group of donors, NAD or NADP as acceptor;MF:FMN binding; | GO:0016646;GO:0010181 |  |
| *S.maltophilia.CGMCC.1.1788GL000199* | BP:intracellular signal transduction;BP:cyclic nucleotide biosynthetic process;MF:adenylate cyclase activity; | GO:0035556;GO:0009190;GO:0004016 |  |
| *S.maltophilia.CGMCC.1.1788GL003981* | MF:alcohol dehydrogenas;MF:alcohol dehydrogenas;MF:zinc ion binding;MF:oxidoreductase activity, acting on the CH-OH group of donors, NAD or NADP as acceptor; | GO:0008106;GO:0004022;GO:0008270;GO:0016616 |  |
| *S.maltophilia.CGMCC.1.1788GL000877* | BP:transcription, DNA-templated;CC:cytoplasm;CC:DNA-directed RNA polymerase complex;MF:DNA binding;MF:DNA-directed RNA polymerase activity;MF:protein dimerization activity; | GO:0006351;GO:0005737;GO:0000428;GO:0003677;GO:0003899;GO:0046983 |  |
| *S.maltophilia.CGMCC.1.1788GL000724* | BP:proteolysis;MF:serine-type peptidase activity; | GO:0006508;GO:0008236 |  |
| *S.maltophilia.CGMCC.1.1788GL001233* | BP:fatty acid biosynthetic process;BP:cellular carbohydrate metabolic process;MF:carboxy-lyase activity; | GO:0006633;GO:0044262;GO:0016831 |  |
| *S.maltophilia.CGMCC.1.1788GL000500* | BP:proteolysis;MF:metal ion binding;MF:metalloendopeptidase activity; | GO:0006508;GO:0046872;GO:0004222 |  |
| *S.maltophilia.CGMCC.1.1788GL004070* | BP:protein unfolding;BP:proteolysis;CC:HslUV protease complex;MF:proteasome-activating ATPase activity;MF:ATPase activity;MF:peptidase activity;MF:ATP binding; | GO:0043335;GO:0006508;GO:0009376;GO:0036402;GO:0016887;GO:0008233;GO:0005524 |  |
| *S.maltophilia.CGMCC.1.1788GL004698* | BP:response to oxidative stress;MF:glutathione peroxidase activity; | GO:0006979;GO:0004602 |  |
| *S.maltophilia.CGMCC.1.1788GL001822* | CC:cytoplasm;MF:metal ion binding;MF:GTPase activity;MF:GTP binding; | GO:0005737;GO:0046872;GO:0003924;GO:0005525 |  |
| *novel0156* | MULTISPECIES: bacterioferritin [Stenotrophomonas] | GO:0006879;GO:0006826;GO:0008199;GO:0004322 |  |
| *novel0083* | hypothetical protein, partial [Mesorhizobium sp. M8A.F.Ca.ET.161.01.1.1] | GO:0016021;GO:0016787 |  |
| *S.maltophilia.CGMCC.1.1788GL002113* | BP:glutamine metabolic process;MF:transferase activity; | GO:0006541;GO:0016740 |  |
| *S.maltophilia.CGMCC.1.1788GL002110* | BP:nucleoside metabolic process;MF:catalytic activity; | GO:0009116;GO:0003824 |  |
| *novel0014* | Dyp-type peroxidase [Stenotrophomonas sp. KAs 5-3] | GO:0004601;GO:0020037 |  |
| *S.maltophilia.CGMCC.1.1788GL000084* | MF:exonuclease activity;MF:nucleic acid binding; | GO:0004527;GO:0003676 |  |
| *S.maltophilia.CGMCC.1.1788GL000319* | MF:hydrolase activity;MF:ATP binding; | GO:0016787;GO:0005524 |  |
| *S.maltophilia.CGMCC.1.1788GL003354* | BP:nitrogen compound metabolic process;MF:amino-acid racemase activity; | GO:0006807;GO:0047661 |  |
| *S.maltophilia.CGMCC.1.1788GL002643* | MF:oxidoreductase activity; | GO:0016491 |  |
| *S.maltophilia.CGMCC.1.1788GL001446* | BP:transposition, DNA-mediated;MF:transposase activity;MF:DNA binding; | GO:0006313;GO:0004803;GO:0003677 |  |
| *S.maltophilia.CGMCC.1.1788GL003683* | BP:queuosine biosynthetic process;MF:magnesium ion binding;MF:S-adenosyl-L-methionine binding;MF:4 iron, 4 sulfur cluster binding;MF:carbon-nitrogen lyase activity; | GO:0008616;GO:0000287;GO:1904047;GO:0051539;GO:0016840 |  |
| *S.maltophilia.CGMCC.1.1788GL002725* | MF:peptidyl-prolyl cis-trans isomerase activity; | GO:0003755 |  |
| *novel0012* | Superoxide dismutase-like protein YojM [Stenotrophomonas maltophilia] | GO:0004784;GO:0046872 |  |
| *S.maltophilia.CGMCC.1.1788GL002798* | CC:integral component of membrane;MF:phosphorelay sensor kinase activity; | GO:0016021;GO:0000155 |  |
| *S.maltophilia.CGMCC.1.1788GL000798* | MF:thymidine phosphorylase activity; | GO:0009032 |  |
| *S.maltophilia.CGMCC.1.1788GL004090* | BP:proteolysis;MF:metal ion binding;MF:metalloendopeptidase activity; | GO:0006508;GO:0046872;GO:0004222 |  |
| *S.maltophilia.CGMCC.1.1788GL003780* | MF:potassium ion binding;MF:magnesium ion binding;MF:pyruvate kinase activity;MF:kinase activity;MF:ATP binding; | GO:0030955;GO:0000287;GO:0004743;GO:0016301;GO:0005524 |  |
| *S.maltophilia.CGMCC.1.1788GL001895* | BP:alanine biosynthetic process;BP:pantothenate biosynthetic process;CC:cytoplasm;MF:aspartate 1-decarboxylase activity; | GO:0006523;GO:0015940;GO:0005737;GO:0004068 |  |
| *novel0021* | acetyl-CoA carboxylase biotin carboxylase subunit, partial [Stenotrophomonas sp. KAs 5-3] | GO:0046872;GO:0016874;GO:0005524 |  |
| *novel0020* | 3-methylcrotonyl-CoA carboxylase, partial [Bacillus sp. Nf3] | GO:0046872;GO:0016874;GO:0005524 |  |
| *novel0025* | MULTISPECIES: isovaleryl-CoA dehydrogenase [Stenotrophomonas] | GO:1901565;GO:0003995;GO:0050660 |  |
| *S.maltophilia.CGMCC.1.1788GL002665* | MF:protein histidine kinase activity; | GO:0004673 |  |
| *S.maltophilia.CGMCC.1.1788GL002206* | BP:copper ion transport;CC:integral component of membrane;CC:plasma membrane;MF:cation-transporting ATPase activity;MF:ATPase activity;MF:copper ion binding;MF:ATP binding; | GO:0006825;GO:0016021;GO:0005886;GO:0019829;GO:0016887;GO:0005507;GO:0005524 |  |
| *S.maltophilia.CGMCC.1.1788GL002202* | BP:glycolytic process;MF:glucokinase activity;MF:ATP binding;MF:glucose binding; | GO:0006096;GO:0004340;GO:0005524;GO:0005536 |  |
| *S.maltophilia.CGMCC.1.1788GL001794* | MF:acetyltransferase activity; | GO:0016407 |  |
| *S.maltophilia.CGMCC.1.1788GL000224* | BP:lipid catabolic process;MF:hydrolase activity; | GO:0016042;GO:0016787 |  |
| *novel0106* | hypothetical protein, partial [Corallococcus sp. AB049A] | GO:0006351;GO:0000428;GO:0000287;GO:0008270;GO:0003677;GO:0003899 |  |
| *novel0104* | DNA-directed RNA polymerase subunit beta [Stenotrophomonas maltophilia] | GO:0006351;GO:0000428;GO:0003899;GO:0032549;GO:0003677 |  |
| *novel0109* | hypothetical protein, partial [Enterobacter hormaechei] | GO:0006351;GO:0000428;GO:0000287;GO:0008270;GO:0003677;GO:0003899 |  |
| *S.maltophilia.CGMCC.1.1788GL004238* | BP:chorismate biosynthetic process;BP:aromatic amino acid family biosynthetic process;BP:cellular amino acid biosynthetic process;MF:3-dehydroquinate dehydratase activity; | GO:0009423;GO:0009073;GO:0008652;GO:0003855 |  |
| *S.maltophilia.CGMCC.1.1788GL002675* | MF:hydrolase activity; | GO:0016787 |  |
| *S.maltophilia.CGMCC.1.1788GL002674* | MF:hydrolase activity; | GO:0016787 |  |
| *S.maltophilia.CGMCC.1.1788GL002673* | MF:dioxygenase activity; | GO:0051213 |  |
| *S.maltophilia.CGMCC.1.1788GL002216* | BP:carbohydrate metabolic process;MF:hydrolase activity, hydrolyzing O-glycosyl compounds; | GO:0005975;GO:0004553 |  |
| *S.maltophilia.CGMCC.1.1788GL003487* | CC:plasma membrane;MF:metal ion binding;MF:4 iron, 4 sulfur cluster binding;MF:electron carrier activity; | GO:0005886;GO:0046872;GO:0051539;GO:0009055 |  |
| *S.maltophilia.CGMCC.1.1788GL002046* | BP:queuosine biosynthetic process;CC:cytoplasm;MF:S-adenosylmethionin;MF:transferase activity;MF:isomerase activity; | GO:0008616;GO:0005737;GO:0051075;GO:0016740;GO:0016853 |  |
| *S.maltophilia.CGMCC.1.1788GL002198* | BP:histidine biosynthetic process;CC:cytoplasm;MF:imidazoleglycerol-phosphate synthase activity;MF:lyase activity; | GO:0000105;GO:0005737;GO:0000107;GO:0016829 |  |
| *S.maltophilia.CGMCC.1.1788GL002196* | BP:glutamine metabolic process;BP:histidine biosynthetic process;CC:cytoplasm;MF:hydrolase activity;MF:glutaminase activity;MF:lyase activity;MF:imidazoleglycerol-phosphate synthase activity; | GO:0006541;GO:0000105;GO:0005737;GO:0016787;GO:0004359;GO:0016829;GO:0000107 |  |
| *S.maltophilia.CGMCC.1.1788GL002197* | BP:histidine biosynthetic process;CC:cytoplasm;MF:1; | GO:0000105;GO:0005737;GO:0003949 |  |
| *S.maltophilia.CGMCC.1.1788GL002194* | BP:histidine biosynthetic process;MF:pyridoxal phosphate binding;MF:histidinol-phosphate transaminase activity; | GO:0000105;GO:0030170;GO:0004400 |  |
| *S.maltophilia.CGMCC.1.1788GL002195* | BP:histidine biosynthetic process;CC:cytoplasm;MF:metal ion binding;MF:imidazoleglycerol-phosphate dehydratase activity;MF:histidinol-phosphatase activity; | GO:0000105;GO:0005737;GO:0046872;GO:0004424;GO:0004401 |  |
| *S.maltophilia.CGMCC.1.1788GL004195* | MF:ribonuclease activity; | GO:0004540 |  |
| *S.maltophilia.CGMCC.1.1788GL004246* | CC:integral component of membrane;MF:phosphatidate cytidylyltransferase activity;MF:nucleotidyltransferase activity;MF:transferase activity, transferring acyl groups; | GO:0016021;GO:0004605;GO:0016779;GO:0016746 |  |
| *S.maltophilia.CGMCC.1.1788GL002538* | BP:proteolysis;MF:serine-type endopeptidase activity; | GO:0006508;GO:0004252 |  |
| *S.maltophilia.CGMCC.1.1788GL001756* | MF:acetyltransferase activity; | GO:0016407 |  |
| *S.maltophilia.CGMCC.1.1788GL003904* | BP:leucine biosynthetic process;MF:2-isopropylmalate synthase activity; | GO:0009098;GO:0003852 |  |
| *S.maltophilia.CGMCC.1.1788GL003589* | BP:propionate metabolic process, methylcitrate cycle;MF:citrate dehydratase activity;MF:aconitate hydratase activity;MF:iron-sulfur cluster binding;MF:metal ion binding; | GO:0019679;GO:0047780;GO:0003994;GO:0051536;GO:0046872 |  |
| *S.maltophilia.CGMCC.1.1788GL002185* | BP:phosphorylation;BP:threonine biosynthetic process;CC:cytoplasm;MF:homoserine kinase activity;MF:ATP binding; | GO:0016310;GO:0009088;GO:0005737;GO:0004413;GO:0005524 |  |
| *S.maltophilia.CGMCC.1.1788GL002187* | MF:threonine synthase activity; | GO:0004795 |  |
| *S.maltophilia.CGMCC.1.1788GL000215* | MF:thiolester hydrolase activity; | GO:0016790 |  |
| *S.maltophilia.CGMCC.1.1788GL004249* | BP:protein dephosphorylation;CC:integral component of membrane;MF:protein tyrosine/serine/threonine phosphatase activity; | GO:0006470;GO:0016021;GO:0008138 |  |
| *S.maltophilia.CGMCC.1.1788GL003596* | BP:response to antibiotic;MF:aminoglycoside 6'-N-acetyltransferase activity; | GO:0046677;GO:0047663 |  |
| *S.maltophilia.CGMCC.1.1788GL003590* | BP:tricarboxylic acid cycle;CC:cytoplasm;MF:transferase activity, transferring acyl groups, acyl groups converted into alkyl on transfer;MF:citrate synthase activity; | GO:0006099;GO:0005737;GO:0046912;GO:0036440 |  |
| *S.maltophilia.CGMCC.1.1788GL003040* | BP:proteolysis;MF:serine-type peptidase activity; | GO:0006508;GO:0008236 |  |
| molecular_function | small molecule sensor activity | GO:0140299 |  |
| *S.maltophilia.CGMCC.1.1788GL000831* | MF:phosphorelay sensor kinase activity; | GO:0000155 |  |
| *S.maltophilia.CGMCC.1.1788GL002798* | CC:integral component of membrane;MF:phosphorelay sensor kinase activity; | GO:0016021;GO:0000155 |  |
| molecular_function | structural molecule activity | GO:0005198 |  |
| *S.maltophilia.CGMCC.1.1788GL002082* | BP:translation;CC:ribonucleoprotein complex;CC:ribosome;MF:structural constituent of ribosome;MF:RNA binding; | GO:0006412;GO:1990904;GO:0005840;GO:0003735;GO:0003723 |  |
| *S.maltophilia.CGMCC.1.1788GL000843* | BP:translation;CC:ribonucleoprotein complex;CC:ribosome;MF:structural constituent of ribosome; | GO:0006412;GO:1990904;GO:0005840;GO:0003735 |  |
| *S.maltophilia.CGMCC.1.1788GL000876* | BP:translation;CC:small ribosomal subunit;MF:structural constituent of ribosome;MF:rRNA binding; | GO:0006412;GO:0015935;GO:0003735;GO:0019843 |  |
| *S.maltophilia.CGMCC.1.1788GL000852* | BP:translation;CC:ribonucleoprotein complex;CC:ribosome;MF:structural constituent of ribosome;MF:tRNA binding; | GO:0006412;GO:1990904;GO:0005840;GO:0003735;GO:0000049 |  |
| molecular_function | transcription regulator activity | GO:0140110 |  |
| *S.maltophilia.CGMCC.1.1788GL002632* | MF:transcription factor activity, sequence-specific DNA binding;MF:sequence-specific DNA binding; | GO:0003700;GO:0043565 |  |
| *S.maltophilia.CGMCC.1.1788GL002806* | MF:transcription factor activity, sequence-specific DNA binding; | GO:0003700 |  |
| *S.maltophilia.CGMCC.1.1788GL004601* | BP:response to stimulus;BP:DNA-templated transcription, initiation;MF:sigma factor activity;MF:DNA binding; | GO:0050896;GO:0006352;GO:0016987;GO:0003677 |  |
| *S.maltophilia.CGMCC.1.1788GL002671* | BP:DNA-templated transcription, initiation;MF:sigma factor activity;MF:DNA binding; | GO:0006352;GO:0016987;GO:0003677 |  |
| *S.maltophilia.CGMCC.1.1788GL002180* | MF:transcription factor activity, sequence-specific DNA binding;MF:sequence-specific DNA binding; | GO:0003700;GO:0043565 |  |
| *S.maltophilia.CGMCC.1.1788GL002524* | MF:transcription factor activity, sequence-specific DNA binding; | GO:0003700 |  |
| molecular_function | translation regulator activity | GO:0045182 |  |
| *S.maltophilia.CGMCC.1.1788GL001603* | CC:cytoplasm;MF:translation elongation factor activity; | GO:0005737;GO:0003746 |  |
| molecular_function | transporter activity | GO:0005215 |  |
| *S.maltophilia.CGMCC.1.1788GL000534* | BP:ion transport;CC:cell outer membrane;CC:pore complex;MF:porin activity;MF:cobalamin-transporting ATPase activity; | GO:0006811;GO:0009279;GO:0046930;GO:0015288;GO:0015420 |  |
| *S.maltophilia.CGMCC.1.1788GL004626* | MF:efflux transmembrane transporter activity; | GO:0015562 |  |
| *S.maltophilia.CGMCC.1.1788GL004707* | CC:integral component of membrane;MF:transmembrane transporter activity; | GO:0016021;GO:0022857 |  |
| *S.maltophilia.CGMCC.1.1788GL000192* | CC:integral component of membrane;MF:transmembrane transporter activity; | GO:0016021;GO:0022857 |  |
| *S.maltophilia.CGMCC.1.1788GL002037* | CC:integral component of membrane;MF:ABC-type transmembrane transporter activity;MF:ATP binding; | GO:0016021;GO:0140359;GO:0005524 |  |
| *S.maltophilia.CGMCC.1.1788GL002724* | BP:nitrate assimilation;CC:integral component of membrane;MF:nitrate transmembrane transporter activity; | GO:0042128;GO:0016021;GO:0015112 |  |
| *S.maltophilia.CGMCC.1.1788GL000793* | CC:integral component of membrane;MF:transmembrane transporter activity; | GO:0016021;GO:0022857 |  |
| *S.maltophilia.CGMCC.1.1788GL001012* | BP:response to chemical;CC:integral component of plasma membrane;MF:transmembrane transporter activity; | GO:0042221;GO:0005887;GO:0022857 |  |
| *S.maltophilia.CGMCC.1.1788GL002206* | BP:copper ion transport;CC:integral component of membrane;CC:plasma membrane;MF:cation-transporting ATPase activity;MF:ATPase activity;MF:copper ion binding;MF:ATP binding; | GO:0006825;GO:0016021;GO:0005886;GO:0019829;GO:0016887;GO:0005507;GO:0005524 |  |
| *S.maltophilia.CGMCC.1.1788GL004141* | CC:integral component of membrane;MF:transmembrane transporter activity; | GO:0016021;GO:0022857 |  |
| *S.maltophilia.CGMCC.1.1788GL002598* | CC:membrane;CC:integral component of membrane;MF:transmembrane transporter activity; | GO:0016020;GO:0016021;GO:0022857 |  |
| *S.maltophilia.CGMCC.1.1788GL003876* | BP:protein transport;CC:integral component of membrane;CC:plasma membrane;MF:transmembrane transporter activity; | GO:0015031;GO:0016021;GO:0005886;GO:0022857 |  |
| *S.maltophilia.CGMCC.1.1788GL003875* | BP:protein transport;CC:integral component of membrane;CC:plasma membrane;MF:transmembrane transporter activity; | GO:0015031;GO:0016021;GO:0005886;GO:0022857 |  |
| *S.maltophilia.CGMCC.1.1788GL000362* | CC:integral component of plasma membrane;MF:potassium ion binding;MF:potassium-transporting ATPase activity; | GO:0005887;GO:0030955;GO:0008556 |  |
| KEGG analysis | | | |
| Gene_id | KO Description | KO ID | KEGG Name |
| Metabolism |  | Amino acid metabolism |  |
| *S.maltophilia.CGMCC.1.1788GL002185* | homoserine kinase [EC:2.7.1.39] | K00872 | thrB |
| *S.maltophilia.CGMCC.1.1788GL002187* | threonine synthase [EC:4.2.3.1] | K01733 | thrC |
| *S.maltophilia.CGMCC.1.1788GL002194* | histidinol-phosphate aminotransferase [EC:2.6.1.9] | K00817 | hisC |
| *S.maltophilia.CGMCC.1.1788GL002195* | imidazoleglycerol-phosphate dehydratase / histidinol-phosphatase [EC:4.2.1.19 3.1.3.15] | K01089 | hisB |
| *S.maltophilia.CGMCC.1.1788GL002196* | imidazole glycerol-phosphate synthase subunit HisH [EC:4.3.2.10] | K02501 | hisH |
| *S.maltophilia.CGMCC.1.1788GL002197* | phosphoribosylformimino-5-aminoimidazole carboxamide ribotide isomerase [EC:5.3.1.16] | K01814 | hisA |
| *S.maltophilia.CGMCC.1.1788GL002198* | imidazole glycerol-phosphate synthase subunit HisF [EC:4.3.2.10] | K02500 | hisF |
| *S.maltophilia.CGMCC.1.1788GL003354* | amino-acid racemase [EC:5.1.1.10] | K25316 | racX |
| *S.maltophilia.CGMCC.1.1788GL003898* | ketol-acid reductoisomerase [EC:1.1.1.86] | K00053 | ilvC |
| *S.maltophilia.CGMCC.1.1788GL003904* | 2-isopropylmalate synthase [EC:2.3.3.13] | K01649 | leuA |
| *S.maltophilia.CGMCC.1.1788GL004238* | 3-dehydroquinate dehydratase II [EC:4.2.1.10] | K03786 | aroQ |
| *novel0020* | 3-methylcrotonyl-CoA carboxylase alpha subunit [EC:6.4.1.4] | K01968 |  |
| *novel0021* | 3-methylcrotonyl-CoA carboxylase alpha subunit [EC:6.4.1.4] | K01968 |  |
| *novel0025* | isovaleryl-CoA dehydrogenase [EC:1.3.8.4] | K00253 | ivd |
| *novel0067* | asparagine synthase (glutamine-hydrolysing) [EC:6.3.5.4] | K01953 | asnB |
| Metabolism |  | Biosynthesis of other secondary metabolites |  |
| *S.maltophilia.CGMCC.1.1788GL002194* | histidinol-phosphate aminotransferase [EC:2.6.1.9] | K00817 | hisC |
| *S.maltophilia.CGMCC.1.1788GL002202* | glucokinase [EC:2.7.1.2] | K00845 | glk |
| Metabolism |  | Carbohydrate metabolism |  |
| *S.maltophilia.CGMCC.1.1788GL001956* | D-lactate dehydratase / protein deglycase [EC:4.2.1.130 3.5.1.124] | K05523 | hchA |
| *S.maltophilia.CGMCC.1.1788GL002202* | glucokinase [EC:2.7.1.2] | K00845 | glk |
| *S.maltophilia.CGMCC.1.1788GL003134* | fumarate hydratase, class I [EC:4.2.1.2] | K01676 | fumA |
| *S.maltophilia.CGMCC.1.1788GL003589* | 2-methylcitrate dehydratase (2-methyl-trans-aconitate forming) [EC:4.2.1.117] | K20455 | acnD |
| *S.maltophilia.CGMCC.1.1788GL003590* | 2-methylcitrate synthase [EC:2.3.3.5] | K01659 | prpC |
| *S.maltophilia.CGMCC.1.1788GL003780* | pyruvate kinase [EC:2.7.1.40] | K00873 | pyk |
| *S.maltophilia.CGMCC.1.1788GL003904* | 2-isopropylmalate synthase [EC:2.3.3.13] | K01649 | leuA |
| *S.maltophilia.CGMCC.1.1788GL003981* | alcohol dehydrogenase (NADP+) [EC:1.1.1.2] | K13979 | yahK |
| Metabolism |  | Energy metabolism |  |
| *S.maltophilia.CGMCC.1.1788GL000393* | heme o synthase [EC:2.5.1.141] | K02257 | ctaB |
| *S.maltophilia.CGMCC.1.1788GL002718* | sulfite reductase (NADPH) flavoprotein alpha-component [EC:1.8.1.2] | K00380 | cysJ |
| *S.maltophilia.CGMCC.1.1788GL002724* | MFS transporter, NNP family, nitrate/nitrite transporter | K02575 | narK |
| *S.maltophilia.CGMCC.1.1788GL002726* | nitrate reductase gamma subunit [EC:1.7.5.1 1.7.99.-] | K00374 | narI |
| *S.maltophilia.CGMCC.1.1788GL002728* | nitrate reductase / nitrite oxidoreductase, beta subunit [EC:1.7.5.1 1.7.99.-] | K00371 | narH |
| *S.maltophilia.CGMCC.1.1788GL002729* | nitrate reductase / nitrite oxidoreductase, alpha subunit [EC:1.7.5.1 1.7.99.-] | K00370 | narG |
| *S.maltophilia.CGMCC.1.1788GL003134* | fumarate hydratase, class I [EC:4.2.1.2] | K01676 | fumA |
| *novel0238* | nitrate reductase / nitrite oxidoreductase, alpha subunit [EC:1.7.5.1 1.7.99.-] | K00370 | narG |
| *novel0239* | nitrate reductase / nitrite oxidoreductase, alpha subunit [EC:1.7.5.1 1.7.99.-] | K00370 | narG |
| Metabolism |  | Global and overview maps |  |
| *S.maltophilia.CGMCC.1.1788GL001601* | uridylate kinase [EC:2.7.4.22] | K09903 | pyrH |
| *S.maltophilia.CGMCC.1.1788GL002202* | glucokinase [EC:2.7.1.2] | K00845 | glk |
| *novel0245* | ribonucleoside-diphosphate reductase alpha chain [EC:1.17.4.1] | K00525 | nrdA |
| Metabolism |  | Glycan biosynthesis and metabolism |  |
| *S.maltophilia.CGMCC.1.1788GL002216* | beta-mannosidase [EC:3.2.1.25] | K01192 | manB |
| *S.maltophilia.CGMCC.1.1788GL003554* | lipid A ethanolaminephosphotransferase [EC:2.7.8.43] | K03760 | eptA |
| Metabolism |  | Lipid metabolism |  |
| *S.maltophilia.CGMCC.1.1788GL003981* | alcohol dehydrogenase (NADP+) [EC:1.1.1.2] | K13979 | yahK |
| *S.maltophilia.CGMCC.1.1788GL004246* | phosphatidate cytidylyltransferase [EC:2.7.7.41] | K00981 | cdsA |
| Metabolism |  | Metabolism of cofactors and vitamins |  |
| *S.maltophilia.CGMCC.1.1788GL000393* | heme o synthase [EC:2.5.1.141] | K02257 | ctaB |
| *S.maltophilia.CGMCC.1.1788GL001895* | aspartate 1-decarboxylase [EC:4.1.1.11] | K01579 | panD |
| *S.maltophilia.CGMCC.1.1788GL002187* | threonine synthase [EC:4.2.3.1] | K01733 | thrC |
| *S.maltophilia.CGMCC.1.1788GL002732* | molybdenum cofactor guanylyltransferase [EC:2.7.7.77] | K03752 | mobA |
| *S.maltophilia.CGMCC.1.1788GL003683* | 7-carboxy-7-deazaguanine synthase [EC:4.3.99.3] | K10026 | queE |
| *S.maltophilia.CGMCC.1.1788GL003898* | ketol-acid reductoisomerase [EC:1.1.1.86] | K00053 | ilvC |
| *novel0156* | bacterioferritin [EC:1.16.3.1] | K03594 | bfr |
| Metabolism |  | Metabolism of other amino acids |  |
| *S.maltophilia.CGMCC.1.1788GL001895* | aspartate 1-decarboxylase [EC:4.1.1.11] | K01579 | panD |
| *S.maltophilia.CGMCC.1.1788GL003354* | amino-acid racemase [EC:5.1.1.10] | K25316 | racX |
| *S.maltophilia.CGMCC.1.1788GL004698* | glutathione peroxidase [EC:1.11.1.9] | K00432 | gpx |
| Metabolism |  | Nucleotide metabolism |  |
| *S.maltophilia.CGMCC.1.1788GL001601* | uridylate kinase [EC:2.7.4.22] | K09903 | pyrH |
| *novel0245* | ribonucleoside-diphosphate reductase alpha chain [EC:1.17.4.1] | K00525 | nrdA |
| Metabolism |  | Xenobiotics biodegradation and metabolism |  |
| *S.maltophilia.CGMCC.1.1788GL002940* | N-ethylmaleimide reductase [EC:1.-.-.-] | K10680 | nemA |
| Genetic Information Processing |  | Folding, sorting and degradation |  |
| *S.maltophilia.CGMCC.1.1788GL000873* | preprotein translocase subunit SecY | K03076 | secY |
| *S.maltophilia.CGMCC.1.1788GL002734* | sulfur-carrier protein | K03636 | moaD |
| *novel0277* | polyribonucleotide nucleotidyltransferase [EC:2.7.7.8] | K00962 | pnp |
| Genetic Information Processing |  | Transcription |  |
| *S.maltophilia.CGMCC.1.1788GL000846* | DNA-directed RNA polymerase subunit beta' [EC:2.7.7.6] | K03046 | rpoC |
| *S.maltophilia.CGMCC.1.1788GL000877* | DNA-directed RNA polymerase subunit alpha [EC:2.7.7.6] | K03040 | rpoA |
| *novel0104* | DNA-directed RNA polymerase subunit beta [EC:2.7.7.6] | K03043 | rpoB |
| *novel0105* | DNA-directed RNA polymerase subunit beta' [EC:2.7.7.6] | K03046 | rpoC |
| *novel0106* | DNA-directed RNA polymerase subunit beta' [EC:2.7.7.6] | K03046 | rpoC |
| *novel0109* | DNA-directed RNA polymerase subunit beta' [EC:2.7.7.6] | K03046 | rpoC |
| Genetic Information Processing |  | Translation |  |
| *S.maltophilia.CGMCC.1.1788GL000843* | large subunit ribosomal protein L7/L12 | K02935 | rplL |
| *S.maltophilia.CGMCC.1.1788GL000852* | small subunit ribosomal protein S10 | K02946 | rpsJ |
| *S.maltophilia.CGMCC.1.1788GL000876* | small subunit ribosomal protein S4 | K02986 | rpsD |
| *S.maltophilia.CGMCC.1.1788GL002082* | small subunit ribosomal protein S1 | K02945 | rpsA |
| Environmental Information Processing |  | Membrane transport |  |
| *S.maltophilia.CGMCC.1.1788GL000873* | preprotein translocase subunit SecY | K03076 | secY |
| *S.maltophilia.CGMCC.1.1788GL001652* | phosphate transport system substrate-binding protein | K02040 | pstS |
| Environmental Information Processing |  | Signal transduction |  |
| *S.maltophilia.CGMCC.1.1788GL000362* | potassium-transporting ATPase potassium-binding subunit | K01546 | kdpA |
| *S.maltophilia.CGMCC.1.1788GL000831* | two-component system, NarL family, capsular synthesis sensor histidine kinase RcsC [EC:2.7.13.3] | K07677 | rcsC |
| *S.maltophilia.CGMCC.1.1788GL001652* | phosphate transport system substrate-binding protein | K02040 | pstS |
| *S.maltophilia.CGMCC.1.1788GL002726* | nitrate reductase gamma subunit [EC:1.7.5.1 1.7.99.-] | K00374 | narI |
| *S.maltophilia.CGMCC.1.1788GL002728* | nitrate reductase / nitrite oxidoreductase, beta subunit [EC:1.7.5.1 1.7.99.-] | K00371 | narH |
| *S.maltophilia.CGMCC.1.1788GL002729* | nitrate reductase / nitrite oxidoreductase, alpha subunit [EC:1.7.5.1 1.7.99.-] | K00370 | narG |
| *S.maltophilia.CGMCC.1.1788GL002776* | superoxide dismutase, Fe-Mn family [EC:1.15.1.1] | K04564 |  |
| *S.maltophilia.CGMCC.1.1788GL002798* | two-component system, OmpR family, sensor kinase ParS [EC:2.7.13.3] | K18072 | parS |
| *S.maltophilia.CGMCC.1.1788GL004246* | phosphatidate cytidylyltransferase [EC:2.7.7.41] | K00981 | cdsA |
| *novel0238* | nitrate reductase / nitrite oxidoreductase, alpha subunit [EC:1.7.5.1 1.7.99.-] | K00370 | narG |
| *novel0239* | nitrate reductase / nitrite oxidoreductase, alpha subunit [EC:1.7.5.1 1.7.99.-] | K00370 | narG |
| Cellular Processes |  | Cell motility |  |
| *S.maltophilia.CGMCC.1.1788GL002305* | flagellar biosynthesis protein FlhB | K02401 | flhB |
| *S.maltophilia.CGMCC.1.1788GL002308* | flagellar biosynthesis protein FliR | K02421 | fliR |
| *novel0226* | flagellar hook-associated protein 1 | K02396 | flgK |
| Cellular Processes |  | Cellular community - prokaryotes |  |
| *S.maltophilia.CGMCC.1.1788GL000831* | two-component system, NarL family, capsular synthesis sensor histidine kinase RcsC [EC:2.7.13.3] | K07677 | rcsC |
| *S.maltophilia.CGMCC.1.1788GL000873* | preprotein translocase subunit SecY | K03076 | secY |
| *S.maltophilia.CGMCC.1.1788GL001200* | diguanylate cyclase [EC:2.7.7.65] | K18968 | adrA |
| *S.maltophilia.CGMCC.1.1788GL001955* | LuxR family transcriptional regulator, quorum-sensing system regulator BjaR1 | K18098 | bjaR1 |
| Cellular Processes |  | Transport and catabolism |  |
| *S.maltophilia.CGMCC.1.1788GL002216* | beta-mannosidase [EC:3.2.1.25] | K01192 | manB |
| *S.maltophilia.CGMCC.1.1788GL002776* | superoxide dismutase, Fe-Mn family [EC:1.15.1.1] | K04564 |  |
| *novel0012* | superoxide dismutase, Cu-Zn family [EC:1.15.1.1] | K04565 |  |
| Organismal Systems |  | Aging |  |
| *S.maltophilia.CGMCC.1.1788GL002776* | superoxide dismutase, Fe-Mn family [EC:1.15.1.1] | K04564 |  |
| *novel0012* | superoxide dismutase, Cu-Zn family [EC:1.15.1.1] | K04565 |  |
| Organismal Systems |  | Endocrine system |  |
| *S.maltophilia.CGMCC.1.1788GL003780* | pyruvate kinase [EC:2.7.1.40] | K00873 | pyk |
| *S.maltophilia.CGMCC.1.1788GL004698* | glutathione peroxidase [EC:1.11.1.9] | K00432 | gpx |
| Organismal Systems |  | Environmental adaptation |  |
| *S.maltophilia.CGMCC.1.1788GL000393* | heme o synthase [EC:2.5.1.141] | K02257 | ctaB |
| Human Diseases |  | Cancer: overview |  |
| *S.maltophilia.CGMCC.1.1788GL002776* | superoxide dismutase, Fe-Mn family [EC:1.15.1.1] | K04564 |  |
| *S.maltophilia.CGMCC.1.1788GL003780* | pyruvate kinase [EC:2.7.1.40] | K00873 | pyk |
| *novel0012* | superoxide dismutase, Cu-Zn family [EC:1.15.1.1] | K04565 |  |
| Human Diseases |  | Cardiovascular disease |  |
| *S.maltophilia.CGMCC.1.1788GL002776* | superoxide dismutase, Fe-Mn family [EC:1.15.1.1] | K04564 |  |
| Human Diseases |  | Drug resistance: antimicrobial |  |
| *S.maltophilia.CGMCC.1.1788GL002798* | two-component system, OmpR family, sensor kinase ParS [EC:2.7.13.3] | K18072 | parS |
| *S.maltophilia.CGMCC.1.1788GL003554* | lipid A ethanolaminephosphotransferase [EC:2.7.8.43] | K03760 | eptA |
| Human Diseases |  | Endocrine and metabolic disease |  |
| *S.maltophilia.CGMCC.1.1788GL003780* | pyruvate kinase [EC:2.7.1.40] | K00873 | pyk |
| Human Diseases |  | Infectious disease: bacterial |  |
| *S.maltophilia.CGMCC.1.1788GL001495* | filamentous hemagglutinin | K15125 | fhaB |
| *S.maltophilia.CGMCC.1.1788GL001652* | phosphate transport system substrate-binding protein | K02040 | pstS |
| Human Diseases |  | Infectious disease: viral |  |
| *S.maltophilia.CGMCC.1.1788GL003780* | pyruvate kinase [EC:2.7.1.40] | K00873 | pyk |
| Human Diseases |  | Neurodegenerative disease |  |
| *S.maltophilia.CGMCC.1.1788GL002776* | superoxide dismutase, Fe-Mn family [EC:1.15.1.1] | K04564 |  |
| *S.maltophilia.CGMCC.1.1788GL004698* | glutathione peroxidase [EC:1.11.1.9] | K00432 | gpx |
| *novel0012* | superoxide dismutase, Cu-Zn family [EC:1.15.1.1] | K04565 |  |
