# Supplementary material for: Stenotrophomonas maltophilia uses a c-di-GMP module to sense the mammalian body temperature during infection
Source: PLoS Pathog. 2024 Sep 4;20(9):e1012533. doi: 10.1371/journal.ppat.1012533 (PMC11404848; doi:10.1371/journal.ppat.1012533)
Supplement: S3 Table — (DOCX) [file ppat.1012533.s012.docx]

**S3 Table. Bacterial strains and plasmids used in this study.**

| **Strain or plasmid** | **Genotype or description** | **Source** |
| --- | --- | --- |
| **Strains** |  |  |
| *E. coli* DH5α | Host strain used for molecular cloning | Lab collection |
| *E. coli* BL21(DE3) | Host strain used for protein expression | Lab collection |
| *S. maltophilia* CGMCC 1.1788 | Wild type strain (WT) | Lab collection |
| WT-EV | *S. maltophilia* wild-type strain containing blank pBBR1MCS2 vector | This study |
| OX0244-HA | 00244 protein with HA tag overexpressed in *S. maltophilia* wild-type strain | This study |
| ΔbtsD-EV | In-frame deletion mutant of *btsD* containing blank pBBR1MCS2 vector | This study |
| CbtsD | Complementary strain with *btsD* overexpressed in Δ*btsD* mutant | This study |
| CbtsD^ΔSP^ | Complementary strain with *btsD* deleted signal peptide overexpressed in Δ*btsD* mutant | This study |
| CbtsD-His | Complementary strain with *btsD*-His overexpressed in Δ*btsD* mutant | This study |
| CbtsD^ΔSP^-His | Complementary strain with *btsD*-His deleted signal peptide overexpressed in Δ*btsD* mutant | This study |
| ΔbtsD-CbtsR | Complementary strain with *btsR* overexpressed in Δ*btsD* mutant | This study |
| ΔbtsD-CbtsR-His | Complementary strain with *btsR*-His overexpressed in Δ*btsD* mutan | This study |
| ΔbtsD-CbtsR^D58A^-His | Complementary strain with *btsR*^D58A^-His overexpressed in Δ*btsD* mutant | This study |
| ΔbtsD-EV-CbtsR-His | Complementary strain with *btsR*-His overexpressed in Δ*btsD*-EV | This study |
| ΔbtsD-EV-CbtsR^D58A^-His | Complementary strain with *btsR*^D58A^-His overexpressed in Δ*btsD*-EV | This study |
| CbtsD-CbtsR-His | Complementary strain with *btsR*-His overexpressed in C*btsD* | This study |
| CbtsD-CbtsR^D58A^-His | Complementary strain with *btsR*^D58A^-His overexpressed in C*btsD* | This study |
| ΔbtsD-Csod1-3 | Complementary strain with *02776-02778* gene cluster overexpressed in Δ*btsD* mutant | This study |
| CbtsD^ΔGGDEF^ | Complementary strain with *btsD* deleted GGDEF domain overexpressed in Δ*btsD* mutant | This study |
| CbtsD^ΔGGDEF^-CbtsR-His | Complementary strain with *btsR*-His overexpressed in C*btsD*^ΔGGDEF^ | This study |
| CbtsD^ΔGGDEF^-CbtsR^D58A^-His | Complementary strain with *btsR*^D58A^-His overexpressed in C*btsD*^ΔGGDEF^ | This study |
| ΔbtsK-EV | In-frame deletion mutant of *btsK* containing blank pBBR1MCS2 vector | This study |
| CbtsK | Complementary strain with *btsK* overexpressed in Δ*btsK* mutant | This study |
| CbtsK^H264A^-His | Complementary strain with *btsK*^H264A^-His overexpressed in Δ*btsK* mutant | This study |
| ΔbtsR-EV | Insertional inactivation mutant of *btsR* containing blank pBBR1MCS1 vector | This study |
| CbtsR | Complementary strain with *btsR* overexpressed in Δ*btsR* mutant | This study |
| CbtsR^D58A^ | Complementary strain with *btsR*^D58A^ overexpressed in Δ*btsR* mutant | This study |
| ΔbtsD-ΔbtsR-EV | Double in-frame deletion mutant of *btsD* and *btsR* containing blank pBBR1MCS2 vector | This study |
| Δ(btsD- btsR)-Csod1-3 | Complementary strain with *02776-02778* gene cluster overexpressed in Δ*btsD*-Δ*btsR* mutant | This study |
| Δ(btsD- btsR)-CbtsD | Complementary strain with *btsD* overexpressed in Δ*btsD*-Δ*btsR* mutant | This study |
| Δ(btsD- btsR)-CbtsR | Complementary strain with *btsR* overexpressed in Δ*btsD*-Δ*btsR* mutant | This study |
| Δ(btsD- btsR)-CbtsR^D58A^ | Complementary strain with *btsR*^D58A^ overexpressed in Δ*btsD*-Δ*btsR* mutant | This study |
| **Plasmids** |  |  |
| pK18mob | Suicide vector used in single-crossover recombination, Kan^r^ | This study |
| pK18mobsacB | Suicide vector used in double-crossover recombination, Kan^r^ | This study |
| pBBR1MCS2 | Broad-host-range vector used for genetic complementation, Kan^r^ | This study |
| pBBR1MCS1 | Broad-host-range vector used for genetic complementation, Chlo^r^ | This study |
| pET30a | Protein expression vector, Kan^r^ | This study |
| pGEX-6P-1 | Protein expression vector, Amp^r^ | This study |
| pK18mob::*btsR* | For insertional inactivation of *btsR* | This study |
| pK18mobsacB::*btsD* | For *btsD* in-frame deletion construction | This study |
| pK18mobsacB::*btsK* | For *btsK* in-frame deletion construction | This study |
| pK18mobsacB::*btsR* | For double in-frame deletion mutant of *btsD* and *btsR* construction | This study |
| pET30a-btsD | Protein expression vector, pET30a::btsD, expressing full-length BtsD | This study |
| pET30a-btsK | Protein expression vector, pET30a::btsK, expressing full-length BtsK | This study |
| pET30a-btsK^H264A^ | Protein expression vector, pET30a::btsK^H264A^,expressing BtsK protein with His264 mutated into Ala | This study |
| pET30a-btsR^D58A^ | Protein expression vector, pET30a::btsR^D58A^,expressing BtsR protein with Asp58 mutated into Ala | This study |
| pET30a-btsD^Δ(SP-GGDEF)^ | Protein expression vector, pET30a::btsD^ΔGGDEF^, expressing BtsD without signal peptide and GGDEF domain | This study |
| pET30a-btsR | Protein expression vector, pET30a::btsR, expressing BtsR without signal peptide domain | This study |
| pET30a-btsK^Δsensor^ | Protein expression vector, pET30a::btsK^Δsensor^, expressing BtsK without sensor domain | This study |
| pET30a-GGDEF^BtsD^ | Protein expression vector, pET30a::btsD-GGDEF, expressing the GGDEF domain of BtsD | This study |
| pET30a-WspR | Protein expression vector, pET30a::wspR, expressing WspR of *Pseudomonas aeruginosa* | This study |
| pET30a-BtsD^Δ(SP-FN3)^ | Protein expression vector, pET30a::btsD^Δ(SP-FN3)^, expressing the recombinant BtsD without its SP and FN3 domain | This study |
| pET30a-BtsD^sub^ | Protein expression vector, pET30a::btsD^sub^, expressing the recombinant BtsD with its GGDEF domain replaced by the GGDEF domain of WspR from *P. aeruginosa* | This study |
| pGEX-6P-1-DncV | Protein expression vector, pGEX-6P-1:: DncV, expressing full-length DncV | (Zhang *et al*, 2022) |
| pBBR1MCS2::*btsD* | Genetic complementation vector, pBBR1MCS2::*btsD* | This study |
| pBBR1MCS2::*btsD*-His | Genetic complementation vector, pBBR1MCS2::*btsD*-His | This study |
| pBBR1MCS2::*btsD*^ΔSP^-His | Genetic complementation vector, pBBR1MCS2::*btsD*^ΔSP^-His | This study |
| pBBR1MCS2::*btsD*^ΔGGDEF^ | Genetic complementation vector, pBBR1MCS2::*btsD*^ΔGGDEF^ | This study |
| pBBR1MCS2::*btsD*^ΔGGDEF^-His | Genetic complementation vector, pBBR1MCS2::*btsD*^ΔGGDEF^-His | This study |
| pBBR1MCS2::*btsK* | Genetic complementation vector, pBBR1MCS2::*btsK* | This study |
| pBBR1MCS2::*btsK*^H264A^ | Genetic complementation vector, pBBR1MCS2::*btsK*^H264A^ | This study |
| pBBR1MCS2::*btsR* | Genetic complementation vector, pBBR1MCS2::*btsR* | This study |
| pBBR1MCS1::*btsR* | Genetic complementation vector, pBBR1MCS1::*btsR* | This study |
| pBBR1MCS1::*btsR*-His | Genetic complementation vector, pBBR1MCS1::*btsR*-His | This study |
| pBBR1MCS2::*btsR*^D58A^ | Genetic complementation vector, pBBR1MCS2::*btsR*^D58A^ | This study |
| pBBR1MCS1::*btsR*^D58A^ | Genetic complementation vector, pBBR1MCS1::*btsR*^D58A^ | This study |
| pBBR1MCS1::*btsR*^D58A^-His | Genetic complementation vector, pBBR1MCS1::*btsR*^D58A^His | This study |
| pBBR1MCS2::*00244* | Genetic complementation vector, pBBR1MCS2::*00244* | This study |
| pBBR1MCS2::*sod1-3* | Genetic complementation vector, pBBR1MCS2::*sod1-3* | This study |
| Kan^r^ : kanamycin resistant; Amp^r^: Ampicillin resistant; CGMCC: China General Microbiological Culture Collection Center. |  |  |
|  | | |
