# Supplementary material for: Stenotrophomonas maltophilia uses a c-di-GMP module to sense the mammalian body temperature during infection
Source: PLoS Pathog. 2024 Sep 4;20(9):e1012533. doi: 10.1371/journal.ppat.1012533 (PMC11404848; doi:10.1371/journal.ppat.1012533)
Supplement: S4 Table — (DOCX) [file ppat.1012533.s013.docx]

**S4 Table. Primers used in this study.**

| **Primer** | **Sequence (5′-3′)** | **Description** |
| --- | --- | --- |
| ΔbtsD | W: CCCAAGCTTGTGGAGCGCCGCGTTTCG | For Δ*btsD* construction |
|  | X: CACCACCAGCCCAGCCAGCGCGCGCTTGAGCACGCC |  |
|  | Y: GGCGTGCTCAAGCGCGCGCTGGCTGGGCTGGTGGTG |  |
|  | Z: CCGGAATTCTCAACCAATACTCGTACG |  |
| CbtsD | F: CCCAAGCTTATGGGCAGGTCAGGGGAC | For *btsD* complementation |
|  | R: CCGGAATTCTCAACCAATACTCGTACG |  |
| Test-ΔbtsD | F: GGGGACAACGGGTGCTGG | For Δ*btsD* verification |
|  | R: CGCCATGCGTGGATGCGA |  |
| CbtsD^ΔSP^ | F: CCCAAGCTTGTGGCGCCGCCGCTGCGC | For *btsD*^ΔSP^ complementation |
|  | R: CCGGAATTCTCAACCAATACTCGTACG |  |
| CbtsD^ΔSP^-His | F: CCCAAGCTTGTGGCGCCGCCGCTGCGC | For *btsD*^ΔSP^-His complementation |
|  | R: CGAGCTCTCAGTGATGGTGATGATGGTGACCAATACTCGTACGATC |  |
| CbtsD^ΔGGDEF^-His | F: GGTACCATGGGCAGGTCAGGGGAC | For *btsD*^ΔGGDEF^-His complementation |
|  | R: GAGCTCTCAGTGATGGTGATGATGGTGGCGCTCGGCCAGCTCGGTCT |  |
| CbtsD^ΔGGDEF^ | F: GGTACCATGGGCAGGTCAGGGGAC | For *btsD*^ΔGGDEF^ complementation |
|  | R: GAGCTCTCAGCGCTCGGCCAGCTCGGTCT |  |
| Pro-His-BtsD | F: GGAATTCCATATGGCGCCGCCGCTGCGCGAC | For BtsD^ΔSP^ expression |
|  | R: CCCAAGCTTACCAATACTCGTACGATC |  |
| Pro-His-BtsD^Δ(SP-GGDEF)^ | F: GGAATTCCATATGGCGCCGCCGCTGCGCGAC | For BtsD(^ΔSP-GGDEF)^ expression |
|  | R: AAGCTTGCGCTCGGCCAGCTCGGTCTT |  |
| Pro-His-GGDEF | F: CATATGCTTGCACGGCAGGCCGAG | For GGDEF^BtsD^ expression |
|  | R: AAGCTTACCAATACTCGTACGATC |  |
| Pro-WspR | F: GGAATTCCATATGCACAACCCTCATGAGAGCAA  R: CCCAAGCTTGCCCGCCGGGGCCGGCGGC | For WspR expression |
| Pro-BtsD^Δ(SP-FN3)^ | W: GGAATTCCATATGGCGCCGCCGCTGCGCGAC | For BtsD^Δ(SP-FN3)^ expression |
|  | X: GGCTTCCTGCTGGCCGCGCTCGCGGAAGCGCT |  |
|  | Y: AGCGCTTCCGCGAGCGCGGCCAGCAGGAAGCC |  |
|  | Z: CCCAAGCTTACCAATACTCGTACGATC |  |
| Pro-BtsD^sub^ | W: TGAGTTACCTGATGTCGGACCG | For BtsD^sub^ expression |
|  | X: GTTGGTCTCCAGCAAGCGCTCGGCCAGCTC |  |
|  | Y: GAGCTGGCCGAGCGCTTGCTGGAGACCAAC |  |
|  | Z: CCCAAGCTTGCCCGCCGGGGCCGGCGGC |  |
| CbtsD-His | F: TGAGTTACCTGATGTCGGAC | For *btsD*-His complementation |
|  | R: CGAGCTCTCAGTGATGGTGATGATGGTGACCAATACTCGTACGATC |  |
| ΔbtsK | W: GAATTCATGAAGCGCCTGCGCCATTT | For Δ*btsK* construction |
|  | X: GCGCTGCTCGGCGAAGGCCGGATCA |  |
|  | Y: TGATCCGGCCTTCGCCGAGCAGCGC |  |
|  | Z: AAGCTTTCAGCCGGGCCGGCCA |  |
| Test-ΔbtsK | F: TGGCCTCCTCGGTGAACCTG | For Δ*btsK* verification |
|  | R: GATGCGCAGATGATAGGCCA |  |
| CbtsK | F: GGTACCATGAAGCGCCTGCGCCATTT | For *btsK* complementation |
|  | R: GAATTCTCAGCCGGGCCGGCCAGGCGGCA |  |
| CbtsK-His | F: GGTACCATGAAGCGCCTGCGCCATT | For *btsK*-His complementation |
|  | R: GAGCTCTCAGTGATGGTGATGATGGTGGCCGGGCCGGCCAGGCGGCA |  |
| CbtsK^H264A^-His | W: GGTACCATGAAGCGCCTGCGCCATT | For *btsK*^H264A^-His complementation |
|  | X: CGGGGTCTGCAGGTCAGCGGTGATCGCCGCCAGCA |  |
|  | Y: TGCTGGCGGCGATCACCGCTGACCTGCAGACCCCG |  |
|  | Z: GAGCTCTCAGTGATGGTGATGATGGTGGCCGGGCCGGCCAGGCGGCA |  |
| Pro-His-BtsK | F: CATATGAAGCGCCTGCGCCATTT | For BtsK expression |
|  | R: AAGCTTGCCGGGCCGGCCAGGCGGCA |  |
| Pro-His-BtsK^Δsensor^ | F: CATATGACCTCCAAACGCCAGCAGGA | For BtsK^Δsensor^ expression |
|  | R: AAGCTTGCCGGGCCGGCCAGGCGGCA |  |
| Pro-His-BtsK^H264A^ | W: CATATGAAGCGCCTGCGCCATTT | For BtsK^H264A^ expression |
|  | X: CGGGGTCTGCAGGTCAGCGGTGATCGCCGCCAGCA |  |
|  | Y: TGCTGGCGGCGATCACCGCTGACCTGCAGACCCCG |  |
|  | Z: AAGCTTGCCGGGCCGGCCAGGCGGCA |  |
| ΔbtsR | F: GAATTCACCGCCATGCGCCAGCTG | For Δ*btsR* construction |
|  | R: AAGCTTCTGCAGGTCGATCGCGCG |  |
| Test-ΔbtsR | F: TTGTAGGCGTCGATGACGCCGGTG | For Δ*btsR* verification |
|  | R: AACGGAACGAGGTGAAGTGCACAT |  |
| CbtsR | F: GGTACCATGGAGACTGAAAACACG | For *btsR* complementation |
|  | R: GAATTCTTATTCCAGGTTCACCGA |  |
| CbtsR^D58A^ | W: CATATGGAGACTGAAAACACG | For *btsR*^D58A^ complementation |
|  | X: CGCGGCAGGTTGAGGGCAAGCACGATCAGG |  |
|  | Y: ACCTGATCGTGCTTGCCCTCAACCTGCCGCG |  |
|  | Z: GAATTCTTATTCCAGGTTCACCGA |  |
| Pro-His-BtsR | F: CATATGGAGACTGAAAACACG | For BtsR expression |
|  | R: AAGCTTTTCCAGGTTCACCGAG |  |
| Pro-His-BtsR^D58A^ | W: CATATGGAGACTGAAAACACG | For BtsR^D58A^ expression |
|  | X: CGCGGCAGGTTGAGGGCAAGCACGATCAGG |  |
|  | Y: ACCTGATCGTGCTTGCCCTCAACCTGCCGCG |  |
|  | Z: AAGCTTTTCCAGGTTCACCGAG |  |
| CbtsR-His | F: GGTACCATGGAGACTGAAAACACGA | For *btsR*-His complementation |
|  | R: GAGCTCTTAGTGATGGTGATGATGGTGTTCCAGGTTCACCGAGGA |  |
| CbtsR^D58A^-His | W: CATATGGAGACTGAAAACACG | For *btsR*^D58A^-His complementation |
|  | X: CGCGGCAGGTTGAGGGCAAGCACGATCAGG |  |
|  | Y: ACCTGATCGTGCTTGCCCTCAACCTGCCGCG |  |
|  | Z: GAGCTCTTAGTGATGGTGATGATGGTGTTCCAGGTTCACCGAGGA |  |
| Promoter-2776 | F: CCGGTGAGGTTCGAAAAGCG | For PCR of *sod1-3* promoter |
|  | R: TGAAGCAATCTCCGTGGGGA |  |
| ChIP-2776 | F: CTTGACCTCAACCAATGTTG | For ChIP-qPCR of *sod1-3* promoter |
|  | R: TGAAGCAATCTCCGTGGGGA |  |
| Csod1-3 | F: CCAAGCTTATGTCGTACTCGCTTCC | For complementation of *sod1-3* gene cluster |
|  | R: CGGAATTCTCAGCCCGCTTTCGCC |  |
|  |  |  |
| 1786-btsR | a1: TGACCAGCTTGTAGGCGTC | For qRT-PCR of *orf* 0*1639*-*btsR* |
|  | a2: TGCGGATGTCGTTGTCGTC |  |
| btsR-btsK | b1: TGCCAACAAGGTGCTGTCGCGCGAA | For qRT-PCR of *btsR*-*btsK* |
|  | b2: TTCAGGTTCTGCCGCTCGAACTCCT |  |
| btsK-btsD | c1: GAAACCTCGCGTTCGCGG | For qRT-PCR of *btsK*-*btsD* |
|  | c2: ATTGCGCGAGGTCCACAC |  |
| btsD-1790 | d1: TGACCTTCAGCGCAGGCCTTGTGGA | For qRT-PCR of *btsD*- *01643* |
|  | d2: GTTCAGCGTGTTCGACACCGTCACC |  |
| FB-1786 | F: CTGGACGAATTCTCGTTG | For qRT-PCR of *01639* |
|  | R: GTGGTCAAGATCAAGGC |  |
| FB-btsR | F: CCCATGCCAACAAGGTGCT | For qRT-PCR of *btsR* |
|  | R: ATTCCAGGTTCACCGAGGAGG |  |
| FB-btsK | F: GACTGCTTGAGATCGGTGGG | For qRT-PCR of *btsK* |
|  | R: CAGATCCTGCAGCTTGGGAA |  |
| FB-btsD | F: GTCTGGCGGTGAGCTATGTG | For qRT-PCR of *btsD* |
|  | R: CACGTGCAGGGTGTAGTCAC |  |
| FB-1790 | F: GTCTGTTCGGTGTCATAC | For qRT-PCR of 01643 |
|  | R: GCAATCCGTTCCATCC |  |
| OX0244-HA | F: GGTACCATGGAATCCATGTCAACGAC | For overexpress *00244* with HA tag |
|  | R: GAATTCTCAAGCGTAGTCTGGGACGTCGTATGGGTAACGCGCCGCGATTC |  |
| tmRNA | -new F: AACTCTAGTTGCCAACGACG | For qRT-PCR of tmRNA |
|  | -new R: AAGGCACTCCATCCCCAGCA |  |
